# Toward Symmetric Organic Aqueous Flow Batteries: Triarylamine‐Based Bipolar Molecules and Their Characterization via an Extended Koutecký–Levich Analysis

**DOI:** 10.1002/chem.202500815

**Published:** 2025-05-03

**Authors:** Carlo Caianiello, Tim Tichter, Luis F. Arenas, René Wilhelm

**Affiliations:** ^1^ Institute of Organic Chemistry, Clausthal University of Technology Leibnizstraße 6 38678 Clausthal‐Zellerfeld Germany; ^2^ Bundesanstalt für Materialforschung und ‐prüfung BAM Unter den Eichen 87 12205 Berlin Germany; ^3^ Institute of Chemical and Electrochemical Process Engineering Clausthal University of Technology Leibnizstraße 17 38678 Clausthal‐Zellerfeld Germany; ^4^ Research Center for Energy Storage Technologies Clausthal University of Technology Am Stollen 19A 38640 Goslar Germany; ^5^ Electrochemical Engineering Laboratory Department of Mechanical Engineering University of Southampton Southampton SO17 1BJ UK; ^6^ School of Chemistry and Chemical Engineering University of Southampton Southampton SO17 1BJ UK

**Keywords:** bipolar redox‐active organic molecules, koutecký–levich analysis, symmetric organic flow batteries, zincke reaction

## Abstract

Symmetric organic flow batteries (SOFBs) can potentially address membrane crossover problems by employing bipolar redox‐active organic molecules (BROMs). Herein, a triarylamine (TAA) skeleton was chosen as a posolyte moiety for a new class of bipolar molecules for pH‐neutral aqueous flow batteries (FBs). Pyridinium and viologen derivatives were tethered to the posolyte moiety, and the new compounds were characterized. Cyclic voltammetry revealed that only viologen with a highly hydrophilic substituent, connected to the TAA moiety via a Zincke reaction, could be reversibly reduced. Varying the supporting electrolyte concentration on the selected derivative revealed water solubility as a challenge for further development. The selected derivative, MeO‐TPA‐Vi‐DMAE, was subjected to hydrodynamic voltammetry, and a modified Koutecký–Levich analysis was developed to investigate the observed potential‐dependent currents at the hydrodynamically dominated region, which are often seen with redox‐active organic molecules. This model discarded a purely Ohmic effect, showing a useful Levich slope at a certain overpotential before the onset of a secondary reaction. TAA‐based BROMs hold promise for pH‐neutral aqueous SOFBs, and the results will guide the design of new derivatives. The three‐term Koutecký–Levich relation here introduced will be useful not only to develop BROM‐based FBs but will most likely appeal to a much broader audience.

## Graphical abstract

Bipolar redox‐active organic molecules based on a triarylamine skeleton and a viologen unit for pH‐neutral aqueous flow batteries are presented. Cyclic voltammetry shows that only viologen with a highly hydrophilic substituent, connected to the TAA moiety can be reversibly reduced. A modified Koutecký–Levich analysis is presented to investigate the observed potential‐dependent currents at the hydrodynamically dominated region.

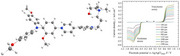

## 1 Introduction

Reliable and efficient stationary energy storage is essential for mitigating the intermittence of renewable energy sources such as solar and wind power.^1^ Flow batteries (FBs) have emerged as a promising energy storage technology, capable of discharging over a timescale of several hours.^[^ ^1^, ^2^, ^3^ ^]^ In an FB, energy is stored within redox‐active substances dissolved in liquid electrolytes, allowing for the decoupling of power and capacity.^[^ ^4^, ^5^ ^]^ Additionally, aqueous FBs (AFBs) offer nonflammability, thus a safer operation, which neutral pH electrolytes can enhance.^[^ ^3^, ^6^ ^]^ Presently, the most advanced system is the vanadium FB, which has been commercially available for more than two decades. An increasing number of such FBs are being deployed worldwide.^[^ ^7^ ^]^ However, the high and volatile costs of vanadium hinder broader commercialization.^[^ ^1^ ^]^ To address this challenge, aqueous organic flow batteries (AOFBs) based on redox‐active organic molecules (ROMs) have gained attention as a possible alternative. AOFBs may benefit from fewer supply limitations, while ROMs offer several advantages, such as fine‐tuning their properties and reliance on abundant elements.^[^ ^8^, ^9^ ^]^ ROMs are typically divided into negative redox couples (for the negolyte) and positive redox couples (for the posolytes).^[^ ^10^ ^]^ Unlike the acidic vanadium FBs, the supporting electrolytes for AOFBs are found over the whole pH range, from acidic to alkaline.^[^ ^11^ ^]^ However, neutral AOFBs are particularly interesting due to their inherently stable nature as well as for improved balance of plant safety and durability.^[^ ^11^, ^12^, ^13^ ^]^

In contrast to vanadium FBs, electrolyte cross‐contamination can be problematic in AOFBs due to the bidirectional transport of active species through the membrane. This “crossover” is difficult to avoid and can be irreversible in most OFBs employing different redox species in the posolyte and negolyte.^[^ ^14^, ^15^ ^]^ Among possible strategies for mitigating crossover, the concept of symmetric organic flow batteries (SOFBs) represents an attractive approach.^[^ ^15^ ^]^ An SOFB employs the same molecule on both half‐cells (i.e., a so‐called bipolar redox‐active organic molecule or BROM). Thus, crossover between the two sides does not lead to the mixing of distinct chemical species, meaning that it is less likely to result in permanent loss of battery capacity. Moreover, by using BROMs, neither chemical nor electrical potential gradients are present at the discharged state between the two half‐cells, making the driving force for crossover or self‐discharge reactions negligible.^[^ ^15^ ^]^ The latter being a peculiar advantage of BROMs, as other strategies, such as the use of thicker membranes^[^ ^16^ ^]^ or larger substituents on ROMs,^[^ ^17^, ^18^ ^]^ will always imply the use of two different molecules for the two half‐cells. Furthermore, in an SOFB, cell polarities can be reversed during cycling, thus extending the electrolyte lifetime if the degradation takes place preferentially in one half‐cell.^[^ ^15^ ^]^

**Scheme 1:**
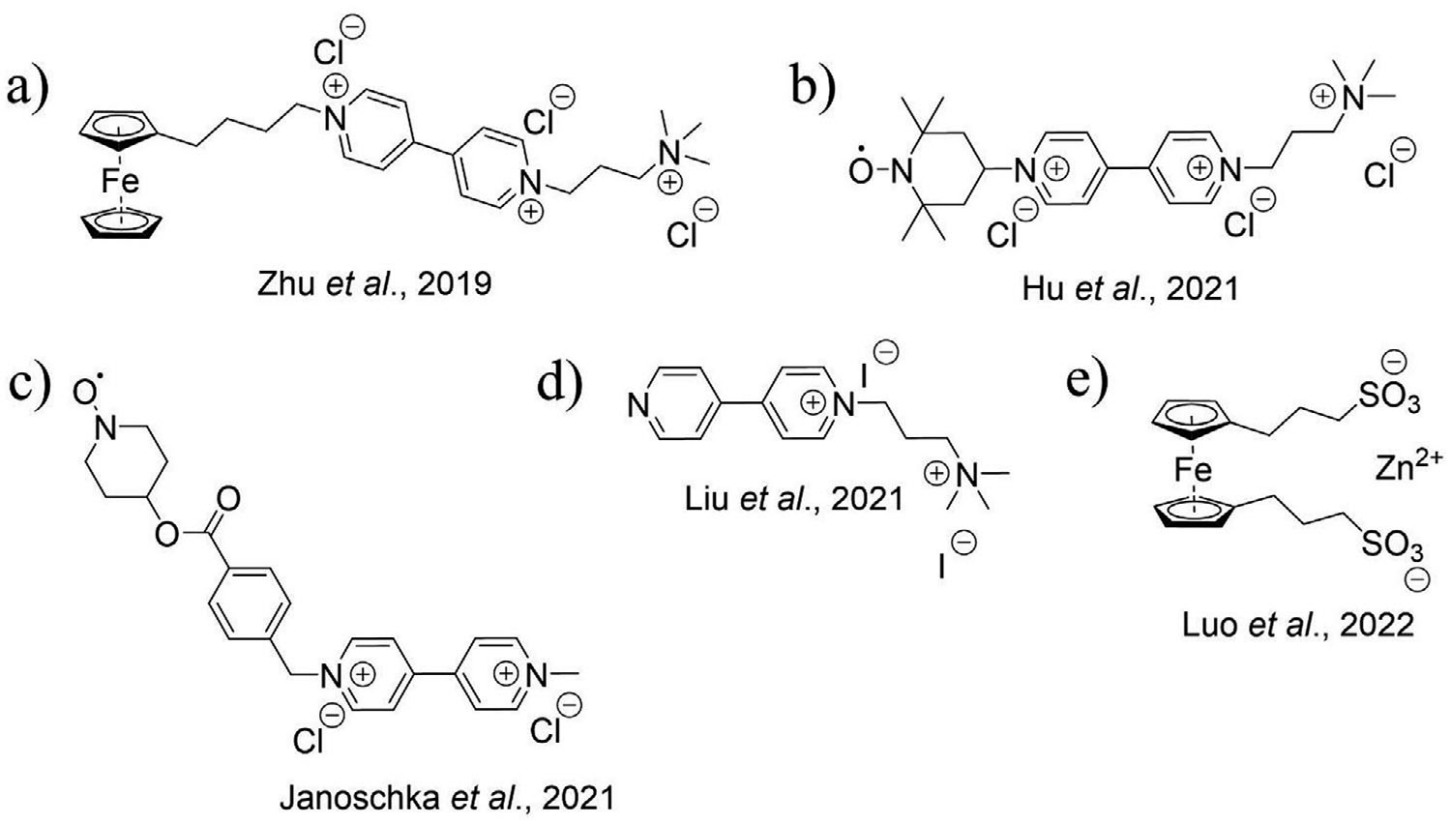
Known bipolar molecules for AFBs; a) Work from Ref. [19]; b) Work from Ref. [20]; c) Work from Ref. [21]; d) Work from Ref. [22]; e) Work from Ref. [23]

The SOFB strategy has been more commonly investigated for nonaqueous FBs,^[^ ^15^, ^24^ ^–^ ^27^ ^]^ with only a few examples of water‐soluble BROMs. For instance, Zhu et al. have reported a ferrocene‐viologen BROM (Scheme 1a),^[^ ^19^ ^]^ showing stability over 4000 cycles with no detectable decomposition and 75% capacity retention. Still, its OCV of only 0.7 V highlights the need to modify the ferrocene moiety to increase cell voltage. Wang et al. have reported a TEMPO‐viologen derivative BROM (see Scheme 1, b),^[^ ^20^ ^]^ demonstrating how to combine two ROMs into one BROM; nevertheless, the use of simple TEMPO as the posolyte moiety is not ideal due to its known instability.^[^ ^28^, ^29^ ^]^ Another example of a TEMPO‐viologen BROM is the seminal work by Janoschka et al. in which a benzyl linker was adopted (Scheme 1c).^[^ ^21^ ^]^ However, the low solubility of this BROM underlines the need to boost the water solubility in all of its redox states. Liu et al. adopted a different strategy by using a bipolar mono‐N‐alkylated bipyridinium iodide salt as a trifunctional electroactive compound (see Scheme 1d), namely, negolyte, posolyte, and supporting electrolyte.^[^ ^22^ ^]^ However, the iodide counter anion requires careful electrolyte optimization to avoid deposition at the electrode. A special case is the zinc‐ferrocene aqueous FB reported by Luo et al., where the BROM consisted of zinc 1,1′‐bis(3‐sulfonatopropyl)ferrocene in which the ferrocene acted as the posolyte moiety and zinc as the negolyte moiety (Scheme 1e).^[^ ^23^ ^]^ However, the FB suffered from zinc dendrite formation at high concentrations (e.g., at 1.0 M of BROM) and high current densities (0.4 and 0.33 A cm^−2^). In summary, stable and fully water‐soluble BROMs with useful cell voltages are yet to be developed.

Aiming to advance the aqueous SOFB strategy, a class of BROMs is presented for the first time by combining common negolyte moieties, that is, pyridinium or viologen derivatives, with a recent class of posolyte moieties, that is, triarylamines (TAAs). TAAs have lately gained attention as posolyte redox couples for AOFBs because they are easy to modify electron‐rich aromatic molecules, yet only a few examples have been reported.^[^ ^30^, ^31^ ^]^ Several modifications of the TAA core have been screened in our laboratory through the use of simple organic building blocks and common synthetic procedures. The two moieties were tethered together either via a cross‐coupling or Zincke reaction to yield new BROMs. Tethering water‐soluble moieties, such as pyridinium or viologen derivatives, to the insoluble TAA core allows the exploration of new posolyte moieties for both AOFBs and SOFBs. This is crucial, as usually the posolyte limits their performance and the library of published posolytes for aqueous systems is rather limited compared to negolytes. In addition, properly substituted TAAs have been recently proven to give stable radicals upon oxidation (i.e., upon charging), and thus stable posolytes in nonaqueous systems.^[^ ^32^ ^]^ The latter result might translate to aqueous conditions as preliminary evidenced by Wang et al.^[^ ^30^ ^]^

The electrochemical characterization of ROMs for AOFBs is usually performed through cyclic voltammetry at quiescent electrolytes and by hydrodynamic voltammetry at a rotating disc electrode (RDE). These techniques permit the evaluation of chemical and electrochemical stability, relevant potentials, type of reaction (reversible, quasi‐reversible, irreversible), and, if possible, the kinetics of the reaction of interest.^[^ ^33^, ^34^ ^]^ In particular, limiting currents obtained at RDE allow for the estimation of diffusion coefficients. Additionally, if the system is quasi‐reversible or irreversible, the application of a Koutecký–Levich analysis can extract kinetic constants from the mixed control region of LSV curves.^[^ ^34^ ^]^ However, the studied reactions frequently fall outside the ideal case described by the classical theory. Thus, modifications to the Koutecký–Levich relation have been developed to account for multistep reaction mechanisms and electrode inhomogeneities,^[^ ^35^ ^]^ micro‐ and nanoparticle catalyst‐coated electrodes,^[^ ^36^, ^37^ ^]^ thin film‐coated electrodes,^[^ ^33^ ^]^ and finite electrode transfer kinetics.^[^ ^38^ ^]^ A nonideality seen in some RDE studies of ROMs, and the partial focus of this work, is a potential‐dependent current observed at high overpotentials in the mass transfer dominated region, sometimes described–incorrectly– as “limiting currents with a slope” or “potential‐dependent limiting currents”. These are, in fact often reported and overlooked, for example, Refs,^[^ ^12^, ^30^, ^31^, ^39^, ^40^, ^41^, ^42^, ^43^ ^]^ with their possible causes and implications usually dismissed. It is worth noting that, in the context of hydrodynamic voltammetry, the strict definition of limiting current implies mass transfer control and independence from overpotential.

The present work discusses the feasibility of TAA as a possible positive redox couple posolyte core structure for pH‐neutral aqueous SOFBs posolytes. The synthesis of several new TAA‐based BROMs is presented, followed by a discussion of the key characteristics of the new electrolytes for the target application as evaluated by cyclic voltammetry at a glassy carbon electrode. One particular TAA‐based BROM, MeO‐TAA‐Vi‐DMAE, is selected and further studied through hydrodynamic voltammetry. Peculiarly shaped current‐potential curves with potential‐dependent current at high overpotentials in the mass transfer dominated region are obtained. Thus, a theoretical concept is introduced by invoking Ohmic limitations in addition to electrode kinetics and hydrodynamics. This leads to an extended Koutecký–Levich equation with a third, yet potential‐dependent term. In this manner, it was possible to study the possible contribution of an electrolyte Ohmic effect to the mass transfer dominated, nonideal limiting currents and to assess if a Levich slope could be useful to determine the diffusion coefficient because of a secondary reaction onset. The herein presented Koutecký–Levich relation will not only shed light on a new class of the next generation of BROMs but will also appeal to a much broader audience in electroanalysis.

## 2 Results and Discussion

### 2.1 Synthesis and Cyclic Voltammetry of New Bipolar Pyridinium‐BMTAA Derivatives

Preliminary results (see Starting point section in Supporting Information) and the scarcity of reports in the literature motivated us to further investigate TAA as a posolyte core structure. To the best of our knowledge, there are only a couple of examples involving TAA‐based posolytes in AOFBs. Among the latter, Farag et al. reported the use of tris(4‐aminophenyl)amine as a possible posolyte in a mixed supporting electrolyte (H_3_PO_4_/HCl 1:1) with VCl_2_/VCl_3_ as the negolyte.^[^ ^31^ ^]^ Another example originates from the work of Wang et al. in which 4,4′,4″‐trihydroxytriphenylamine was used as the posolyte with zinc as the negolyte in an aqueous solvent (5:1 H_2_O/DMF at pH 5).^[^ ^30^ ^]^ The latter work showed that adding methoxy groups in the *para* position could stabilize the radical cation and prevent typical side reactions such as radical quenching from the aqueous solvent. In addition, electron‐donating groups (EDGs) can be used to further tune the oxidation potential.

Considering our previous experience with viologens and push‐pull dyes,^[^ ^18^, ^44^, ^45^, ^46^ ^]^ our attention turned initially to the use of pyridinium as a possible dual‐function water‐solubilizing and electroactive group to produce a new series of BROMs featuring a TAA derivative as the posolyte moiety. In particular, by applying the strategy proposed by Wang et al.,^[^ ^30^ ^]^ the chosen moiety as the posolyte side is the 4‐[bis(4‐methoxyphenyl)amino]phenyl (BMTAA). The addition of a strong electron‐withdrawing group (e.g., pyridinium or 4,4´‐bipyridinium) to a π‐donor moiety, such as BMTAA, results in a typical push‐pull structure with potentially interesting electrochemical and photophysical properties.^[^ ^47^ ^]^

**Figure 1:**
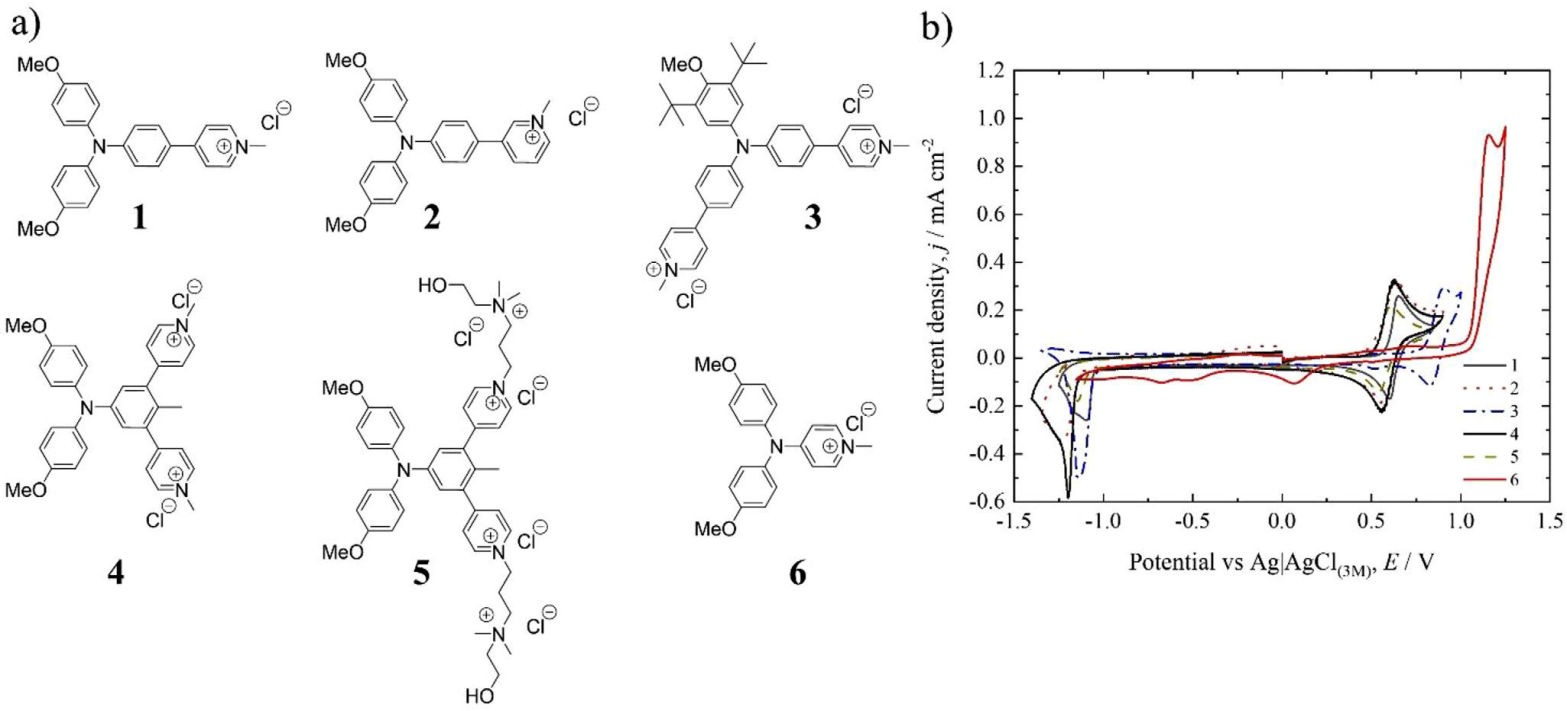
New pyridinium‐TAA derivatives and their cyclic voltammograms. a) Compounds **1–6**. b) Corresponding voltammograms recorded at 0.002 mol dm^−3^ in 0.1 mol dm^−3^ KCl. Scan rate 100 mV s^−1^.

The new pyridinium‐BMTAA derivatives were prepared via simple coupling reactions, either Ullmann‐type or Suzuki or a combination of both, followed by quaternization via the classic Menshutkin reaction (Schemes S2–S4 in Supporting Information). The chloride salts of the new derivatives were then characterized using cyclic voltammetry in aqueous media at a concentration of 0.002 mol dm^−3^ (0.1 mol dm^−3^ KCl as supporting electrolyte). As depicted in Figure 1, in the pyridinium‐BMTAA derivatives **1**,**2** ^–^ **4**,**5** the BMTAA/BMTAA^+^ moiety displayed an overall reversible behavior with positive formal potentials near ≈ +0.60 V versus Ag/AgCl, while the pyridinium moiety reduction was always found to be irreversible at peak potentials of ≈ −1.2 V versus Ag/AgCl with the only exception being **4** with a more negative reduction potential (≈ −1.3 V versus Ag/AgCl). The irreversibility of these peaks is consistent with an electrochemical reaction followed by a homogeneous chemical step involving an electrochemically inactive product.^[^ ^48^ ^]^ A different formal potential of + 0.87 V versus Ag/AgCl for the BMTAA/BMTAA^+^ moiety was obtained for **3**, featuring only one methoxy, protected by two *t*‐butyl groups, as suggested by Yang et al.,^[^ ^49^ ^]^ and two pyridinium moieties. In the latter case, the formal potential of the moiety was shifted to a higher value, due to the presence of only one methoxy group. In addition, a small peak was found at a peak potential of ≈ +0.5 V versus Ag/AgCl. Here, the addition of the *t*‐butyl moieties next to the only methoxy group did not lead to any improvement of the voltammetric behavior. All the modifications carried out on the pyridinium moiety did not change the observed irreversible behavior, whether a different connectivity on and to the aromatic linker to screen for possible electronic effects, as in **1–2** and **4–5**, or a switch to a more hydrophilic substituent on the nitrogen as in **5** to avoid a decrement in the solubility of the reduced species. A special case in the screened connectivity between the pyridinium unit and the posolyte moiety is represented by compound **6,** which resembles a 4‐aminopyridine derivative. Compound **6** features a pyridinium unit linked directly to a bis(4‐methoxyphenyl)amine unit (BMPA) via the amine nitrogen, that is, there is no additional aromatic ring between the negolyte and the posolyte moieties. The nonquaternized parent compound and its voltammogram in MeCN were already reported by Lim et al.;^[^ ^50^ ^]^ however, to our knowledge, the pyridinium analogue and its electrochemical characterization were not yet known. The voltammogram reported by Lim et al. for the parent compound of **6** shows a reversible behavior with a formal potential of ≈ +1.1 V versus NHE.^[^ ^50^ ^]^ Unfortunately, the voltammogram of **6** shows a drastic change from the derivatives in which a linker is placed between the negolyte and the posolyte units (e.g., compounds **1**–**5**), with irreversible oxidation at a peak potential as high as ≈ +1.10 V versus Ag/AgCl and a cathodic peak at ≈ +0.1 V versus Ag/AgCl. Peaks similar were reported for the voltammogram of pyridine in an aqueous solution and were related to a 2D transition in a pyridine‐adsorbed layer on gold.^[^ ^51^ ^]^ Overall, the lack of a linker between the two electroactive units did not lead to any improvement in the voltammogram. On the contrary, it had a detrimental impact on electrochemical reversibility and peak height.

An additional observation can be made for compounds **1–4** when the potential is scanned through positive values (i.e., from 0 to + 1.0 V versus Ag/AgCl) after a scan in the negative region (i.e., from 0 to − 1.4 V versus Ag/AgCl). In other words, if the BMTAA moiety is oxidized after the reduction of the pyridinium unit, new peaks appear in the voltammograms (see Figure. S2). An explanation for this can be that the electron taken from the pyridinium unit upon reduction is quickly delocalized over the phenyl bridge to the TAA‐type nitrogen, changing the electrochemical behavior of the TAA moiety upon oxidation. In the case of compound **4**, the latter phenomenon is more pronounced. The additional peaks disappear upon successive scan cycles, underlying the complex behavior of these molecules (see Figure. S2). These observations are likely related to the pyridine being quaternized. Indeed, in addition to the previous example for **6**, the parent compound of **1** (i.e., with a nonquaternized pyridine) was also recently reported in an investigation on push‐pull systems in nonaqueous media by Tydlitát et al. and its voltammogram showed two reversible events, one for the pyridine and the other for the TAA unit in DMF.^[^ ^47^ ^]^

### 2.2 Synthesis and Cyclic Voltammetry of New Bipolar Bipyridinium‐BMTAA Derivatives

**Figure 2:**
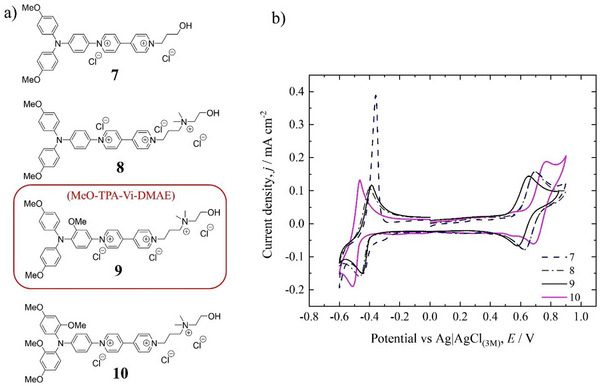
New bipyridinium‐TAA derivatives and their cyclic voltammograms a) Compounds **7–10**. b) Corresponding voltammograms recorded at 0.001 mol dm^−3^ in 0.1 mol dm^−3^ KCl. Scan rate 100 mV s^−1^.

Considering the unsatisfactory electrochemical reversibility obtained by tethering a pyridinium to the BMTAA moiety, the water‐solubilizing negolyte moiety was changed to a 4,4´‐bipyridinium derivative, that is, a viologen. When screening simple procedures to connect a viologen to the chosen BMTAA unit, the Zincke reaction appeared straightforward. The latter is a reaction that allows the transformation of a primary amine into its *N*‐alkyl or *N*‐aryl pyridinium salt by the use of the so‐called Zincke salt N‐2,4‐dinitrophenyl)‐pyridinium chloride.^[^ ^52^ ^]^ To perform this reaction, the BMTAA moiety was chosen as the primary amine fragment, while the Zincke salt was prepared from the 4,4´‐bipyridine with one of the two rings functionalized with a hydrophilic substituent (Schemes S6,S7 from Supporting Information). According to recent reports, the presence of hydroxyl and ammonium groups was shown to increase the hydrophilicity of the viologen, in addition to a possible stabilization from the alcohol residues on the radical cation.^[^ ^18^, ^53^, ^54^ ^]^ Thus, a propanol residue and a recent dimethylaminoethanol‐based compound were chosen.^[^ ^18^ ^]^ The new BROMs, as shown in Figure. 2a, were prepared, and their chloride salts were characterized electrochemically. As shown in Figure. 2b, the first reduction of the viologen side in compound **7** (bearing a propanol residue) has a peak potential of ≈ −0.42 V versus Ag/AgCl and on the reoxidation shows the typical “spike” behavior which signals irreversibility as reported for the second reduction of MV in water. In the latter case, the “spike” is associated with deposition and stripping at the working electrode due to the insolubility of MV^0^.^[^ ^55^ ^]^ Here, the lower solubility of **7** ^+*^ compared to the pristine cationic state (i.e., **7** ^2+^) might be responsible for the observed irreversibility of the first reduction.

Regarding the oxidation of the posolyte moiety of **7**, a reversible behavior can be seen at a formal potential of ≈ +0.66 V versus Ag/AgCl for the TAA/TAA^+^ moiety, but as shown in Figure 2b, a small additional peak at ≈ +0.30 V versus Ag/AgCl precedes it. The latter is speculated to be due to the presence of a dimer that is oxidized at a lower potential,^[^ ^55^ ^]^ or to the formation of a carbazole upon radical delocalization^[^ ^55^ ^]^ or to the oxidation of decomposition product(s) derived from processes in the bulk of the solution.^[^ ^31^ ^]^ Compound **8**, featuring a more hydrophilic substituent, shows a reversible first reduction for the viologen, namely for Vi^3+^/Vi^2+^, at a formal potential of ≈ −0.42 V versus Ag/AgCl, while the second reduction remains irreversible at a peak potential of ≈ −0.70 V versus Ag/AgCl as seen in Figure. S3. The behavior of the TAA moiety in **8** resembles that of **7**, having a positive formal potential of ≈ +0.66 V versus Ag/AgCl for the TAA/TAA^+^ couple, where the latter is preceded by a small additional peak at ≈ + 0.30 V versus Ag/AgCl. The latter becomes less evident on a second scan (see Figure. S4), thus suggesting the presence of processes taking place in the solution and the possible formation of an insoluble film at the electrode surface.^[^ ^31^ ^]^

**Figure 3:**
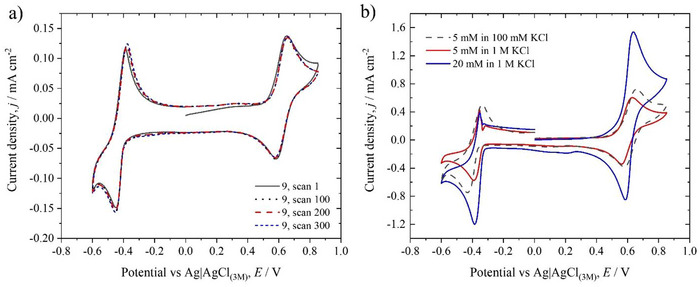
Additional voltammograms for **MeO‐TAA‐Vi‐DMAE (9)**. a) Selected scans recorded at 0.001 mol dm^−3^ in 0.1 mol dm^−3^ KCl; b) Effect of BROM concentration and supporting electrolyte. Scan rate 100 mV s^−1^.

Encouraged by the previous results, two additional modifications on the BMTAA moiety were evaluated to expand the scope on the posolyte side and assess their effect on the electrochemistry of the BROMs, respectively. In particular, the first modification involved an additional methoxy group on the central aromatic linker in a strategic position as seen in compound **9,** hereafter referred to as **MeO‐TAA‐Vi‐DMAE**. The presence of a methoxy group in *ortho* to the BMPA moiety was speculated to be beneficial to block a possible reactive site upon oxidation, for example, to prevent the carbazole formation as reported by Blanchard et al.^[^ ^55^ ^]^ and to further twist the dihedral angle between the three rings around the central nitrogen atom. The second modification also focused on the dihedral angle between the three rings and the blocking of possible reactive sites by the modification of the BMPA moiety; this was accomplished by adding two more methoxy groups (one per ring), but leaving the aromatic linker unchanged, via the use of 2,4‐dimethoxyiodobenzene instead of 4‐iodoanisole in the Ullmann condensation thus leading to **10** (Scheme S5 from Supporting Information). The dihedral angle between the three rings along the central nitrogen is expected to have a significant effect on the electronic features of the molecule, and in turn, its electrochemical behavior, as also found by Kim et al.^[^ ^56^ ^]^ The corresponding diamines were prepared in just two steps as described in the Supporting Information (pp 29–31) and used as a substrate for the Zincke reaction. The chloride salt of **MeO‐TAA‐Vi‐DMAE** (**9**) displays a reversible TAA/TAA^+^ unit with a formal potential of ≈ +0.62 V versus Ag/AgCl and a reversible viologen unit, namely Vi^3+^/Vi^2+^, with a formal potential of ≈ ^−^0.42 V versus Ag/AgCl, as seen in Figure. 2. The presence of the additional methoxy group did not alter the redox potentials of **MeO‐TAA‐Vi‐DMAE** (**9**) when compared to **7** or **8** but it affected the additional peak that appeared for **7** and **8** at a peak potential of ≈ +0.30 V versus Ag/AgCl. Indeed, the peak appears to be missing for **MeO‐TAA‐Vi‐DMAE** (**9**) (see Figure S5). This result might be explained by the change in the dihedral angles between the planes of phenyl rings induced by the additional methoxy in a strategic position when compared to the pristine derivatives, that is, with no additional substituents on the aromatic linker. This would be consistent with findings from Kim et al. on the effect of the number and position of methoxy groups in TAA donors.^[^ ^56^ ^]^ The chloride salt of **10** shows reversibility for the TAA/TAA^+^ couple, with a formal potential ≈ +0.73 V versus Ag/AgCl, which is slightly higher than the value for **MeO‐TAA‐Vi‐DMAE (9),** but it is preceded by a small additional shoulder at a potential ≈ +0.55 V versus Ag/AgCl which is likely related to the presence of additional methoxy groups. The latter finding is interesting when compared to the voltammograms reported by Kim et al. for new photosensitizers based on TAA moieties,^[^ ^56^ ^]^ where a similar phenomenon was observed when two methoxy groups were in the *ortho* position to each other. The slight anodic shift to higher values was unexpected due to the presence of additional EDGs, which theoretically should have increased the electron density and lowered the oxidation potential. Regarding the viologen moiety, **10** shows a reversible Vi^3+^/Vi^2+^ couple with formal potential ≈ −0.50 V versus Ag/AgCl, which is slightly more negative than the values for **7**, **8**, and **MeO‐TAA‐Vi‐DMAE** (**9**). However, it should be underlined that also for **MeO‐TAA‐Vi‐DMAE** (**9**) and **10** the second reduction remained irreversible (see Figure S3), likely due to a much lower solubility of the double reduced species and possible side reactions such as dimerization or adsorption at the electrode. Based on the preliminary voltammograms, **MeO‐TAA‐Vi‐DMAE** (**9**) was selected for further evaluation. The voltammetry also suggests a reasonable stability for this compound, as its shape did not change over 300 oxidation‐reduction cycles for the 0.001 mol dm^−3^ solution, as shown in Figure  3a, while in the case of **10**, the voltammograms started to change on the 100^th^ scan (see Figure. S6).

However, while the presence of methoxy groups is beneficial for some aspects, as highlighted by the results presented here and previous works,^[^ ^30^ ^]^ it can also represent the molecule's Achilles heel. As reported by Zhou et al.,^[^ ^49^ ^]^ if a TAA derivative containing a methoxy group is subjected to a sufficiently high potential, the latter undergoes oxidative demethylation and the overall TAA derivative loses reversibility. This finding is also confirmed in this work (see Figure S7a,b). The lack of a clear second oxidation peak for **MeO‐TAA‐Vi‐DMAE** (S7b) in comparison to **10 (** S7a) is likely due to a fortuitous overlap with the oxygen evolution reaction (OER). However, the appearance of increased redox activity between − 0.4 V and 0.4 V versus Ag/AgCl is consistent with the aforementioned oxidative demethylation. The methoxy groups could also be deprotected to yield the free OH groups as proposed by Wang et al. for their 4,4′,4″‐trihydroxytriphenylamine.^[^ ^30^ ^]^ However, a weakly acidic electrolyte is needed for this strategy, in contrast with the pH‐neutral KCl‐based electrolyte here chosen.

By continuing the electrochemical characterization of **MeO‐TAA‐Vi‐DMAE** (**9**), the effect of the supporting electrolyte concentration on the voltammograms was assessed at two different concentrations of redox‐active species, as shown in Figure 3b. Increasing the concentration of the active species to 0.005 mol dm^−3^ in the 0.1 mol dm^−3^ KCl solution did not have any detrimental effect, that is, did not change the peak heights or peak potentials significantly. However, when the concentration of the supporting electrolyte was increased up to 1 mol dm^−3^ KCl, to match industrially relevant conditions, the behavior of **MeO‐TAA‐Vi‐DMAE** (**9**) changed dramatically. As seen in Figure 3b, the viologen's first reduction became irreversible at 0.005 and at 0.02 mol dm^−3^ in 1 mol dm^−3^ KCl, probably due to a homogeneous chemical reaction of the product following the electrochemical step, likely involving a drastic decrease in the solubility of the reduced species and possibly related to polymerization. A similar shape in the voltammograms was indeed observed by Martinez‐González et al. when screening the concentration and counter anion effects on commonly used FB electrolytes such as viologen derivatives.^[^ ^57^ ^]^

**Figure 4:**
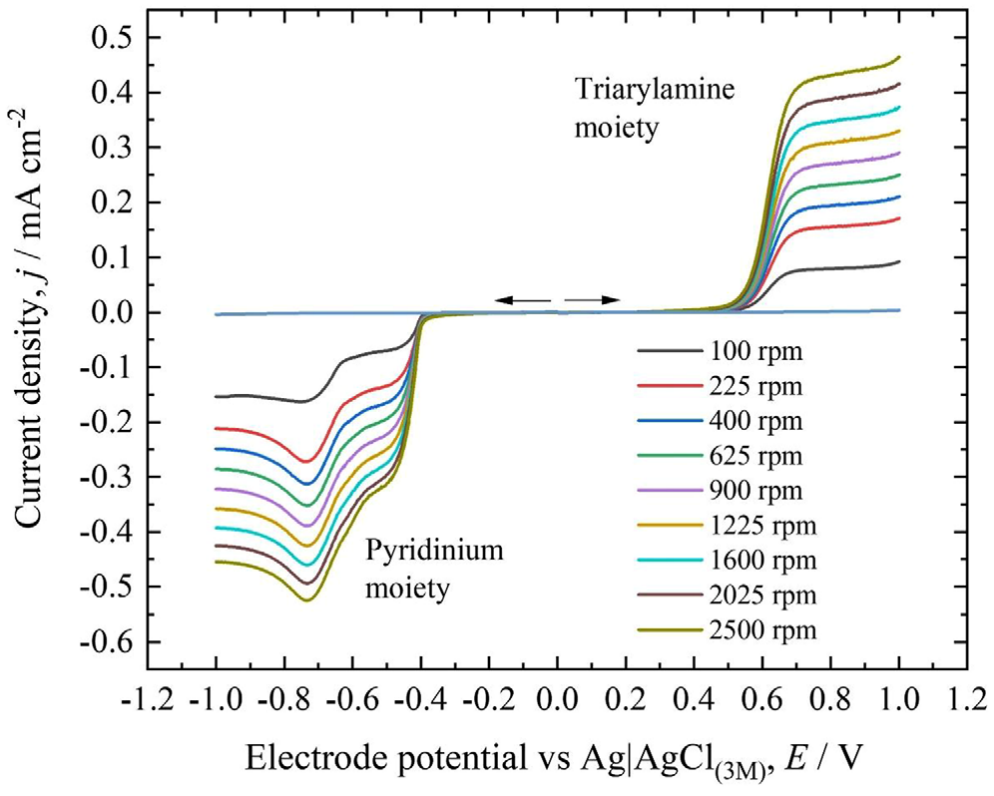
Hydrodynamic linear sweep voltammetry for compound **MeO‐TAA‐Vi‐DMAE** (**9**) at glassy carbon RDE. Concentration 0.001 mol dm^−3^ in 0.1 mol dm^−3^ KCl. Scan rate 5 mV s^−1^. The background current for the supporting electrolyte is also shown.

Further confirmation of this hypothesis derives from the RDE experiments upon reaching the second viologen reduction at low potentials, as seen in Figure 4. The LSV shows a strongly potential‐dependent cathodic current for the viologen moiety. Plus, an unexpected peak appears in what would ideally be the limiting current region, with peak potential at ≈ −0.73 V versus Ag/AgCl, nearly independent of rotation rate. The existence of this peak under forced convection suggests a reaction on a deposited layer adjacent to the electrode surface as reported for RDE electrodes coated with insoluble polymerized films of nanometric thickness, where it could be associated to a obstructed diffusion across the assumed thin film.^[^ ^58^, ^59^ ^]^ This is consistent with the cathodic deposition of insoluble MV films reported by Engelman et al.^[^ ^60^ ^]^ The peak could be catalyzed ORR despite the supply of inert gas, with oxygen possibly coming from the counterelectrode in the undivided cell. Nevertheless, we are inclined to consider this unlikely in view of negligible oxygen routinely achieved in appropriate cells fitted with inert gas supply at such currents and timescales.^[^ ^18^ ^]^

Additionally, it is likely that upon increasing the concentration of the supporting electrolyte, the high ionic strength of the solution further exacerbates the lower solubility of the reduced species, making even the first reduction irreversible. Indeed, the solubility of the pristine cationic state in water was estimated to be 0.8 mol dm^−3^ (± 0.35) in water (see UV‐Vis section in Supporting Information p. 2 and Figures S8,S9). This value is lower than the solubility of the few known examples of BROMs. Indeed, Liang et al. reported a solubility of 1.76 mol dm^−3^ for (TAABPy)Cl_3_, Luo et al. a value of 1.80 mol dm^−3^ for Zn[Fc(SPr)_2_], and Zhu et al. 1.2 mol dm^−3^ for Fc‐bpy^3+^. Taking everything into account, namely the loss of reversibility for **MeO‐TAA‐Vi‐DMAE** (**9**) at increased concentration of supporting electrolyte, the solubility is recognized as the bottleneck for further characterization, including flow cells under relevant conditions (i.e., concentration in the molar range) of this new class of BROMs. As a possible strategy to solve this issue and increase the water solubility, the switch to 2‐methoxyethanol residues instead of simple methoxy groups is speculated to be effective. Indeed, 2‐methoxyethanol residues could increase the overall hydrophilicity of the molecule in all the redox states and, in turn, improve its electrochemical properties in high ionic strength environments. Regarding the oxidative demethylation decomposition pathway, we speculate that the use of 2‐methoxyethanol residues could be beneficial. As an alternative, other electron‐donating stabilizing groups not based on oxygen can also be screened.

### 2.3 Challenges in Hydrodynamic Voltammetry for BROMs

Following the promising cyclic voltammograms of **MeO‐TAA‐Vi‐DMAE** (**9**) under diluted conditions, hydrodynamic voltammetry was performed using the RDE technique to assess its diffusion coefficient and, if possible, to obtain kinetic data. Since the newly developed ROMs are bipolar and the supporting electrolyte is potassium chloride, where K^+^ and Cl^−^ have extremely similar effective ionic radii (and thus ion mobility), it can be considered that both the positive and the negatively charged redox state of the electroactive substance have very similar effective radii and thus similar diffusion coefficients. Therefore, the diffusion coefficient may be estimated from either the anodic or the cathodic limiting currents using the Levich equation, which quantitatively relates the inverse limiting currents with the inverse of the angular velocity of the working electrode. However, since the reduction reaction of the Vi^3+/^Vi^2+^ couple was already found to be most likely coupled to a strongly hindered, film‐dependent diffusion process (Figure 4), the anodic limiting current for the TAA/TAA^+^ couple was favored instead at first glance.

**Figure 5:**
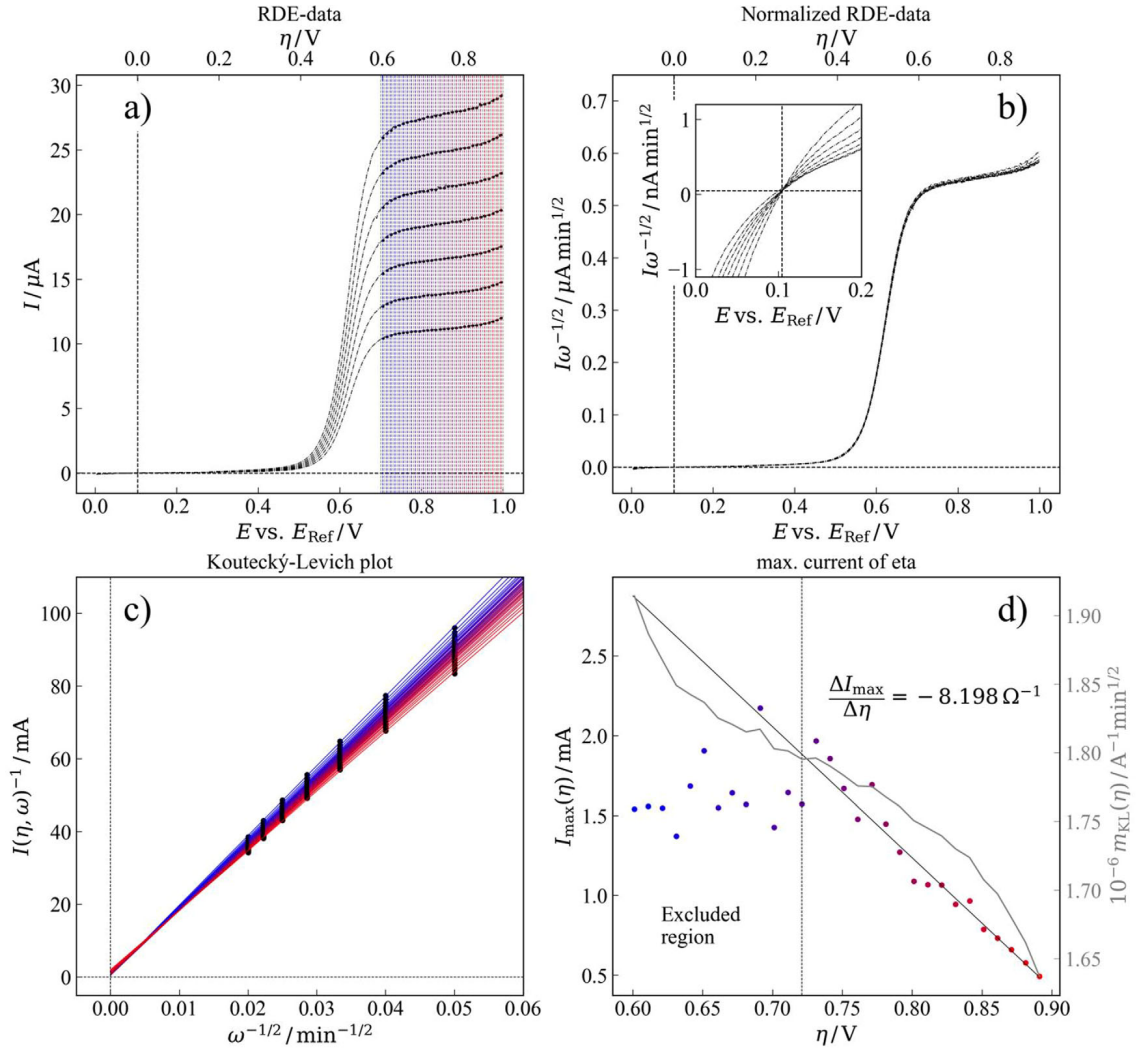
Extended Koutecký–Levich analysis of the oxidation of the TAA moiety of **MeO‐TAA‐Vi‐DMAE** (**9**). a) Linear sweep voltammetry at glassy carbon RDE (400 to 2500 rpm); b) Normalized RDE data showing a fast electrode reaction under full diffusion control; c) Koutecký–Levich plots purposely constructed from points in the mass transfer dominated region; d) Maximum current and Levich slopes versus overpotential.

Visual inspection of the hydrodynamic voltammetry data depicted in Figure 5a reveals, however, that it is a nonstraightforward task to find the actual anodic limiting current since no well‐defined current plateau is formed. In contrast, the current appears to be dependent on the potential, yet with a rather linear increase (somewhat similar to an Ohmic resistance) in the region where an ideal limiting value would be expected. This particular slope was the main motivation for the theoretical considerations elucidated below. It should be noted that all the experimental results were compensated for Ohmic drop and that this behavior is unrelated to *IR* losses in the three‐electrode cell (see Figure S10).

As mentioned above, revision of the literature shows that potential‐dependent currents at the mass transfer dominated hydrodynamic conditions can be found in RDEs coated with thin films, for example, Refs.[61, 62, 63] Such films are less than 50 nm thick, and several modified Koutecký–Levich relations have been considered to model the response of such “film‐coated RDEs” based on competing mass transfer rates.^[^ ^64^, ^65^ ^]^ However, since we could not find previous theoretical explanations for the nonideal limiting currents (i.e., their “slope”) in the mass transfer dominated region and because the electrodes used in our experimental setup were not coated a priori, we first focused on two different hypotheses.

From our previous work,^[^ ^38^ ^]^ it is known that finite electron transfer kinetics can lead to a mismatch in the theoretically expected hydrodynamic limiting currents (obtained from a back‐calculation of the slope obtained from a Koutecký–Levich analysis) and the experimentally observed current plateaus of a RDE measurement. However, it should be strongly emphasized that in these scenarios, a current plateau, and thus an implied limiting current, is still formed. Since this is particularly not the case for the RDE data of **MeO‐TAA‐Vi‐DMAE** (**9**), where no current plateau is ever reached, a different explanation is required.

In this context, one alternative would be a replacement of the finite electron transfer rate by an Ohmic, thus potential‐dependent, limiting term. In this manner, one could readily address the “slope” of the potential‐dependent hydrodynamic current in question. A second idea, which could also explain the poorly defined current plateau and the increasing “slope” at higher overpotentials, would be the consideration of another electrochemical oxidation reaction starting at larger overpotentials and contributing to the overall current. For now, it is not necessary to specify if this secondary reaction is the degradation of the BROM (such as the aforementioned oxidative demethylation of the TAA moiety) or OER.

The scope of the following theoretical considerations is to clarify which of the two aforementioned effects is more reasonable for the BROM, **MeO‐TAA‐Vi‐DMAE** (**9**), used in this study, as this will contribute to the understanding of its particular electrochemical reactions.

### 2.4 Introduction of an Ohmic Term into the Koutecký–Levich Equation

Considering the generalized current‐overpotential relation for a one‐step‐one‐electron transfer electrode reaction with Butler‐Volmer kinetics under forced convection, the Faradaic current of an RDE experiment can be expressed by:

(1) $$I_{\mathrm{far}}\;\left(\xi ,\omega \right)=\frac{\left(e^{\alpha \xi }-e^{-\left(1-\alpha \right)\xi }\right)}{\frac{1}{I_{\mathrm{eq}}\;}+\frac{e^{\alpha \xi }}{I_{\lim,\mathrm{an}}\left(\omega \right)}-\frac{e^{-\left(1-\alpha \right)\xi }}{I_{\lim,\mathrm{ca}}\left(\omega \right)}}\;,$$

where ξ is the dimensionless overpotential according to

(2) $$\xi =\frac{nF}{RT}\;\left(E-E_{\mathrm{eq}}\right),\;$$

where E and $E_{\mathrm{eq}}$ stand for the electrode potential and the equilibrium electrode potential, respectively, n is the number of transferred electrons per redox‐step and *F*, *R*, and *T* have their usual meaning as Faraday's constant, gas constant, and absolute temperature. Additionally, $I_{\mathrm{eq}}$ is the exchange‐current defined by

(3)

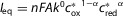

 and $I_{\lim,\mathrm{an}}$ and $I_{\lim,\mathrm{ca}}$ are the anodic and cathodic limiting currents defined by Equations (4) and (5), respectively.

(4) $$I_{\lim,\mathrm{an}}\;\left(\omega \right)=0.201nFAc_{\mathrm{red}}^{*}D_{\mathrm{red}}^{2/3}\mu^{-1/6}\omega^{1/2}\;$$

(5) $$I_{\lim,\mathrm{ca}}\;\left(\omega \right)=-0.201nFAc_{\mathrm{ox}}^{*}D_{\mathrm{ox}}^{2/3}\mu^{-1/6}\omega^{1/2}\;$$

In Equations (1) to (6) it is, A the geometrical area of the working electrode, $k^{0}$ the standard heterogeneous rate constant, α the electron transfer coefficient, $D_{\mathrm{ox}}$, and $D_{\mathrm{red}}$ the Fickian diffusion coefficients and $c_{\mathrm{ox}}^{*}$ and $c_{\mathrm{red}}^{*}$ the bulk concentrations of oxidized and reduced species, respectively. The parameter μ is the kinematic viscosity of the supporting electrolyte, ω the angular velocity of the rotating‐disc working electrode in revolutions per minute (rpm).

By defining the kinetic current (in absence of any mass transfer limitations) of the anodic reaction as

(6) $$I_{\mathrm{kin},\;\mathrm{an}}\;\left(\xi \right)=I_{\mathrm{eq}}\;e^{\alpha \xi }$$

and its cathodic analogue according to

(7) $$I_{\mathrm{kin},\;\mathrm{ca}}\;\left(\xi \right)=I_{\mathrm{eq}}\;e^{-\left(1-\alpha \right)\xi },\;$$

Equation (1) takes the form

(8) $$I_{\mathrm{far}}\;\left(\xi ,\omega \right)=\frac{I_{\mathrm{kin},\mathrm{an}}\left(\xi \right)-I_{\mathrm{kin},\;\mathrm{ca}}\;\left(\xi \right)}{1+\frac{I_{\mathrm{kin},\mathrm{an}}\left(\xi \right)}{I_{\lim,\mathrm{an}}\left(\omega \right)}-\frac{I_{\mathrm{kin},\mathrm{ca}}\left(\xi \right)}{I_{\lim,\mathrm{ca}}\left(\omega \right)}}$$

Considering that $I_{\mathrm{far}}\left(\xi \right)$ is the potential‐dependent Faradaic current of the reaction under investigation, the Faradaic DC‐resistance (not to be confused with the Faradaic impedance) may be defined by

(9) $$R_{\mathrm{far}}\;\left(\xi \right)=\frac{E-E_{\mathrm{eq}}}{I\left(\xi \right)}$$

Now, considering that the Faradaic DC‐resistance is connected in series to a resistance $R_{\mathrm{ser}}$, the total DC‐resistance of the system $R_{\mathrm{tot}}\left(\xi \right)$ will be given by

(10) $$R_{\mathrm{tot}}\;\left(\xi \right)=R_{\mathrm{far}}\left(\xi ,\omega \right)+R_{\mathrm{ser}}=\frac{E-E_{\mathrm{eq}}}{I\left(\xi ,\omega \right)}+R_{\mathrm{ser}}.\;$$

Substituting $R_{\mathrm{tot}}$ by its Ohmic representation yields the reciprocal total current $I_{\mathrm{tot}}\left(\xi ,\omega \right)$ at a given overpotential according to

(11) $$\;\frac{1}{I_{\mathrm{tot}}\left(\xi ,\omega \right)}=\frac{1}{I\left(\xi ,\omega \right)}+\frac{R_{\mathrm{ser}}}{E-E_{\mathrm{eq}}}=\frac{1}{I\left(\xi ,\omega \right)}+\frac{1}{I_{\mathrm{ser}}\left(\xi \right)}.\;$$

Using Equation (8) on Equation (11) finally yields

(12) $$\begin{array}{ccc} \; & & \frac{1}{I_{\mathrm{tot}}\left(\xi ,\omega \right)}=\frac{1}{I_{\mathrm{kin},\mathrm{an}}\left(\xi \right)-I_{\mathrm{kin},\;\mathrm{ca}}\left(\xi \right)}\;\\ & & \;\left(1+\frac{I_{\mathrm{kin},\mathrm{an}}\left(\xi \right)}{I_{\lim,\mathrm{an}}\left(\omega \right)}-\frac{I_{\mathrm{kin},\mathrm{ca}}\left(\xi \right)}{I_{\lim,\mathrm{ca}}\left(\omega \right)}\right)+\frac{1}{I_{\mathrm{ser}}\left(\xi \right)}.\; \end{array}$$

From Equation (12), it is now readily seen that either $I_{\mathrm{kin},\mathrm{an}}\left(\xi \right)$ or $I_{\mathrm{kin},\mathrm{ca}}\left(\xi \right)$ will govern the denominator at sufficiently large positive or negative overpotentials.

Thus, for an anodic reaction with $|I_{\mathrm{kin},\mathrm{an}}\left(\xi \right)\left|\gg \right|I_{\mathrm{kin},\mathrm{ca}}\left(\xi \right)|$, Equation (12) can be vastly simplified. This gives

(13) $$\;\frac{1}{I_{\mathrm{tot}}\left(\xi \right)}=\frac{1}{I_{\mathrm{kin},\mathrm{an}}\left(\xi \right)}+\frac{1}{I_{\lim,\mathrm{an}}\left(\omega \right)}+\frac{1}{I_{\mathrm{ser}}\left(\xi \right)},\;$$

Which is nothing but a modified Koutecký–Levich equation containing an Ohmic term.

Considering that $I_{\mathrm{kin},\mathrm{an}}\left(\xi \right)\gg I_{\lim,\mathrm{an}}$ at the mass transfer dominated region (i.e., *“limiting current conditions”*), Equation (13) reduces to

(14) $$\;\frac{1}{I_{\mathrm{tot}}\left(\xi ,\omega \right)}=\frac{1}{0.201nFAc_{\mathrm{red}}^{*}D_{\mathrm{red}}^{2/3}\mu^{-1/6}}\;\frac{1}{\omega^{1/2}}+\frac{1}{I_{\mathrm{ser}}\left(\xi \right)},\;$$

where the anodic limiting current was replaced by its definition given in Equation (4). Considering that an electrochemical system would follow Equation (14), it is now readily seen that a classical Koutecký–Levich analysis, of the inverse *limiting current* versus the inverse rotation speed of the electrode will inevitably lead to a potential‐dependent offset. It is exactly this quantity which is the subject of the following analysis.

### 2.5 Three‐Term Koutecký–Levich Analysis of the Oxidation of the TAA Moiety of **Meo‐TAA‐Vi‐DMAE** (**9**)

For analyzing the RDE data, in the context of Equation (14), the first challenge was to find the equilibrium potential. In this regard, it was found that all LSV curves of the oxidation of the TAA moiety of **MeO‐TAA‐Vi‐DMAE** (**9**) (depicted in Figure 5a), normalized by the square root of the rotation speed of the working electrode merge in one particular point (cf. Figure 5b, inset) which is very close to zero current. It is assumed that this particular point at + 0.1 V versus Ag/AgCl corresponds to the equilibrium potential and that the small nonzero current results from the capacitive charging of the electrochemical double‐layer in the direction of the potential sweep in the positive direction (for an anodic reaction). For this reason, a secondary abscissa in terms of overpotential (instead of potential versus the reference electrode) has been included in Figure 5a, b.

Since Equation (14) suggests a potential‐dependent ordinate intersect in a Koutecký–Levich‐like analysis and no well‐defined (yet potential independent) limiting current is achieved, a two‐step analysis is introduced. In the first step, a potential‐resolved Koutecký–Levich analysis has been performed. For this purpose, the region, where a limiting current would be expected, has been subdivided into steps of 50 mV each.* Starting from an overpotential of + 0.6 V and up to an overpotential of + 0.9 V, the potential dependent current limits have been computed by the local average of each of the 50 mV intervals and assigned to the respective midpoint‐potentials which are indicated by the vertical lines, grading from blue (low overpotentials) to red (larger overpotential) in Figure 5a. The corresponding Koutecký–Levich lines are shown in Figure 5c with the same color code. It can be seen that all curves possess a very low, yet potential‐dependent, ordinate intercept. Additionally, there is a change in the slope of the Levich lines at different overpotentials.

This change in slope is particularly not expected in the framework of Equation (14), which only predicts a potential‐dependent offset. For this reason, both effects, that is, the potential‐dependent offset and the potential‐dependent slope are investigated further in the analysis shown in Figure 5d. This subplot depicts the inverse of the potential‐dependent ordinate intercepts of Figure 5c on the primary ordinate and the potential‐dependent slopes of the Levich lines on the secondary ordinate versus the actual overpotentials on the abscissa.

It can be seen that the inverse of the potential‐dependent ordinate intercepts (thus $I_{\mathrm{ser}}\left(\xi \right)$ from Equation (13)) fall on a straight line at overpotential larger than 0.72 V. Below an overpotential of 0.72 V, however, no linear relation between the inverse ordinate intercepts and the overpotential is found and data is scattered. Therefore, performing a linear regression was restricted to overpotentials larger than 0.72 V (right side of the dashed line in Figure 5d). This leads to an apparent resistance value of $R_{\mathrm{ser}}$ ≈ ^−^0.12 Ω (i.e., $\Delta I_{\max}/\Delta \eta$ = ^−^8.2 Ω^−1^) which is counterintuitive at a first glance due to its sign.

Regarding Figure 5d, the negative resistance contribution is, however, not the only feature that appears somewhat odd. Considering the slope of the Levich lines with the overpotential (depicted on secondary ordinate and the curve colored in grey), an s‐shaped response with an inflection point at around 0.72 V is obtained. While one could speculate that this inflection point may be the ideal choice for estimating the hydrodynamic parameters of the BROM under investigation, a more decent approach would be to first reproduce the experimentally observed behavior qualitatively with a theoretical model before phrasing such claims. This is exactly what is shown in the ensuing subsection.

### 2.6 Modeling the Potential‐Dependent Slope Transient in a Koutecký–Levich Plot—Ohmic Resistance Versus Second Reaction

For the following theoretical considerations, Equation 14 is mainly used. Approximate values for the hydrodynamic limiting currents are deduced from the Koutecký–Levich slope at the inflection point of 0.72 V in Figure 5d, so 1.8 × 10^−6^ A^−1^ min^1/2^. Other parameters were set by educated guesses, which are elaborated below.

**Figure 6:**
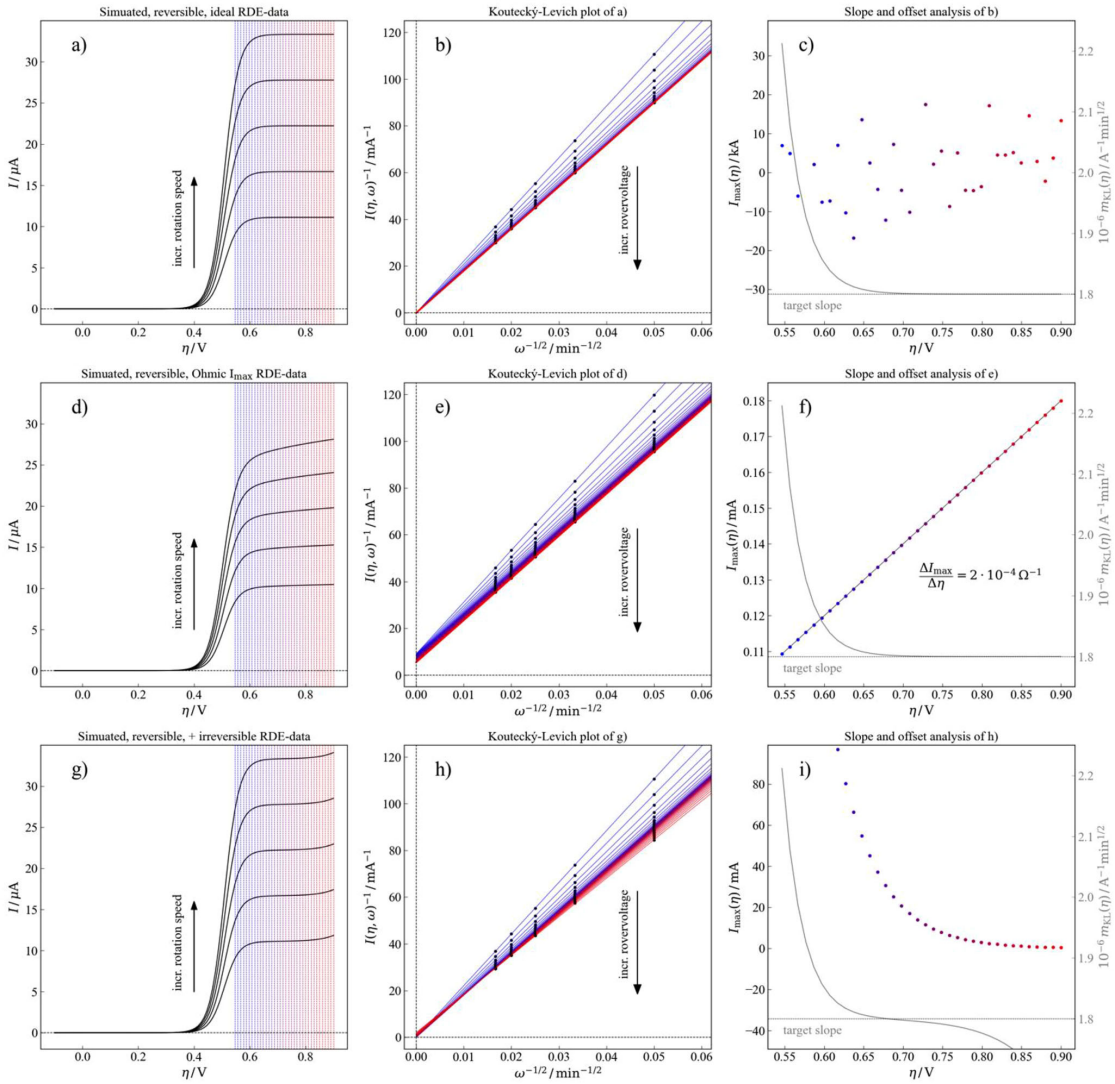
Simulations for a) a reversible electron transfer reaction, b) a reversible electron transfer reaction under a transfer resistance of 5000 Ohm and c) a reversible electron transfer reaction + another, kinetically controlled reaction and the corresponding (from left to right) potential‐dependent Koutecký–Levich analyses b), e), h), and slope + offset analyses c), f), i).

Carefully looking at the ascending part of the linear voltage sweeps in Figure 5a, it can be expected that the actual electron transfer reaction is rather fast, that is, the process is reversible from an electrochemical point of view. Electrochemical reversibility may be considered by setting $I_{\mathrm{eq}}$ to an exotically large value of 1 kA. Under these circumstances, the electron transfer kinetics are about nine orders of magnitude faster than the hydrodynamic limitation, thus ensuring they are not the bottleneck of the reaction. The linear sweep voltammograms, computed for this reversible scenario, are depicted in Figure 6a.† It can be seen that, unlike the experimental data in Figure 5a, a true limiting current is formed. This quantity is in the range of the experimental data, that is, about 30 µA for the fastest rotation speed.

The potential‐dependent Koutecký–Levich analysis on this synthetic dataset is depicted in Figure 6b and reveals three key insights:

- It can be seen that a potential‐dependent Levich slope is formed, which converges to a certain value with increasing overpotentials. This means that nonconvergent slopes are observed only in the region of potential where the linear‐sweep‐voltammograms are curved. This is only in partial agreement with the experimental findings, where a potential‐dependent slope of the Levich lines is seen.
- All Levich lines reach the origin and applying the slope and offset analyses of Figure 5 to the results of Figure 6b results in Figure 6c. It can be seen that this particular offset analysis (aiming for the hypothetical current limit at infinite rotation speed) only reveals a set of vastly scattered points in the kiloampere range. Thus, no trend (or even a linear relation) is seen. This is somewhat intuitive, since a reversible (exceptionally fast) reaction was assumed. Therefore, the scattering is mainly caused by numerical artifacts of the simulation.
- The most interesting result is obtained from the slope analysis. There, it can be seen that at overpotentials larger than 0.7 V the initially assumed Levich slope of 1.8 × 10^−6^ A^−1^ min^1/2^ is recovered. This is in excellent agreement with the experimental results and supports the assumption that the actual electron transfer reaction can be considered reversible. However, since no “slope” is seen in Figure 6a, this “only reversible reaction” model cannot resemble the experiment sufficiently well. Plus, no onset of secondary reaction in the high‐overpotential region of the linear sweep voltammograms is seen in the simulation.

Since the assumption of an ideally reversible reaction does not explain the experimental findings, an additional simulation, including the third term in the Koutecký–Levich equation according to Equation 14, has been performed. Respective linear‐sweep‐voltammograms are depicted in Figure 6d. It can be seen that in this case, a potential‐dependent slope in the limiting currents is introduced, which increases as the virtual angular velocity of the electrode increases. This is in qualitative agreement with the experimental data. However, a resistance of $R_{\mathrm{ser}}$ ≈ 5000 Ω is required to get this qualitative match. Disregarding the sign of the experimentally observed value of $R_{\mathrm{ser}}$ ≈ ^−^0.12 Ω, this deviates by almost four orders of magnitude and is thus considered unrealistic. Additionally, it can be seen that the limiting current for the largest virtual rotation speed of the electrode is only about 25 µA (cf. Figure 6d). This is less than the experimentally obtained value (cf. Figure 5a) and also less accurate when compared to the “just reversible case” (cf. Fig. 6a). Nevertheless, the potential‐dependent Koutecký–Levich analysis has been performed. Its results are shown in Figure. 6e, f. Again, it can be seen that a potential‐dependent slope of the Levich lines is observed, which, unlike the experimental data of Figure. 5d, converges if the overpotentials (cf. Figure 6e, f, grey curve) surpass 0.7 V. The respective offset analysis (cf. Figure 6f) reveals a straight line with a positive slope. From this slope, the oddly large resistance of $R_{\mathrm{ser}}$ ≈ 5000 Ω can be, as expected, recovered easily.

These results imply that an exceptionally large resistance would be required for distorting the linear sweep voltammograms in the observed manner. Thus, the “reversible reaction + resistance distortion” hypothesis is considered unrealistic as well for our case. However, this could be qualitatively consistent with the resistive behavior observed in poorly performed experiments, in particular when the RDE —or another cell component— has faulty electrical connections. It is also possible that the high resistance resides in the film itself,^[^ ^66^ ^]^ for which appropriate models and measurements could be developed.

Yet another approach was introduced, which assumes a second oxidation reaction (not specifying which redox species is consumed) starting at large overpotentials. The results of this simulation are depicted in Figure 6g. It can be seen that this “two reaction hypothesis” is so far the only circumstance that qualitatively reproduces the current magnitude of the experimental data (compared to Figure 5a) and the current increment over 0.9 V, which seems kinetically affected (see Figure 5b), and which could be the onset of the second oxidation of **MeO‐TAA‐Vi‐DMAE** (**9**) or simply the OER on glassy carbon (its peak at + 1.2 V versus Ag/AgCl in neutral conditions), especially when the redox species is at such low concentration (see Fig. S7 and comments therein). The respective potential‐dependent Koutecký–Levich analyses depicted in Figure 6h also reproduce the experimental data well, that is, a potential‐dependent slope is obtained, which does not converge at all and rather implies a region of convergence. The “slope analysis” is depicted in Figure 6i. It can be seen that now the very same s‐curve characteristic, as seen in the experimental data, is observed, which is considered a strong implication that the “two reaction hypothesis” is the most realistic model to explain the experimental findings. More importantly, however, it can be seen that the predefined Levich slope of 1.8 × 10^−6^ A^−1^ min^1/2^ is precisely recovered at the inflection point of this particular s‐curve. This means that the potential‐dependent Koutecký–Levich analysis presented in this manuscript can be used to accurately identify the actual hydrodynamic parameters of an electrochemical system, even if the experimental data does reveal only a poorly defined limiting current. However, it is worth noting that unlike the experiment, the offset analysis did not reveal a linear trend. However, still, the “negative trend” in the resistance was reproduced to some extent. This feature can be explained by the second reaction, which opens another branch for the current to flow and thus results in a certain amount of “extra current” at a given overpotential and thus a negative resistance.

It is therefore assumed that this particular potential is the optimal choice for estimating the diffusion coefficient of the electrochemically active species via Levich analysis, as it is —at least to some extent— a balance between kinetic interference and overpotential‐related blocking effects. Estimating the diffusion coefficient of **MeO‐TAA‐Vi‐DMAE** (**9**) from the Levich slope $m_{\mathrm{KL}}$ (at *η* = 0.72 V) = 1.8 × 10^−6^ A^−1^ min^1/2^ resulted in *D* = 2.52 × 10^−6^ cm^2^ s^−1^ (in 0.1 mol dm^−3^ KCl) (see Hydrodynamic Voltammetry section from Supporting Information). This value falls between the one reported by Loh et al. for 4,4′,4″‐trihydroxytriphenylamine (in 0.1 mol dm^−3^ NaCl),^[^ ^30^ ^]^ namely, 1.1 × 10^−6^ cm^2^ s^−1^ and the one reported by Liang et al. for their viologen‐based BROM, namely, 4.0 × 10^−6^ cm^2^ s^−1^ (in 0.5 mol dm^−3^ KCl).^[^ ^39^ ^]^ This is consistent with the molecular weight and size of **MeO‐TAA‐Vi‐DMAE** (**9**) and the differences in supporting electrolytes.

An important note is that the “two reaction hypothesis” on its own fails to reproduce the continuous slope in the overpotential‐dependent current seen in the experimental data (compare to Figure 5a). The OER on glassy carbon could still be responsible for this effect, especially after observing the relevant voltammograms and considering the low concentration of the redox species (see Fig. S7). This could be combined with an electric resistance of an assumed film on the surface or the RDE. We suggest that further understanding could be gained from revisiting the thin film‐coated RDE theory,^[^ ^33^ ^]^ acknowledging that a more accurate model might necessitate multistep kinetics and possible surface effects due to strong interactions with the organic molecule or its polymers. Other causes for a potential‐dependent current in the mass transfer dominated region could be hydrodynamic, mass transport, and diffusion layer nonidealities. In damaged electrodes this could be merely the result of creep of electrolyte up the plastic shielding.

### 2.7 DFT Calculations for the Selected BROM

**Figure 7:**
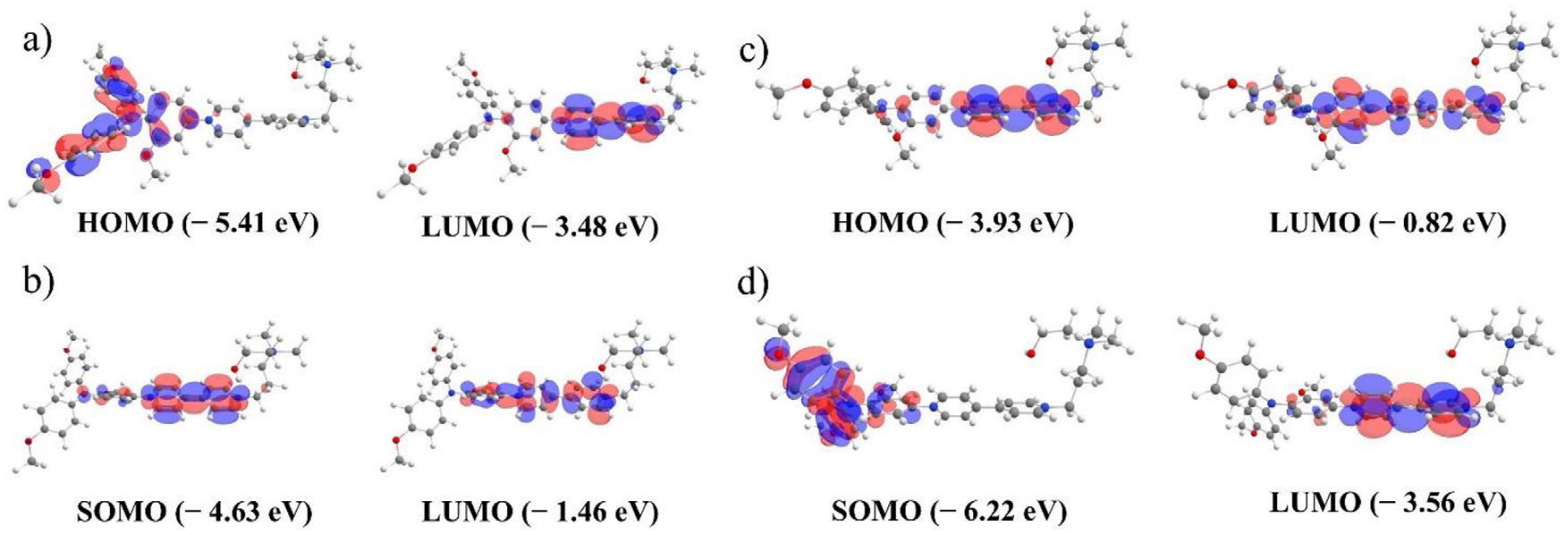
HOMO/LUMO distribution for **MeO‐TAA‐Vi‐DMAE** (**9**) in water; a) (MeO‐TAA‐Vi‐DMAE)^3+^; b) (MeO‐TAA‐Vi‐DMAE)^2+^; c) (MeO‐TAA‐Vi‐DMAE)^+^; d) (MeO‐TAA‐Vi‐DMAE)^4+^.

The results of the DFT calculations of each oxidation state of **MeO‐TAA‐Vi‐DMAE** (**9**) are given in Figure 7. Each oxidation state was initially optimized with XTB.^[^ ^67^, ^68^ ^]^ After that, we used the Conformer‐Rotamer Ensemble Sampling Tool (CREST), which was developed by Grimme et al.^[^ ^67^, ^69^ ^]^ Afterwards, the conformer set was further processed using CENSO, developed by the same group,^[^ ^70^, ^71^ ^]^ to predict the most important conformer according to the Boltzmann distribution. In all calculations, water was incorporated as a solvent. The conformer with the highest Boltzmann weight was further calculated with high‐level DFT with ORCA.^[^ ^72^, ^73^ ^]^ The structure was optimized using the functional PBE0 with a dispersion correction^[^ ^74^, ^75^ ^]^ and the def2‐TZVP basis set^[^ ^76^ ^]^ with a PCM solvent model for water. For all oxidation states, all analytical frequency calculations resulted in positive values. The HOMO‐LUMO levels for the different oxidation states are shown in Figure 7 and Table 1 from the Supporting Information. In the oxidized and nonoxidized form (Figure 7a, d) it is possible to see that the HOMO/SOMO is mainly located in the TAA unit while the LUMOs are located in the viologen unit. In the reduced and double reduced form (Figure 7b, c) the HOMO/SOMO is mainly located in the viologen unit and the attached phenyl ring of the TAA unit. The same can be seen for both LUMOs of the molecules. The energy levels and *λ*_max_ are summarized in Table 1 (Supporting Information). The angles calculated for **MeO‐TAA‐Vi‐DMAE (9),** according to Kim et al. (Figure. S12),^[^ ^56^ ^]^ in all the redox states are summarized in Table 2 (Supporting Information); the values here obtained differ from the derivatives reported in Ref.[56] and likely reflect the presence of a methoxy group in *ortho* to the TAA nitrogen on the linker between the two moieties. Interestingly, the reduction of the viologen moiety seems to have a greater effect on the average *Φ* value than the oxidation of TAA, suggesting that the former is more closely linked to changes in the molecule's overall conformation.

## 3 Conclusions

The exploration of new organic scaffolds for the synthesis of bipolar molecules promises to advance the field of aqueous SOFBs. Here, a class of bipolar molecules is obtained by tethering viologen derivatives to a TAA skeleton. The new derivatives resemble classic push‐pull systems in both nonoxidized and oxidized forms. Through simple and modular synthetic routes, it was possible to evaluate the effect of modifications of both the TAA moiety and the substituent on the viologen side on electrochemical properties. A preliminary screening allowed us to select **MeO‐TAA‐Vi‐DMAE** (**9**) as the best candidate for development. Further characterization revealed that solubility has to be improved, particularly for the reduced state, to explore the use of these compounds in flow cells. Currently, modifications of **MeO‐TAA‐Vi‐DMAE** (**9**) are being screened in our laboratories. Further experiments are being carried out to better understand the electrochemistry of this class of BROMs and to improve the reversibility of the viologen unit (or perhaps replace it). These TAA‐viologen bipolar molecules allow us to draw guidelines and strategies to improve their solubility and stability.

The frequently overlooked potential‐dependent current at high overpotentials in the mass transfer dominated region of RDE experiments was analyzed through a three‐term Koutecký–Levich relation in Ohmic terms. This was possible on the fast, diffusion‐controlled TAA moiety reaction of **MeO‐TAA‐Vi‐DMAE** (**9**) by considering its anodic current at glassy carbon in the mass transfer‐dominated region. This model discarded a purely reversible electron transfer reaction case, while a “reversible reaction + resistance distortion” hypothesis was considered inadequate for this case but could qualitatively explain reported results with damaged electrodes or resistive cell components. Yet, it was shown that a “two reaction hypothesis” gave a comparable current to the experimental data, being consistent with the onset of the **TAA** OER, or perhaps the second oxidation of **MeO‐TAA‐Vi‐DMAE** (**9**), and useful to estimate a diffusion coefficient. Yet, the overall overpotential dependency (i.e., the “slope”, nearly constant for all angular velocities) could not be explained by this model only, pointing out the need to reconsider the electrical and electrolyte resistance of the possible electrode film, revisiting film‐coated electrode models,^[^ ^64^, ^65^, ^66^ ^]^ which have not accounted for this particular effect, to the best of our knowledge. Regarding the viologen moiety of **MeO‐TAA‐Vi‐DMAE** (**9**), a surface interaction process was indeed indicated by reduction peaks at nearly constant potential under forced convection. More research is needed on the possible formation mechanism of electrode films and secondary reactions in AOFBs. Irreversible, resistive electrode fouling could negatively impact the performance of an FB,^[^ ^77^ ^]^ yet some permeable thin films can be ordinary or even beneficial in indefinitely steady electrode reactions.^[^ ^59^ ^]^

## Conflict of Interests

The authors declare no conflict of interest.

## Supporting information



Supporting Information

## Data Availability

The data that support the findings of this study are available in the supplementary material of this article.

## Footnotes

* The traditional Koutecký‐Levich analysis takes current values from the mixed controlled region to extract the kinetic current and construct a Tafel plot for sluggish electrode reactions. Still, Koutecký‐Levich lines can be constructed with current values at any overpotential and for fast reactions to obtain other information, such as in this case.

† At this point, it should be noted that all linear sweep voltammograms of Figure. 6 are presented in terms of overpotential at the abscissa, since a shift by any reference potential is straightforward and does not add additional value to the understanding.
