# Supplementary material for: Toward Symmetric Organic Aqueous Flow Batteries: Triarylamine‐Based Bipolar Molecules and Their Characterization via an Extended Koutecký–Levich Analysis
Source: Chemistry. 2025 May 3;31(31):e202500815. doi: 10.1002/chem.202500815 (PMC12133636; doi:10.1002/chem.202500815)
Supplement: Supplementary file 1 — Supporting Information [file CHEM-31-e202500815-s001.pdf]

Supporting Information

Towards Symmetric Organic Aqueous Flow Batteries: Triarylamine-Based Bipolar Molecules and their Characterization via an Extended Koutecký-Levich Analysis

Carlo Caianiello,[a] Tim Tichter,[b] Luis F. Arenas,[c,d,e,f] René Wilhelm*[a]

[a] M.Sc. C. Caianiello, Prof.-Dr. R. Wilhelm, Institute of Organic Chemistry, Clausthal University of Technology, Leibnizstraße 6, Clausthal-Zellerfeld, 38678, Germany. E-mail: rene.wilhelm@tu-clausthal.de
[b] Dr. T. Tichter, Bundesanstalt für Materialforschung und -prüfung (BAM), Unter den Eichen 87, 12205, Berlin, Germany.
[c] Dr. L.F. Arenas, Institute of Chemical and Electrochemical Process Engineering, Clausthal University of Technology, Leibnizstraße 17, Clausthal-Zellerfeld, 38678, Germany.
[d] Dr. L.F. Arenas, Research Center for Energy Storage Technologies, Clausthal University of Technology, Am Stollen 19A, Goslar 38640, Germany.
[e] Dr. L.F. Arenas, Electrochemical Engineering Laboratory, Department of Mechanical Engineering, University of Southampton, Southampton SO17 1BJ, United Kingdom.
[f] Dr. L.F. Arenas, School of Chemistry and Chemical Engineering, University of Southampton, Southampton SO17 1BJ, United Kingdom.

Table of contents

General Information 2

Solubility measurement 2

Cyclic voltammetry 3

Hydrodynamic voltammetry 3

Starting point - Learning from the synthesis and cyclic voltammetry of TMIPA-Cl 5

Additional figures 6

DFT Results 11

Synthetic procedures 20

NMR spectra 37

References 92

General Information

All substances were purchased from BLD Pharm, Fluorochem, TCI, Acros Organics and Sigma-Aldrich. Potassium carbonate was dried by storing it in an oven at 120 °C. Reactions under microwave irradiations were conducted with a Discover SP microwave reactor (CEM Corp.) in a sealed tube with a PTFE cap. Pressure flasks were either purchased from FengtecEx GmbH or in-house-made. Unless otherwise stated, the solvents were either reagent grade or HPLC suitable. Amberlite® IRA-900Cl was purchased from Thermo Fisher Scientific and regenerated as described below. HPLC-grade water from Carl Roth was used in the preparation of the solutions for the electrochemical characterization (conductivity of 18.2 MΩ cm). Melting points were measured with a Stuart Scientific SMP3. NMR data were collected with either an AVANCE NEO 400 MHz or an AVANCE III 600 MHz instrument from Bruker (Bruker BioSpin GmbH & Co. KG). Mass spectra (HRESI-MS) were acquired in an LC/MSD-System Serie HP1100 (API-ES) spectrometer (Agilent). A Bruker Alpha FT-IR spectrometer (platinum ATR) was used to record IR data. GC-MS measurements were carried out on a Varian 320 MS TQ mass spectrometer. UV-Vis characterization was performed on a Jasco V-650 spectrophotometer using a high-precision cell (quartz Suprasil®) from HellmaAnalytics (10 mm light path).

Anion exchange general procedure: In a typical procedure, the corresponding salt (e.g., iodides, bromides or a mixture of bromide and chloride) is dissolved in MeOH and passed three times through a column filled with an excess of IRA900-Cl (25-30 times by mass). The MeOH was removed under reduced pressure and the chloride salt was dried under a high vacuum. The IRA-900Cl was regenerated after each use. All the salts prepared were subjected to anion exchange before the electrochemical characterization to avoid any effect from the presence of I^- or Br^-.

IRA-900Cl regeneration procedure: The IRA-900Cl was purified by Soxhlet extraction with MeOH for at least 24 hours. Afterwards, the resin was then regenerated by flushing it with 2-4 bed volumes (BV) of 15 % NaCl solution, followed by deionized water (4 BV).

Solubility measurement

Solubility measurement was carried out according to a known procedure.[1] A calibration curve was built by using three different concentrations (1, 5, 10 µM) and by taking the absorbance at 277 nm (Fig. S8-9). A saturated solution was prepared by adding a minimum

amount of HPLC-grade water to an excess of **9** (≈1.3 g). Note: the solution is very dark and viscous, the latter being responsible for the high standard deviation. An aliquot of the saturated solution was taken and properly diluted until the absorbance fell into the range obtained with the calibration curve (dilution factor between 100000 and 125000). The reported value is the average of three measurements.

Cyclic voltammetry

All the experiments were performed with an Autolab PGSTAT204 potentiostat/galvanostat (Metrohm) in a three-electrode cell configuration. Before each measurement, the glassy carbon working electrode (d = 2mm) was polished with a 0.03 µm Al_2O_3 slurry and then rinsed carefully with deionized water. A platinum sheet was used as a counter electrode and the reference was an Ag/AgCl (3.0 mol dm^{-3} KCl) electrode. All measurements were carried out using 0.1 mol dm^{-3} or 1 mol dm^{-3} as the aqueous supporting electrolyte at a temperature of 22 °C under a nitrogen atmosphere after purging the solutions from O_2 for 30 min.

Hydrodynamic voltammetry

The procedure was performed with a concentration of 1 mM in 0.1 M KCl using an RDE II (Metrohm) and an Autolab PGSTAT204 potentiostat/galvanostat (Metrohm) in a 100 mL conical, undivided cell. A glassy carbon electrode (d = 3 mm) was used as the working electrode, a platinum sheet as the counter electrode and an Ag/AgCl (3.0 M KCl) electrode as the reference. The working electrode was polished with an Al_2O_3 slurry from Metrohm before each measurement. Linear sweep voltammetry was carried out at a temperature of 22 °C and v = 5 mV s^{-1}. All solutions were deoxygenated for 30 min by bubbling nitrogen and the measurements were performed under a blanket of nitrogen. The PEEK-coated electrode was in good condition, having been recently acquired. All cell connections and cables were free of corrosion, dirt, or any visible damage.

IR compensation for the electrolyte solution was initially performed using the positive feedback method in the potentiostat/galvanostat implemented through the Nova 2.1.7 software (Metrohm). As shown in Fig. S10, experiments revealed a negligible effect of IR compensation on the RDE data. Hence the rest of the RDE data was obtained uncompensated, which is reasonable in view of the 0.1 M KCl supporting electrolyte having a relatively high conductivity and the short distance between working and reference electrodes.

As explained in the main text, the values of *D* were calculated from the slope m_{KL} = 1.8×10^{-6} A^{-1} min$^{1/2}$ at η = 0.72 V (this slope being equivalent to 1.72×10^{-6} A rad$^{-1/2}$ s$^{1/2}$ in the typical plot of current density vs square root of angular velocity in radians per second) and considering the linear relationship between limiting current, I_L, and the square root of rotation rate, $\omega^{1/2}$, in accordance with the Levich equation:

$$I_L = 0.62nAD^{2/3}c\nu^{-1/6}\omega^{1/2}$$

where *n* is the number of electrons involved in the reaction, *F* is the Faraday constant, *c* is the concentration of the species in solution and ν is the kinematic viscosity of the solution, which is 0.0098 cm^2 s^{-1} for 0.1 mol dm^{-3} KCl. The kinematic viscosity was calculated from ref. [2] and the density of the solution from ref. [3].

Starting point - Learning from the synthesis and cyclic voltammetry of TMIPA-Cl

The starting point of our investigation was inspired by a quaternized tris (4-(1H-imidazol-1-yl)phenyl) amine (TIPA), or formally 1,1',1''-(nitrilotris(benzene-4,1-diyl))tris(3-methyl-1H-imidazol-3-ium), reported as an *N*-heterocyclic carbene precursor.[4] This TIPA was an iodide salt and, to our knowledge, its electrochemical characterization in aqueous media has not been reported. Given our interest in synthesizing new water-soluble ROMs, we produced the quaternized TIPA as a chloride salt (TMIPA-Cl, Fig. S4a) and assessed it by using cyclic voltammetry (Fig. S1). TMIPA-Cl showed an oxidation at +1.15 V *vs* Ag/AgCl with a current peak ratio approaching unity (≈ 1.30) and its voltammogram did not change significantly upon increasing its concentration to 0.1 mol dm^{-3} (Fig. S1b, c). However, such a high oxidation potential makes the molecule unsuitable for a posolyte, since it would lead to an open circuit voltage (OCV) larger than the electrochemical window of water when combined with common negolytes such as those based on methyl viologen (MV), BTMAP-Vi[5] or (DMAE-Pr)$_2$-Vi[1] (≈ 1.80 V and ≈ 1.65 V respectively).

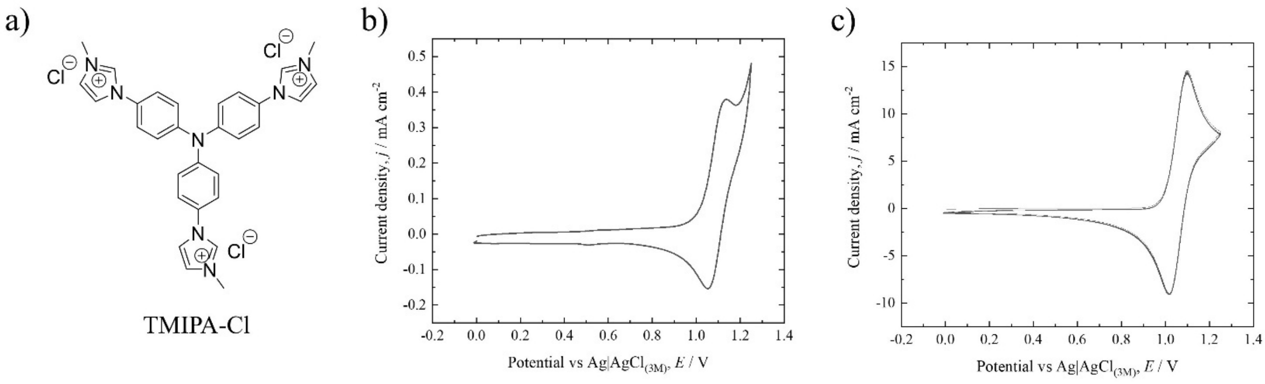

Fig. S1 Starting point of this investigation. a) Structure of TMIPA-Cl. Corresponding voltammograms were recorded at b) 0.002 mol dm^{-3} in 0.1 mol dm^{-3} KCl and at c) 0.1 mM in 1 mol dm^{-3} KCl. Scan rate 100 mV s^{-1}.

Additional figures

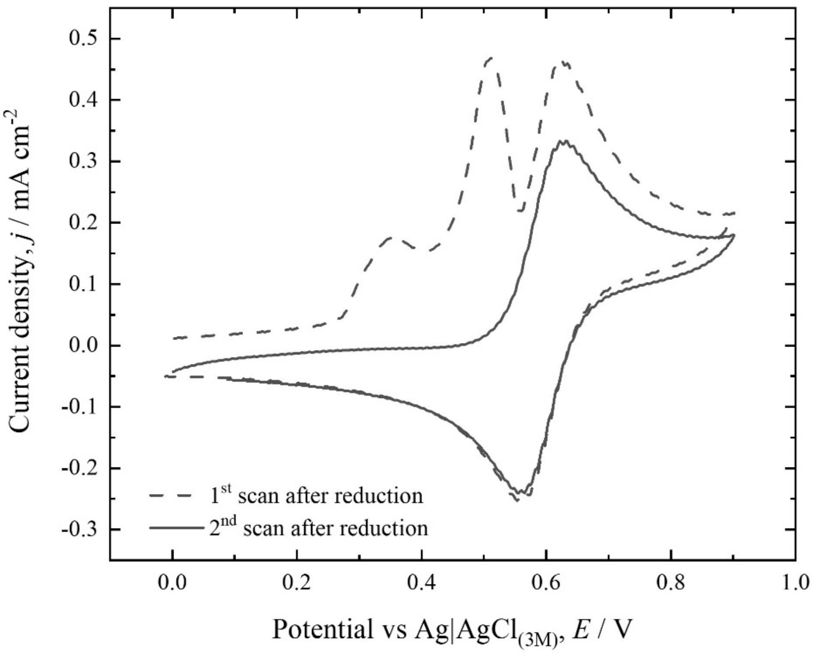

Fig. S2 Two consecutive scans for compound **4** after reducing the pyridinium units. Scan rate 100 mV s^{-1}.

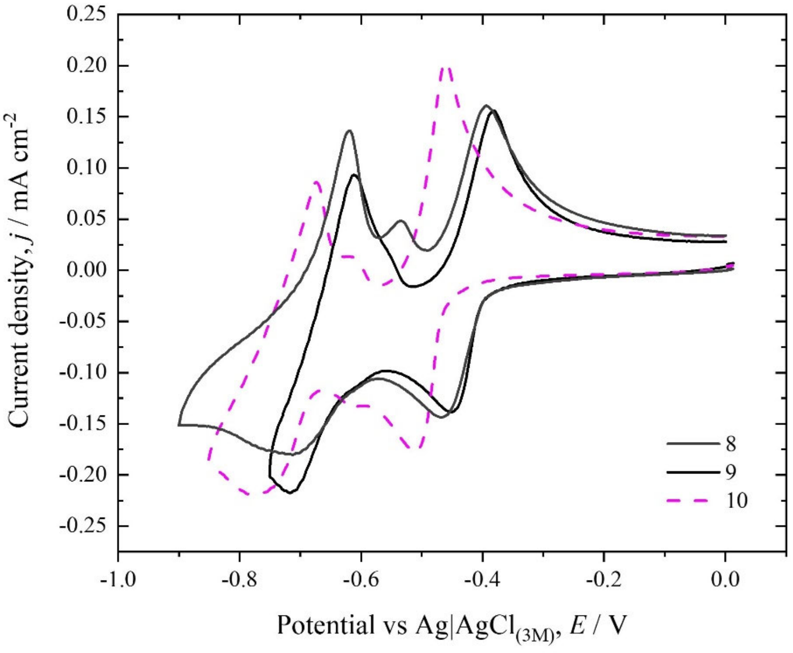

Fig. S3 Second reduction for the viologen moiety for compounds **8-10** recorded at 0.001 mol dm^{-3} in 0.1 mol dm^{-3} KCl. Scan rate 100 mV s^{-1}.

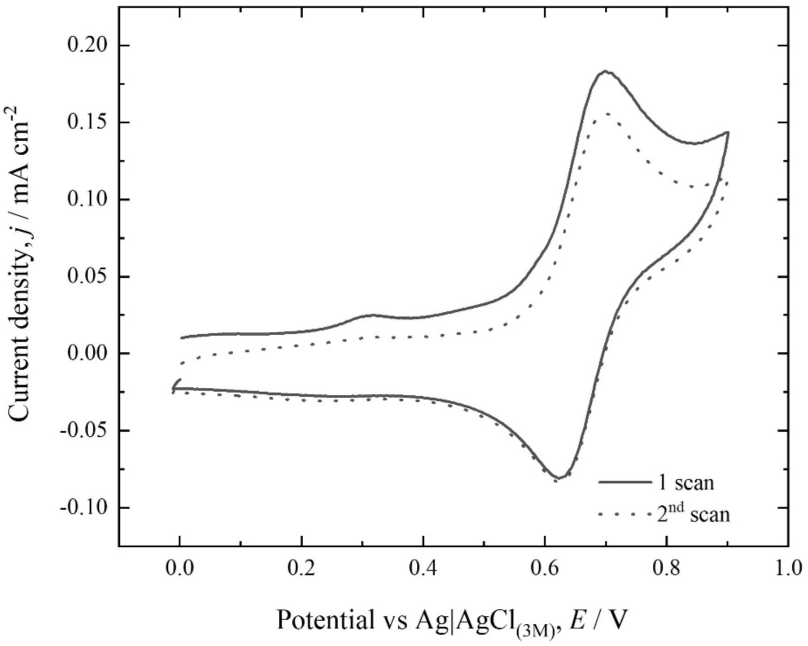

Fig. S4 Compound **8** voltammograms recorded at 0.001 mol dm^{-3} in 0.1 mol dm^{-3} KCl, two successive scans. Scan rate 100 mV s^{-1}.

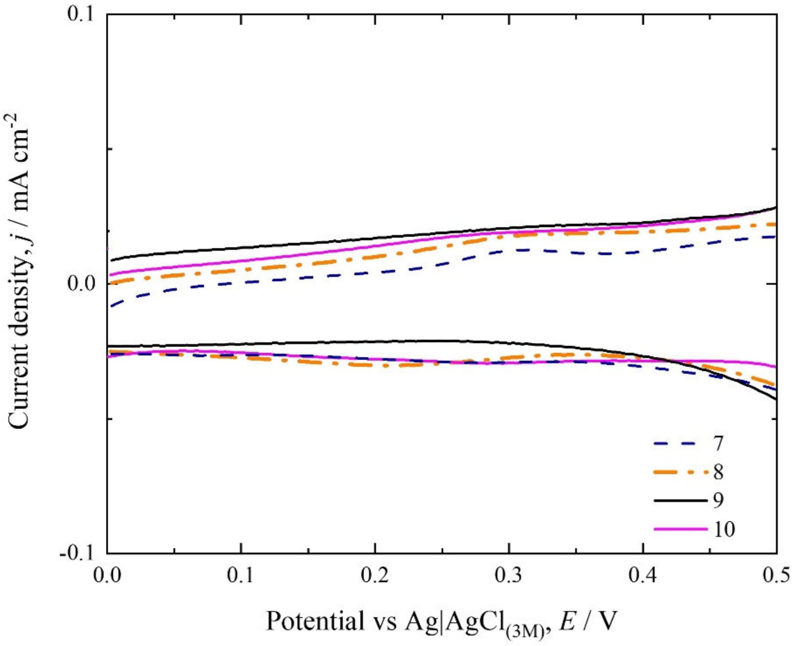

Fig. S5 Enlarged section of the voltammograms for compounds **7-10**. Scan rate 100 mV s^{-1}.

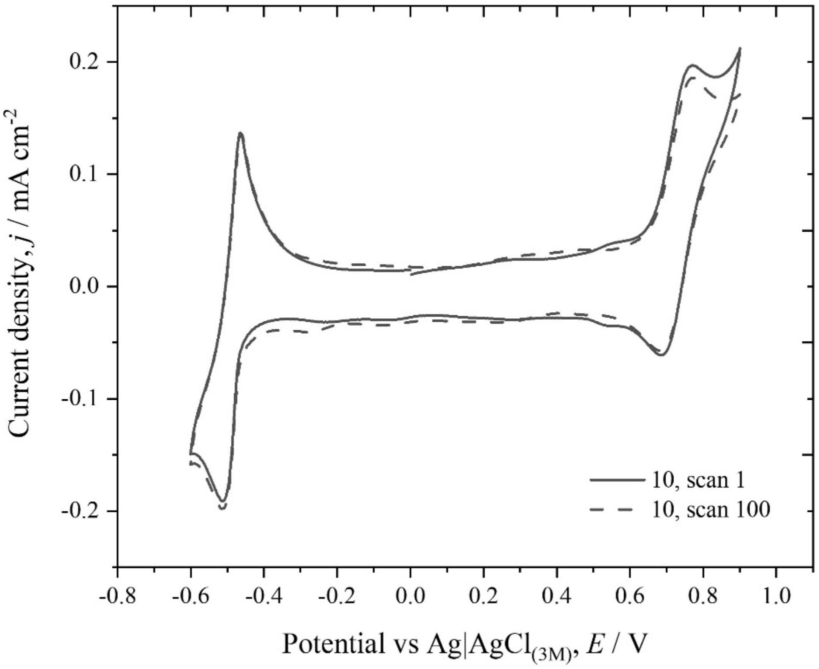

Fig. S6 Selected scans for **10** recorded at 0.001 mol dm^{-3} in 0.1 mol dm^{-3} KCl. Scan rate 100 mV s^{-1}.

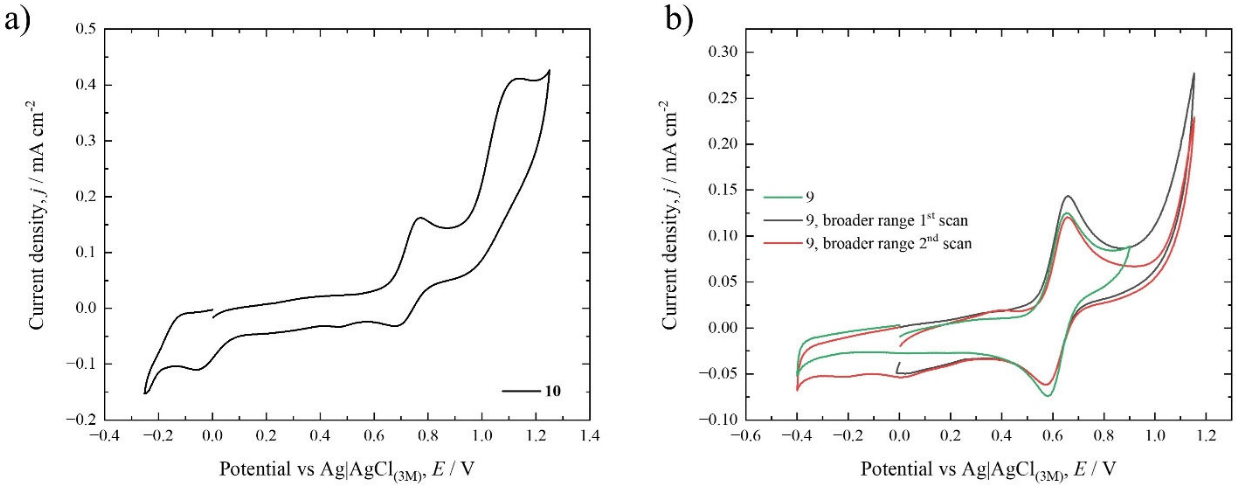

Fig. S7 Comparison voltammograms for **10** and **9** with a broader voltage range recorded at 0.001 mol dm^{-3} in 0.1 mol dm^{-3} KCl. Scan rate 100 mV s^{-1}. a) compound **10;** b) compound **9**, for which the 2nd oxidation might be masked due to an overlap with OER, which at GC has a peak at +1.2 V vs Ag/AgCl under neutral conditions.[6]

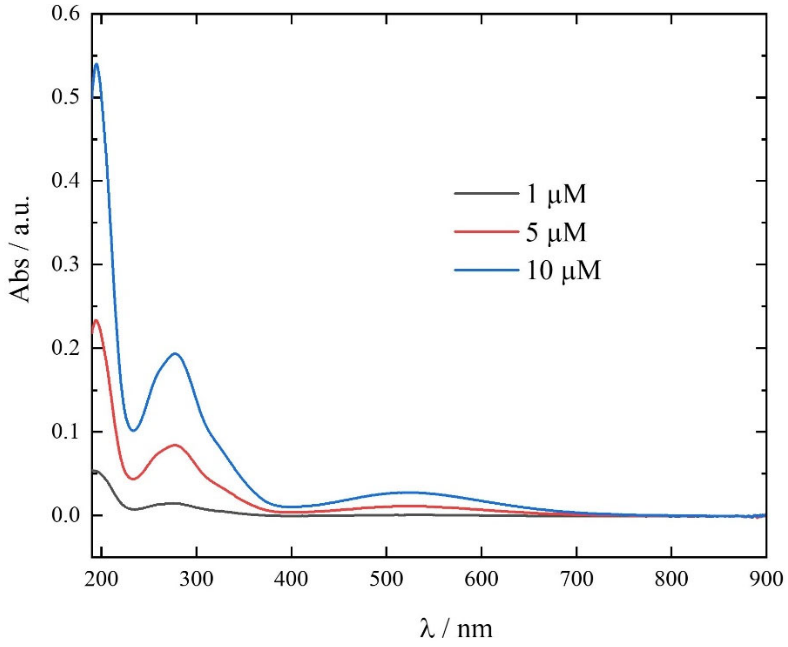

Fig. S8 UV-Vis spectra for compound **9** in HPLC-grade water at varying concentrations.

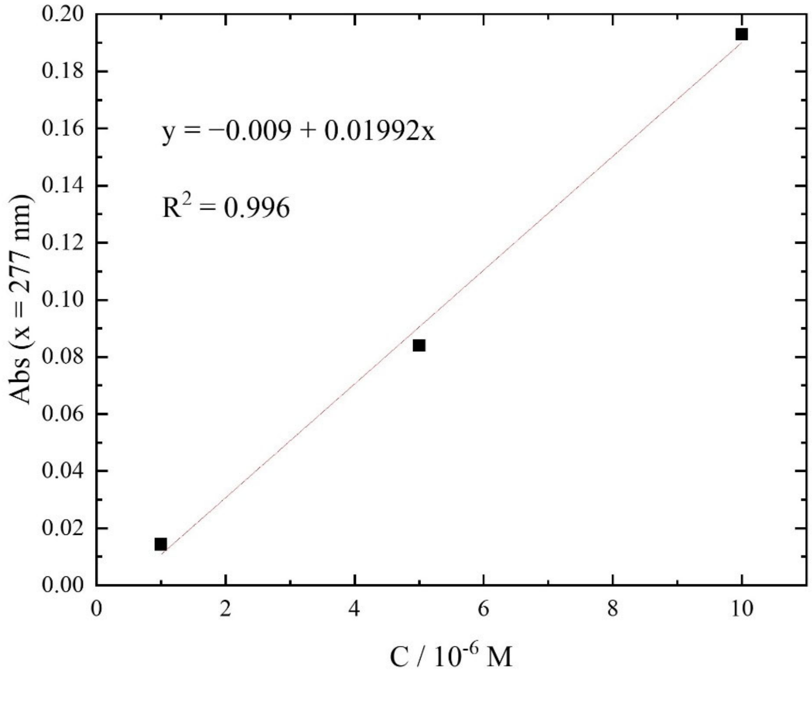

Fig. S9 Calibration curve for compound **9**.

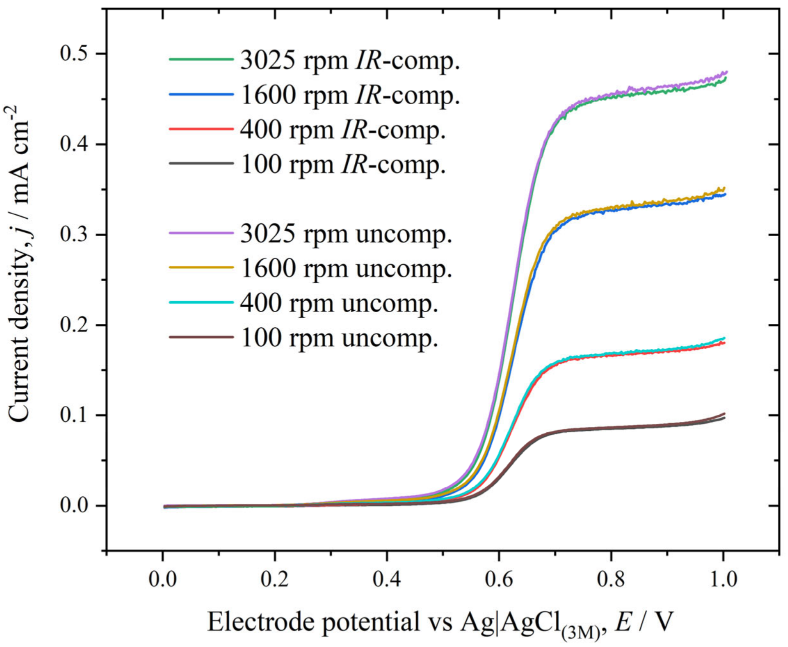

Fig. S10 Comparison of $IR_{solution}$-compensated and uncompensated raw RDE-data. Hydrodynamic linear sweep voltammetry for the oxidation of compound MeO-TAA-Vi-DMAE **(9)** at RDE. Concentration 0.001 mol dm^{-3} in 0.1 mol dm^{-3} KCl. Scan rate 5 mV s^{-1}.

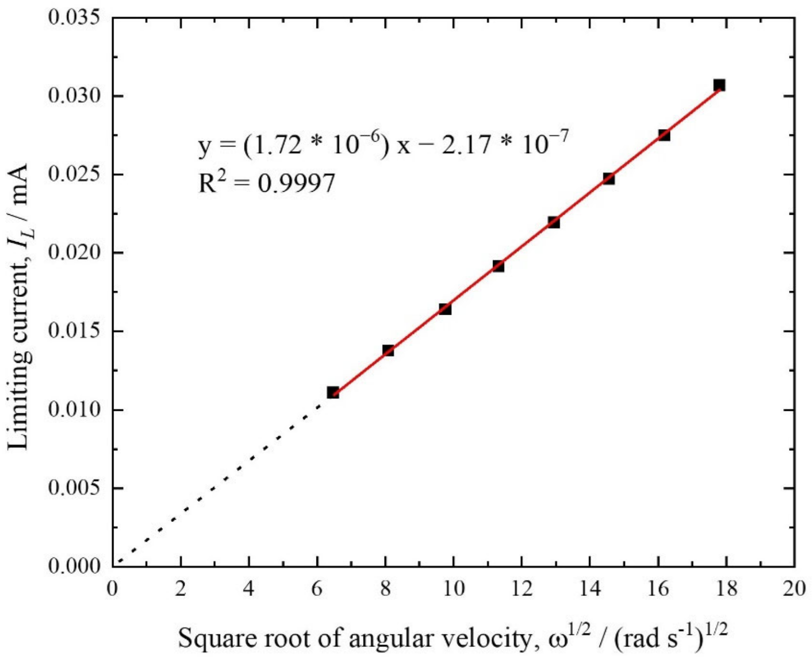

Fig. S11 Levich plot for the determination of the diffusion coefficient from the linear sweep voltammetry for **MeO-TAA-Vi-DMAE** (**9**).

DFT Results

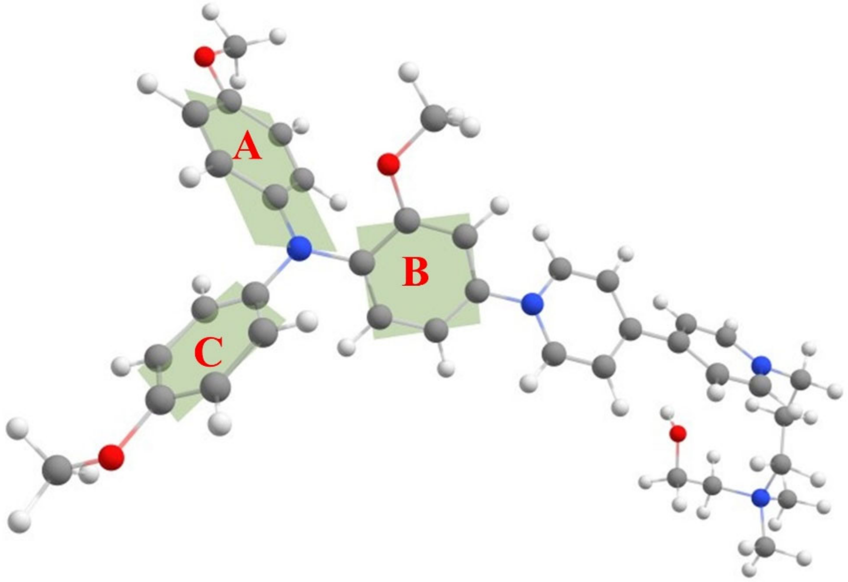

Fig. S12 Planes A, B, C used for calculating dihedral angles in (**MeO-TAA-Vi-DMAE**)$^{3+}$

Table S1 Energy levels and calculated UV/Vis for **MeO-TAA-Vi-DMAE** (**9**) in different oxidation states.

Compound	HOMO/SOMO (eV), Φ_{NHE} (V vs NHE)	LUMO (eV), Φ_{NHE} (V vs NHE)	λ_{max} (nm)
9^{3+}	− 5.41, + 0.56	− 3.48, +1.37	769, 432
9^{2+}	− 4.63 (SOMO), − 0.22	− 1.46, − 3.39	653, 525
9^{+}	− 3.93, − 0.92	− 0.82, − 4.03	449, 427
9^{4+}	− 6.22 (SOMO), + 1.37	− 3.56, − 1.29	692, 560, 446

Table S2 Dihedral angles for **MeO-TAA-Vi-DMAE (9)** in all the oxidation states

**236** oxidation state	Φ1 (°)	Φ2 (°)	Average (°)
$\mathbf{236}^{3+}$	69.4	72.8	71.1
$\mathbf{236}^{2+}$	72.2	75.6	73.9
$\mathbf{236}^{+}$	75.8	76.8	76.3
$\mathbf{236}^{4+}$	71.5	70.6	71.1

[Compound 9]$^{3+}$

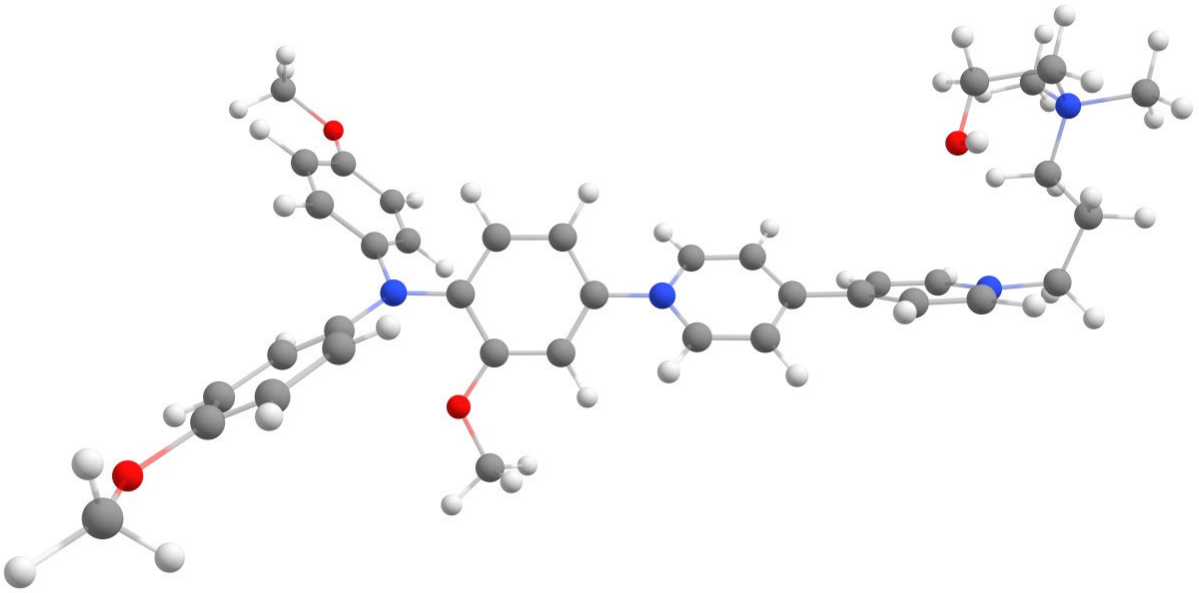

! PBE0 def2-TZVP TIGHTSCF D3BJ CPCM(water) Opt

HOMO: -5.4185; LUMO: -3.4836

XYZ-Coordinates

6	1.250372000	-0.457583000	0.926489000	6	5.450296000	0.223728000	1.740753000
6	2.616668000	-0.378355000	0.999136000	6	-0.909492000	0.491089000	0.478755000
6	3.258502000	0.826438000	0.723247000	6	-1.711273000	1.504136000	0.991755000
6	2.472121000	1.923278000	0.377733000	6	-3.089914000	1.389747000	0.920527000
6	1.109314000	1.792651000	0.305726000	6	-3.672364000	0.256542000	0.300343000
7	0.512694000	0.614585000	0.575367000	6	-2.829006000	-0.729766000	-0.204441000
6	4.722849000	0.928176000	0.785021000	6	-1.453658000	-0.631917000	-0.117046000
6	5.420633000	1.725096000	-0.120570000	7	-5.054833000	0.108739000	0.236107000
6	6.790076000	1.770761000	-0.066780000	6	-5.598427000	-1.195295000	0.336323000
7	7.466330000	1.073669000	0.858217000	6	-5.871017000	1.150321000	-0.254246000
6	6.819898000	0.320858000	1.758732000	6	-7.171591000	1.300461000	0.229637000

6	-7.985893000	2.305986000	-0.245645000	1	-8.224495000	-6.578911000	0.130009000
6	-7.515852000	3.202689000	-1.207128000	1	-8.894207000	-4.981448000	-0.287620000
6	-6.218022000	3.066186000	-1.686758000	1	-7.498275000	-5.629722000	-1.191470000
6	-5.411586000	2.035705000	-1.217926000	1	-7.656229000	4.621868000	-3.499216000
6	-6.458384000	-1.696936000	-0.629932000	1	-8.759615000	5.787655000	-2.726962000
6	-6.990924000	-2.974936000	-0.518409000	1	-7.070186000	5.663268000	-2.173855000
6	-6.645100000	-3.777252000	0.564440000	1	9.237214000	2.121848000	0.605264000
6	-5.774947000	-3.278069000	1.535434000	1	9.286342000	0.853566000	1.835820000
6	-5.267255000	-2.000528000	1.426301000	1	9.045911000	0.355732000	-1.175829000
8	-7.099704000	-5.035597000	0.759281000	1	10.568856000	0.391283000	-0.294400000
6	-7.978389000	-5.574960000	-0.209846000	1	9.940252000	-1.510864000	1.081805000
8	-8.386402000	4.158681000	-1.608819000	1	8.287478000	-1.510441000	0.458719000
6	-7.929951000	5.104557000	-2.556090000	1	10.358301000	-3.683037000	0.623868000
6	8.939358000	1.099530000	0.832584000	1	8.632556000	-3.803337000	0.211474000
6	9.508438000	0.148522000	-0.209286000	1	9.865861000	-4.406456000	-0.933216000
6	9.324104000	-1.294205000	0.207598000	1	9.154439000	-3.122142000	-2.658540000
7	9.713194000	-2.309030000	-0.827786000	1	9.075182000	-1.362649000	-2.598741000
6	9.632484000	-3.651136000	-0.185835000	1	11.397642000	-2.916751000	-1.923278000
6	8.834140000	-2.274477000	-2.051757000	1	11.189927000	-1.152688000	-1.805303000
6	11.119423000	-2.094433000	-1.267261000	1	11.758474000	-2.079886000	-0.385844000
6	7.340679000	-2.353588000	-1.811937000	1	7.090552000	-3.106444000	-1.062910000
8	6.761010000	-1.139586000	-1.382671000	1	6.897518000	-2.686142000	-2.755925000
8	-3.934365000	2.284627000	1.451621000	1	6.730864000	-0.535083000	-2.131950000
6	-3.392594000	3.455586000	2.037543000	1	-4.245141000	4.048833000	2.359871000
1	0.705679000	-1.360021000	1.162627000	1	-2.768744000	3.211114000	2.901950000
1	3.171097000	-1.267198000	1.269300000	1	-2.805674000	4.020898000	1.308397000
1	2.908762000	2.888784000	0.159964000				
1	0.461629000	2.604916000	0.009049000				
1	4.912985000	2.290850000	-0.890365000				
1	7.381732000	2.354899000	-0.759364000				
1	7.429193000	-0.195806000	2.488504000				
1	4.965071000	-0.388612000	2.489134000				
1	-1.259984000	2.343545000	1.502831000				
1	-3.271098000	-1.589683000	-0.691547000				
1	-0.823327000	-1.395584000	-0.555117000				
1	-7.543240000	0.618925000	0.985830000				
1	-8.996099000	2.421403000	0.130263000				
1	-5.824851000	3.741752000	-2.434613000				
1	-4.406933000	1.928592000	-1.611377000				
1	-6.723113000	-1.083996000	-1.483469000				
1	-7.660697000	-3.334142000	-1.288142000				
1	-5.518486000	-3.904343000	2.382142000				
1	-4.602109000	-1.616136000	2.191270000				

[Compound 9]$^{2+}$

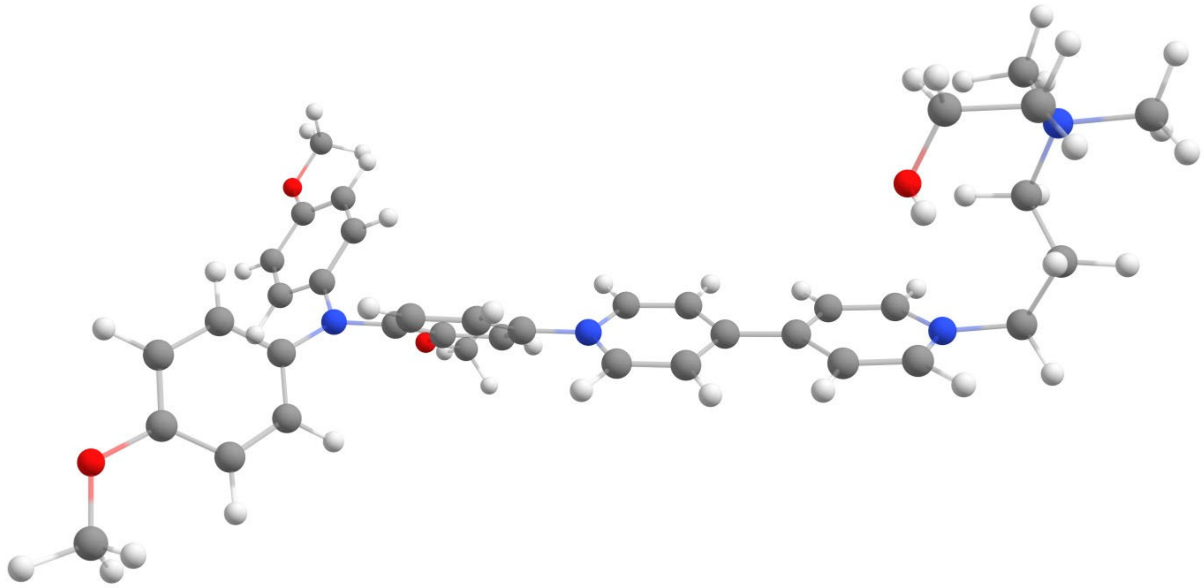

SOMO: -4.6266; LUMO: -1.4637

XYZ-Coordinates

6	1.114400000	-0.925151000	0.980412000	6	-6.927409000	-3.350312000	-1.323982000
6	2.456126000	-0.956585000	1.171579000	6	-7.639994000	-2.430989000	-0.551831000
6	3.221850000	0.236589000	1.282056000	6	-7.043858000	-1.263492000	-0.122851000
6	2.467422000	1.438457000	1.188719000	6	-6.688191000	1.737368000	-1.082377000
6	1.123489000	1.422214000	1.008960000	6	-7.368226000	2.939184000	-1.103182000
7	0.426595000	0.252466000	0.897262000	6	-7.171806000	3.882025000	-0.094707000
6	4.631652000	0.225567000	1.459806000	6	-6.287147000	3.594805000	0.940973000
6	5.408917000	1.415936000	1.490957000	6	-5.624097000	2.375703000	0.965063000
6	6.759872000	1.371769000	1.617433000	8	-7.873800000	5.034569000	-0.203361000
7	7.428124000	0.195595000	1.734057000	6	-7.682169000	6.016646000	0.795781000
6	6.723396000	-0.963933000	1.743736000	8	-7.590245000	-4.476570000	-1.683586000
6	5.371330000	-0.978342000	1.613341000	6	-6.891691000	-5.422556000	-2.467997000
6	-0.975998000	0.259937000	0.677902000	6	8.886999000	0.190972000	1.770048000
6	-1.767574000	-0.690644000	1.315736000	6	9.509609000	0.366526000	0.391147000
6	-3.137962000	-0.707764000	1.104163000	6	9.232620000	-0.838312000	-0.480547000
6	-3.726850000	0.227626000	0.226146000	7	9.734711000	-0.733654000	-1.894285000
6	-2.909364000	1.170912000	-0.382304000	6	9.491373000	-2.045038000	-2.555487000
6	-1.542203000	1.202613000	-0.165264000	6	9.075997000	0.373592000	-2.675346000
7	-5.109509000	0.210857000	-0.003705000	6	11.201625000	-0.482239000	-1.905712000
6	-5.808499000	1.435724000	-0.042006000	6	7.570995000	0.303741000	-2.823381000
6	-5.722028000	-0.969465000	-0.466164000	8	6.846233000	0.604701000	-1.651847000
6	-5.024460000	-1.879345000	-1.248993000	8	-3.971813000	-1.561987000	1.718944000
6	-5.610830000	-3.067978000	-1.668199000	6	-3.415928000	-2.545044000	2.573507000

1	0.531288000	-1.825360000	0.851902000
1	2.921413000	-1.931282000	1.207046000
1	2.936114000	2.408088000	1.279271000
1	0.536440000	2.328287000	0.972188000
1	4.952662000	2.391662000	1.405110000
1	7.369698000	2.265501000	1.628546000
1	7.301806000	-1.870624000	1.862585000
1	4.884132000	-1.942342000	1.638188000
1	-1.317126000	-1.379980000	2.015974000
1	-3.360910000	1.887234000	-1.058052000
1	-0.925693000	1.923766000	-0.686400000
1	-3.999207000	-1.668323000	-1.530838000
1	-5.031052000	-3.755873000	-2.269022000
1	-8.665056000	-2.656664000	-0.280230000
1	-7.604565000	-0.568862000	0.491678000
1	-6.841047000	1.021059000	-1.880899000
1	-8.051588000	3.171128000	-1.912217000
1	-6.116199000	4.302490000	1.741179000
1	-4.945486000	2.155075000	1.781367000
1	-6.639810000	6.348306000	0.830857000
1	-8.319739000	6.855375000	0.523204000
1	-7.974640000	5.642484000	1.781611000
1	-6.010118000	-5.800847000	-1.941372000
1	-7.585738000	-6.241513000	-2.646693000
1	-6.581555000	-4.992457000	-3.425377000
1	9.208003000	1.005569000	2.419327000
1	9.203853000	-0.747935000	2.225706000
1	9.123292000	1.284151000	-0.056737000
1	10.582009000	0.500599000	0.543183000
1	9.714644000	-1.723988000	-0.062342000
1	8.163701000	-1.026313000	-0.560026000
1	8.440953000	-2.307982000	-2.467020000
1	9.779247000	-1.964573000	-3.601799000
1	10.100491000	-2.794188000	-2.053741000
1	9.532168000	0.336472000	-3.665939000
1	9.370737000	1.313178000	-2.207658000
1	11.549400000	-0.551315000	-2.934281000
1	11.399941000	0.511549000	-1.513582000
1	11.685321000	-1.239618000	-1.291379000
1	7.248685000	-0.686347000	-3.148223000
1	7.320923000	1.000063000	-3.631271000
1	6.938019000	1.544287000	-1.461773000
1	-2.712993000	-3.183854000	2.031586000
1	-2.908873000	-2.085869000	3.427006000

1	-4.253843000	-3.141771000	2.926962000

[Compound 9]$^{1+}$

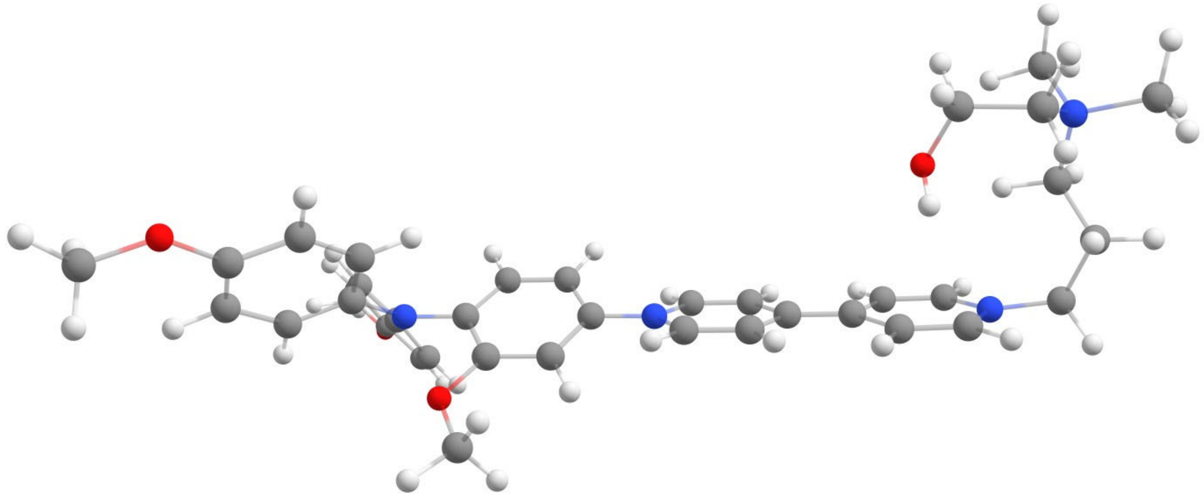

HOMO: -3.9265; LUMO: -0.8190

XYZ-Coordinates

6	1.293812000	-0.636163000	-1.229855000	6	-5.513846000	2.285064000	-0.865023000
6	2.625985000	-0.569331000	-1.398641000	6	-4.840229000	-2.127030000	1.025773000
6	3.389613000	0.631694000	-1.133408000	6	-5.391276000	-3.348941000	1.363081000
6	2.562224000	1.726403000	-0.671789000	6	-6.709985000	-3.648323000	1.030454000
6	1.232443000	1.614356000	-0.510921000	6	-7.461969000	-2.704021000	0.337264000
7	0.541942000	0.440732000	-0.779991000	6	-6.895884000	-1.486931000	-0.014313000
6	4.753895000	0.724267000	-1.300197000	8	-7.171340000	-4.865079000	1.415883000
6	5.518761000	1.922550000	-1.014907000	6	-8.504765000	-5.193348000	1.083670000
6	6.857483000	1.971009000	-1.156008000	8	-7.804364000	4.869705000	0.201078000
7	7.594066000	0.895078000	-1.592859000	6	-8.781268000	5.091539000	1.198605000
6	6.915902000	-0.258868000	-1.903307000	6	9.041395000	0.917443000	-1.612341000
6	5.577099000	-0.369468000	-1.777425000	6	9.663989000	0.042111000	-0.529719000
6	-0.840557000	0.346992000	-0.587142000	6	9.310235000	0.558976000	0.847647000
6	-1.600144000	-0.520019000	-1.380027000	7	9.577755000	-0.403490000	1.977446000
6	-2.973153000	-0.619679000	-1.206758000	6	9.368010000	0.328420000	3.253925000
6	-3.619525000	0.145770000	-0.219031000	6	8.697042000	-1.624151000	1.922047000
6	-2.848074000	0.998762000	0.556098000	6	10.992490000	-0.860787000	1.926986000
6	-1.479459000	1.114170000	0.385825000	6	7.230715000	-1.432349000	2.249875000
7	-5.014382000	0.057598000	-0.037615000	8	6.518545000	-0.568193000	1.397970000
6	-5.583955000	-1.172725000	0.329248000	8	-3.760397000	-1.410957000	-1.961034000
6	-5.752214000	1.253901000	0.047592000	6	-3.149948000	-2.209469000	-2.956491000
6	-6.709061000	1.458517000	1.034980000	1	0.748925000	-1.554165000	-1.394890000
6	-7.423485000	2.647942000	1.110444000	1	3.117215000	-1.476148000	-1.726097000
6	-7.171029000	3.670512000	0.202717000	1	2.995625000	2.694205000	-0.456552000
6	-6.204751000	3.477051000	-0.784699000	1	0.626069000	2.453220000	-0.202736000

1	5.028326000	2.824725000	-0.675277000	
1	7.427751000	2.864294000	-0.934256000	
1	7.524838000	-1.076160000	-2.269474000	
1	5.133456000	-1.312279000	-2.068565000	
1	-1.122292000	-1.077413000	-2.172324000	
1	-3.341020000	1.583342000	1.324719000	
1	-0.915207000	1.766221000	1.039168000	
1	-6.907166000	0.678612000	1.760450000	
1	-8.161186000	2.766890000	1.892893000	
1	-6.012714000	4.273242000	-1.495170000	
1	-4.770208000	2.146647000	-1.641641000	
1	-3.814992000	-1.909460000	1.301835000	
1	-4.805486000	-4.084761000	1.902598000	
1	-8.486230000	-2.906603000	0.053345000	
1	-7.490938000	-0.768438000	-0.565573000	
1	-8.680713000	-6.191259000	1.480953000	
1	-9.211892000	-4.491738000	1.537284000	
1	-8.653921000	-5.202948000	-0.000636000	
1	-9.171589000	6.092552000	1.025159000	
1	-9.596613000	4.365258000	1.124506000	
1	-8.344003000	5.040311000	2.200623000	
1	9.394271000	0.572040000	-2.587666000	
1	9.357751000	1.955535000	-1.498190000	
1	9.310504000	-0.980823000	-0.676871000	
1	10.745445000	0.032490000	-0.678620000	
1	9.893599000	1.451566000	1.079628000	
1	8.252110000	0.802350000	0.918527000	
1	8.380703000	0.783279000	3.249194000	
1	9.462450000	-0.374901000	4.079443000	
1	10.129579000	1.101954000	3.328521000	
1	9.113203000	-2.323197000	2.649793000	
1	8.822038000	-2.061620000	0.932357000	
1	11.130262000	-1.503171000	1.060673000	
1	11.635936000	0.014620000	1.856525000	
1	11.206003000	-1.415197000	2.838337000	
1	7.109018000	-1.040732000	3.260626000	
1	6.802594000	-2.441876000	2.247624000	
1	6.461451000	-0.947053000	0.509870000	
1	-3.955019000	-2.771856000	-3.424921000	
1	-2.650491000	-1.592215000	-3.709097000	
1	-2.426771000	-2.902970000	-2.517731000	

[Compound 9]$^{4+}$

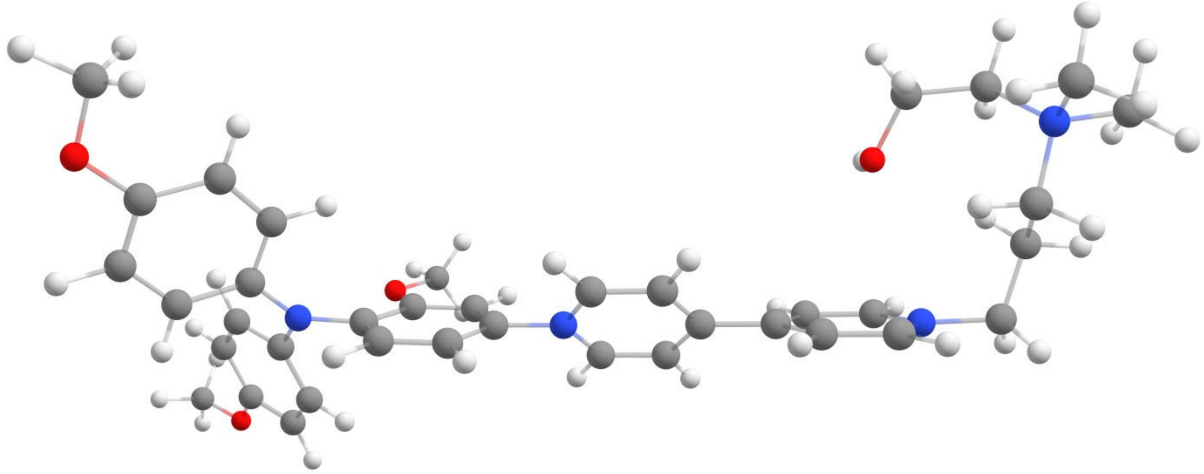

SOMO: -6.2176; LUMO: -3.5622

XYZ-Coordinates

6	1.100622000	-1.952773000	0.069803000	6	-6.380427000	-3.686736000	-0.249997000
6	2.468733000	-2.046777000	0.098231000	6	-7.582923000	-3.421259000	-0.927789000
6	3.227700000	-0.951622000	0.503662000	6	-7.933946000	-2.099969000	-1.230328000
6	2.564884000	0.220122000	0.859285000	6	-7.102822000	-1.071750000	-0.852105000
6	1.196660000	0.273238000	0.793690000	8	-8.318655000	-4.482708000	-1.246553000
7	0.486963000	-0.804329000	0.409909000	6	-9.555828000	-4.281756000	-1.921167000
6	4.696704000	-1.024701000	0.557565000	8	-7.129123000	4.599845000	1.651384000
6	5.396284000	-0.453084000	1.615782000	6	-6.461925000	5.817452000	1.340285000
6	6.766758000	-0.541635000	1.647133000	6	8.911680000	-1.182603000	0.673028000
7	7.437026000	-1.152001000	0.660087000	6	9.515699000	-0.064439000	-0.162341000
6	6.786913000	-1.706576000	-0.373112000	6	9.311092000	1.280740000	0.500378000
6	5.417012000	-1.667354000	-0.445858000	7	9.760178000	2.462967000	-0.306427000
6	-0.947189000	-0.715085000	0.367865000	6	11.195380000	2.329836000	-0.677798000
6	-1.600535000	-1.131822000	-0.778618000	6	8.967065000	2.643215000	-1.575855000
6	-2.984993000	-1.016548000	-0.851919000	6	9.622346000	3.669178000	0.557611000
6	-3.677727000	-0.482443000	0.255317000	6	7.462382000	2.723754000	-1.423679000
6	-2.986679000	-0.083378000	1.392163000	8	6.832167000	1.479678000	-1.201523000
6	-1.613445000	-0.195706000	1.464643000	8	-3.701244000	-1.329504000	-1.926988000
7	-5.072923000	-0.292701000	0.200331000	6	-3.031239000	-1.872139000	-3.059194000
6	-5.909913000	-1.334076000	-0.165480000	1	0.460657000	-2.781340000	-0.199171000
6	-5.581717000	0.953140000	0.557595000	1	2.928255000	-2.989715000	-0.167261000
6	-6.793952000	1.054080000	1.258943000	1	3.105042000	1.108831000	1.157405000
6	-7.280126000	2.285361000	1.610651000	1	0.631874000	1.166241000	1.021021000
6	-6.575887000	3.450769000	1.269904000	1	4.888730000	0.040640000	2.433972000
6	-5.362815000	3.353508000	0.576441000	1	7.354978000	-0.136098000	2.459594000
6	-4.873073000	2.115112000	0.230884000	6	-5.553968000	-2.661607000	0.122366000

1	7.397249000	-2.179049000	-1.131087000	
1	4.927429000	-2.122147000	-1.297022000	
1	-1.033646000	-1.490206000	-1.626634000	
1	-3.545726000	0.305027000	2.234187000	
1	-1.080417000	0.088069000	2.362699000	
1	-7.321162000	0.155986000	1.554907000	
1	-8.200217000	2.377994000	2.174494000	
1	-4.809990000	4.238759000	0.294781000	
1	-3.946531000	2.040150000	-0.324632000	
1	-4.638742000	-2.868925000	0.663728000	
1	-6.130412000	-4.714581000	-0.017427000	
1	-8.837883000	-1.876050000	-1.779252000	
1	-7.352166000	-0.053987000	-1.124171000	
1	-9.393294000	-3.809888000	-2.892817000	
1	-9.980745000	-5.272628000	-2.059177000	
1	-10.229429000	-3.669566000	-1.317524000	
1	-5.478415000	5.851478000	1.814278000	
1	-6.358622000	5.936084000	0.259325000	
1	-7.090886000	6.609116000	1.739295000	
1	9.226387000	-1.122980000	1.714499000	
1	9.212993000	-2.150097000	0.275429000	
1	10.578948000	-0.293432000	-0.250832000	
1	9.088569000	-0.093805000	-1.166120000	
1	8.260764000	1.460363000	0.721288000	
1	9.872808000	1.325761000	1.434908000	
1	11.315435000	1.498776000	-1.367580000	
1	11.507862000	3.255854000	-1.155677000	
1	11.771632000	2.159560000	0.229984000	
1	9.234363000	1.821702000	-2.240760000	
1	9.340036000	3.568309000	-2.016799000	
1	9.891134000	4.546047000	-0.027564000	
1	8.598728000	3.745121000	0.914708000	
1	10.298509000	3.556340000	1.402470000	
1	7.089367000	3.192109000	-2.340014000	
1	7.176498000	3.374645000	-0.596166000	
1	6.843359000	0.982745000	-2.026324000	
1	-3.809651000	-2.075458000	-3.789724000	
1	-2.318090000	-1.152130000	-3.467476000	
1	-2.518548000	-2.799657000	-2.794353000	

Synthetic procedures

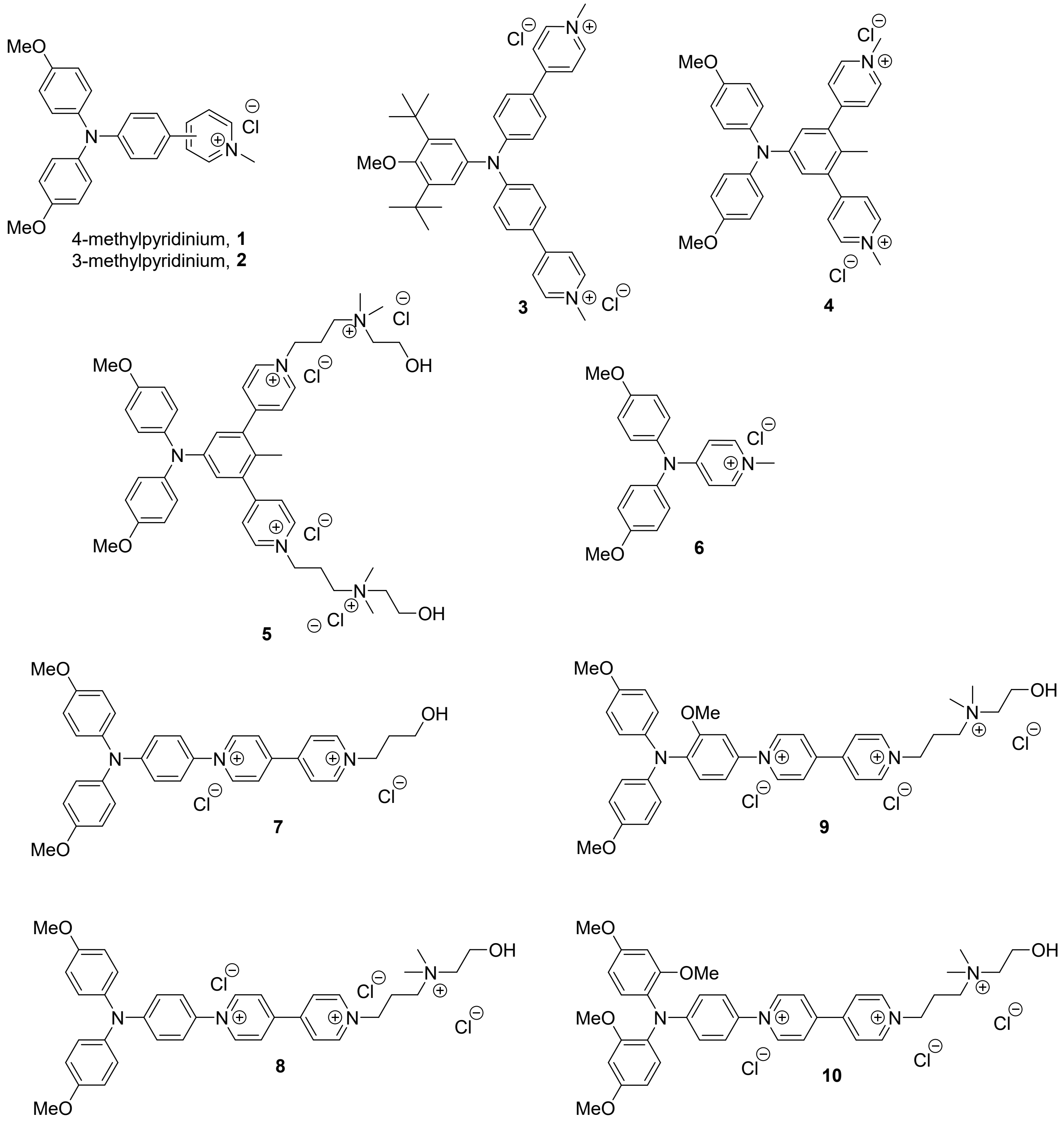

Scheme S1 Summary of the BROMs prepared in this work.

Tris (4-(1H-imidazol-1-yl)phenyl) amine (TIPA)[4] and 1,1',1''-(nitrilotris(benzene-4,1-diyl))tris(3-methyl-1H-imidazol-3-ium) iodide (TMIPA)[7] were prepared according to previous reports. Iodide was exchanged with chloride as described below (See anion exchange procedure).

3-bromo-N-(2-hydroxyethyl)-N,N-dimethylpropan-1-aminium bromide (*DMAE-BPr*) was prepared according to a known procedure.[1]

N,N-bis(4-methoxyphenyl)pyridin-4-amine *((BMP)PA):* It was prepared according to a literature procedure (but increasing the catalyst loading to 40 % and reaction time to 72 h)[8] and purified by hot filtration from PE. Yield: 45 % . Spectroscopic data are consistent with the previous report.[8]

Suzuki coupling starting material preparation:

*N,N-bis(4-bromophenyl)-3,5-di-tert-butyl-4-methoxyaniline (**11**):* It was prepared according to a previous report.[9]

*3,5-dibromo-N,N-bis(4-methoxyphenyl)-4-methylaniline (**12**):* It was prepared by modifying a literature procedure (4-iodoanisole was used instead of 4-tert-butyliodobenzene).[10]

Briefly, 3,5-dibromo-4-methylaniline (1 eq, 1.30 g, 4.90 mmol), copper (I) chloride (0.5 eq, 243 mg, 2.45 mmol), 1,10-phenanthroline (0.52 eq, 460 mg, 2.55 mmol), potassium hydroxide (9 eq, 2.48 g, 44.16 mmol), 4-iodoanisole (2.70 eq, 3.10 g, 13.25 mmol) were added under N_2 in an RBF and degassed by vacuum/N_2 cycles (three times). Anhydrous toluene (12 mL) was then added, and the suspension was refluxed for 24 h. The reaction solution was then cooled down to room temperature and filtered over celite. To the filtrate, ground active carbon was added, and the resultant suspension was refluxed for 20 minutes. Afterwards, the active carbon was then removed by filtration, and the filtrate was washed with 1 M HCl and brine. The organic phase was then dried over sodium sulfate, concentrated under reduced pressure, and the residue recrystallized from PE. The product was isolated as a brownish solid (840 mg, 1.76 mmol, yield: 36 %).

^{1}H NMR (400 MHz, DMSO): δ 7.17 – 7.06 (m, 4H), 7.00 – 6.92 (m, 4H), 6.81 (s, 2H), 3.76 (s, 6H), 2.38 (s, 3H).

^{13}C NMR (101 MHz, DMSO) δ 157.1 (C_{quat}), 149.0 (C_{quat}), 139.0 (C_{quat}), 128.0, 126.5 (C_{quat}), 125.17, 120.9, 115.7, 55.7 (CH_3), 22.7 (CH_3).

GC-MS: m/z calc 477.20, found 477.0, 476.1, 478.2.

ATR-IR (v, cm^{-1}): 2932.9*w*, 2840.1*w,* 1883.8*w,* 1605.6*m,* 1587.0*s,* 1523.1*m,* 1504.6*s,* 1438.6*s,* 1327.3*m,* 1286.1*w,* 1240.8*s,* 1181*s,* 1162.44*m,* 1102.7*s,* 1030.5*s,* 1007.9*m,* 968.7*m,* 927.5*m,* 849.2*m,* 828.5*s,* 814.1*s,* 781.1*m,* 729.6*s,* 647.17*m,* 579.16*s,* 476.1*m.*

Melting point: 135 ± 4 °C

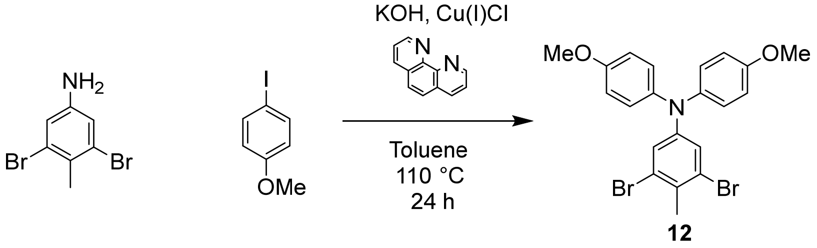

Scheme S2 Suzuki coupling starting materials preparation.

Suzuki coupling general procedure:

The corresponding bromo triarylamine derivative (1 eq), $Pd(PPh_3)_2Cl_2$ (0.05 eq), anhydrous K_2CO_3 (1eq), and corresponding pyridine boronic acid (1.15 eq) were added to an RBF under a N_2 flow. Dioxane/Water (4:1 vol/vol, to get ≈0.1 M solution) was then added, and the suspension was degassed by bubbling N_2 for 20 minutes and then heated at 90 °C for 20 h. Afterwards, the suspension was cooled down and filtered over celite. The filtrate was partitioned between DCM/H_2O, and the organic phase was washed with water.

The organic phase was then dried over sodium sulfate and concentrated to dryness under reduced pressure. The residue was taken up in acidic water, and the undissolved material was filtered off. The water phase was then made alkaline (pH 14) by the careful addition of NaOH powder. The obtained suspension was then filtered on paper, and solids were washed with water, collected with Acetone, and concentrated under reduced pressure. The products were used without further processing.

*4-methoxy-N-(4-methoxyphenyl)-N-(4-(pyridin-4-yl)phenyl)aniline (**13**)*:

4-Bromo-4',4''-dimethoxytriphenylamine (BLDpharm) and pyridin-4-yl boronic acid were used. 55 % yield. Spectroscopic data are consistent with the literature.[11]

*4-methoxy-N-(4-methoxyphenyl)-N-(4-(pyridin-3-yl)phenyl)aniline (**14**):*

4-Bromo-4',4''-dimethoxytriphenylamine (BLDpharm) and pyridin-3-yl boronic acid were used. The crude product was isolated as a yellow solid. Yield: 90.6 % (590 mg, 1.54 mmol). ^{1}H NMR (400 MHz, DMSO) δ 9.07 – 8.80 (m, 1H), 8.63 – 8.42 (m, 1H), 8.10 – 7.96 (m, 1H), 7.65 – 7.56 (m, 2H), 7.16 – 7.10 (m, 4H), 7.04 – 6.97 (m, 5H), 6.94 – 6.83 (m, 2H), 3.81 (s, 6H).

*3,5-di-tert-butyl-4-methoxy-N,N-bis(4-(pyridin-4-yl)phenyl)aniline (**15**):* N,N-bis(4-bromophenyl)-3,5-di-tert-butyl-4-methoxyaniline (***11***) and pyridin-4-yl boronic were used. The crude product was isolated as a yellow solid. Yield 79 % (1.180 g, 2.178 mmol).

^{1}H NMR (400 MHz, DMSO) δ 8.65 – 8.46 (m, 5H), 7.85 – 7.73 (m, 5H), 7.73 – 7.60 (m, 5H), 7.16 – 7.09 (m, 4H), 7.06 (s, 2H), 3.69 (s, 3H), 1.34 (s, 18H).

*N,N-bis(4-methoxyphenyl)-4-methyl-3,5-di(pyridin-4-yl)aniline (**16**):*

3,5-dibromo-N,N-bis(4 methoxyphenyl)-4-methylaniline (***12***) and pyridin-4-yl boronic were used. The crude product was isolated as a yellow solid. Yield: 77.2 % (465 mg, 0.982 mmol). The product was isolated as an off-white solid.

^{1}H NMR (400 MHz, DMSO) δ 8.59 (s, 4H), 7.38 – 7.31 (m, 4H), 7.13 – 7.04 (m, 5H), 6.97 – 6.86 (m, 5H), 6.68 – 6.62 (m, 2H), 3.72 (s, 6H), 1.97 (s, 3H).

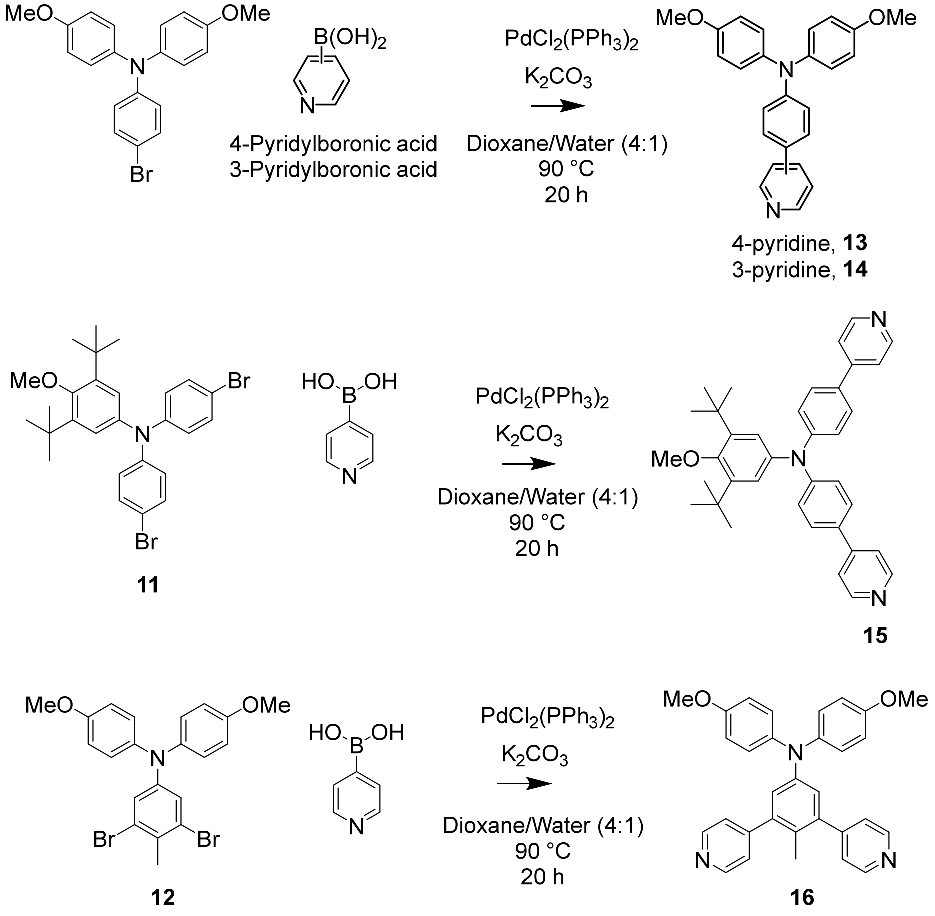

Scheme S3 Suzuki coupling reactions.

TAA-pyridine quaternization procedures:

*4-(4-(bis(4-methoxyphenyl)amino)phenyl)-1-methylpyridin-1-ium iodide (**1**)*: Compound 13, 4-methoxy-N-(4-methoxyphenyl)-N-(4-(pyridin-4-yl)phenyl)aniline (1 eq, 600 mg, 1.57 mmol) was dissolved in DCM and added to a pressure flask. MeI (2.05 eq, 0.2 mL, 3.21 mmol) was then added, and the solution was stirred for 24 h at 50 °C. Afterwards, the solution was poured into diethyl ether and a red solid crushed out. The solid was filtered off and washed with diethyl ether and ethyl acetate. The product was isolated as a red solid (680 mg, 1.30 mmol, 82 % yield).

^{1}H NMR (400 MHz, DMSO) δ 8.92 – 8.87 (m, 2H), 8.34 – 8.30 (m, 2H), 7.97 (dd, 0H), 7.20 – 7.15 (m, 4H), 7.03 – 6.99 (m, 3H), 6.79 – 6.69 (m, 2H), 4.28 (s, 3H), 3.77 (s, 6H).

^{13}C NMR (151 MHz, DMSO) δ 157.5 (C_{quat}), 153.7 (C_{quat}), 152.6 (C_{quat}), 145.4 (CH), 138.7 (C_{quat}), 130.1 (CH), 128.5 (CH), 122.8 (C_{quat}), 117.2 (CH), 115.7 (CH), 55.8 (OCH_3), 46.8 (N^+-CH_3).

^{15}N-HMBC (61 MHz, DMSO): -190.20 (N-pyridinium), -279.35 (N-triphenylamine)

HRESI-MS: m/z calc. 397.1910, found 397.1911

ATR-IR (ν, cm^{-1}): 3421*m*, 3025*m*, 1638*m*, 1584*s*, 1488.1*s*, 1333.5*m*, 1294.3*s*, 1226.3*s*, 1185.1*s*, 1164.5*s*, 1106.8*m*, 1024.3*m*, 818.2*s*, 711.1*m*, *577.1s*, 494.6*m*, 406.0*s*.

Melting point: 118 ± 3 °C

*3-(4-(bis(4-methoxyphenyl)amino)phenyl)-1-methylpyridin-1-ium iodide (**2**)*: see the procedure for (**1**). Compound ***14*** was used as the starting material instead of ***13***. The product was isolated as an intense orange solid (490 mg, 0.934 mmol, 65%).

^1H NMR (600 MHz, DMSO) δ 9.50 (s, 1H), 8.93 (d, J = 6.0 Hz, 1H), 8.81 – 8.77 (m, 1H), 8.12 (dd, J = 8.3, 5.9 Hz, 1H), 7.80 – 7.76 (m, 2H), 7.16 – 7.09 (m, 4H), 7.02 – 6.95 (m, 5H), 6.86 – 6.82 (m, 2H), 4.45 (s, 3H), 3.77 (s, 6H).

^{13}C NMR (151 MHz, DMSO) δ 157.00(C_{quat}), 150.7 (C_{quat}), 143.0, 142.8, 140.8, 139.5 (C_{quat}), 139.4, 128.7, 128.1, 123.6 (C_{quat}), 118.5, 115.9, 115.7 (CH), 55.8 (OCH_3), 48.4 (N^+-CH_3).

^{15}N NMR (61 MHz, DMSO) δ -178.26 (N-pyridinium), -284.25 (N-triarylamine).

HRESI-MS: m/z calc. 397.1911, found 397.1904.

ATR-IR (ν, cm^{-1}): 3338.9*w*, 3033.9*w*, 2833.9*w*, 1601.4*m*, 1494.3*s*, 1104.7*m*, 1026.4*s*, 913.0*w*, 828.4*s*, 781.1*m*, 676.03*m*, 579.2*s*, 525.6*s*, 420.5*w*.

Melting point: 100 ± 3 °C (Chloride salt)

*4,4'-(((3,5-di-tert-butyl-4-methoxyphenyl)azanediyl)bis(4,1-phenylene))bis(1-methylpyridin-1-ium) diodide (**3**):* Compound ***15***, 3,5-di-tert-butyl-4-methoxy-N,N-bis(4-(pyridin-4-yl)phenyl)aniline (1 eq, 1.167 g, 2.154 mmol), was dissolved in THF and added to a pressure flask. An excess of MeI (6eq, 0.8 mL, 12.93 mmol) was added, and the mixture was stirred at 60 °C for 24 h. The product was then filtered off and washed with diethyl ether. The product was isolated as a reddish solid (1.43 g, 1.73 mmol, 80 %).

^1H NMR (600 MHz, MeOD) δ 8.82 (d, J = 6.5 Hz, 4H), 8.36 (d, J = 6.4 Hz, 4H), 8.06 – 8.01 (m, 4H), 7.32 – 7.27 (m, 4H), 7.15 (s, 2H), 4.38 (s, 2H), 3.77 (s, 1H), 1.39 (s, 18H).

^{13}C NMR (151 MHz, MeOD) δ 158.0 (C_{quat}), 154.8 (C_{quat}), 150.6 (C_{quat}), 145.7 (C_{quat}), 144.9, 139.9 (C_{quat}), 129.2, 127.1 (C_{quat}), 125.4, 123.0, 122.8, 63.9 (OCH_3), 46.3 (N^+-CH_3), 31.0 ($(CH_3)_3$).

^{15}N NMR (61 MHz, MeOD) δ -185.24 (N-pyridinium), -268.44 (N-triarylamine)

HRESI-MS: m/z calc. 285.6776, found 285.6778.

ATR-IR (v, cm^{-1}): 3353.3*w*, 2953.3*m,* 1638.5*m,* 1580.8*s,* 1490.1*s,* 1407.7*s,* 1290.2*s,* 1183.0*s,* 1113.0*s,* 997.5*m,* 818.2*s,* 715.2*m,* 630.7*m,* 502.9*s,* 406.0*s.*
Melting point: 127 ± 4 °C (Chloride salt)

*4,4'-(5-(bis(4-methoxyphenyl)amino)-2-methyl-1,3-phenylene)bis(1-methylpyridin-1-ium) diiodide (**4**):* Compound ***16***, N,N-bis(4-methoxyphenyl)-4-methyl-3,5-di(pyridin-4-yl)aniline (1 eq, 220 mg, 0.465 mmol), was dissolved in DCM and added to a pressure flask. MeI (4.15 eq, 0.12 mL, 1.93 mmol) was then added, and the mixture was stirred for 20 h at 50 °C. Afterwards, the suspension was then diluted with DCM, filtered, and the solid was washed with diethyl ether. The product was isolated as a solid (233 mg, 0.308 mmol, 66 %).
^{1}H NMR (600 MHz, MeOD) δ 8.98 – 8.93 (m, 4H), 8.12 – 8.08 (m, 4H), 7.17 – 7.11 (m, 4H), 6.97 – 6.88 (m, 6H), 4.46 (s, 6H), 3.78 (s, 6H), 2.19 (s, 3H).
^{13}C NMR (151 MHz, MeOD) δ 158.1 (C_{quat}), 157.1 (C_{quat}), 147.9 (C_{quat}), 145.2, 139.4 (C_{quat}), 138.5 (C_{quat}), 128.1, 127.1, 122.7 (C_{quat}), 120.7, 114.9, 54.6 ($OC\mathit{H}_3$), 47.2 (N^+-$C\mathit{H}_3$), 16.3 (CH_3)
^{15}N NMR (61 MHz, MeOD) δ -180.09 (N-pyridinium), -285.09 (N-Triarylamine)
HRESI-MS: m/z calc. 251.6281, found 251.6288.
ATR-IR (v, cm^{-1}): 3353.3*m,* 3017.4*w,* 2836.0*w,* 1640.6*m,* 1595.3*m,* 1562.3*m,* 1502.5*s,* 1358.2*m,* 1277.9*m,* 1232.5*s,* 1024.3*s,* 838.8*s,* 783.2*m,* 746.1*m,* 731.7*m,* 717.2*m,* 622.4*s,* 599.8*s,* 568.8*s,* 418.4*s.*
Melting point: 304 ± 10 °C (Chloride salt).

*4,4'-(5-(bis(4-methoxyphenyl)amino)-2-methyl-1,3-phenylene)bis(1-(3-((2-hydroxyethyl)dimethyl ammonio)propyl)pyridin-1-ium) tetrabromide (**5**)*: Compound ***16***, N,N-bis(4-methoxyphenyl)-4-methyl-3,5-di(pyridin-4-yl)aniline (1 eq, 390 mg, 0.82 mmol), was dissolved in DMF (0.2 M). 3-bromo-N-(2-hydroxyethyl)-N,N-dimethylpropan-1-aminium bromide (2.30 eq, 590 mg, 1.89 mmol) was then added and the mixture stirred at 115°C for 24 h. The obtained suspension was then filtered, and the solid was washed with MeCN and Et_2O. The product was isolated as a light yellow solid (389 mg, 0.37 mmol, 44 %).

^{1}H NMR (600 MHz, MeOD) δ 9.17 – 9.13 (m, 4H), 8.19 – 8.14 (m, 4H), 7.17 – 7.12 (m, 4H), 6.98 (s, 2H), 6.95 – 6.89 (m, 4H), 4.82 (t, J = 7.6 Hz, 4H), 4.59 (s, 2H), 4.05 – 4.01 (m, 4H), 3.79 (s, 6H), 3.73 – 3.68 (m, 4H), 3.62 – 3.57 (m, 4H), 3.27 (s, 12H), 2.72 – 2.63 (m, 4H), 2.22 (s, 3H).

^{13}C NMR (151 MHz, MeOD) δ 158.9 (C_{quat}), 157.1 (C_{quat}), 147.9 (C_{quat}), 144.7, 139.4 (C_{quat}), 138.5 (C_{quat}), 128.6, 127.1, 122.9 (C_{quat}), 120.8, 114.9, 65.5 (CH_2), 61.2 (CH_2), 57.6 (CH_2), 55.5 (CH_2), 54.6 (CH_3), 51.3 (CH_3), 24.6 (CH_2), 16.5 (CH_3).

^{15}N NMR (61 MHz, MeOD) δ -171.37 (N-pyridinium), -284.29 (N-Triarylamine), -324.91 (N-aliphatic ammonium)

ATR-IR (v, cm^{-1}): 3301.8*m,* 2996.8*m,* 2836.0*w,* 1634.4*s,* 1595.3*m,* 1560.2*w,* 1504.6*s,* 1440.7*s,* 1360.3*m,* 1230.4*s,* 1193.3*m,* 1016.1*s,* 946.0*m,* 834.7*s,* 775.0*m,* 731.7*m,* 616.3*s,* 579.2*s,* 564.7*s,* 523.5*s.*

HRESI-MS: m/z calc. 183.8675, found 183.8675

Melting point: 98 ± 3 °C (Chloride salt)

*4-(bis(4-methoxyphenyl)amino)-1-methylpyridin-1-ium iodide (**6**)*: (BMP)PA, N,N-bis(4-methoxyphenyl)pyridin-4-amine (1 eq, 305 mg, 0.99 mmol) was dissolved in DCM (0.2 M) and added to a pressure flask. MeI (2 eq, 0.12 mL, 1.8 mmol) was then added and the mixture stirred at 55°C for 24 h. Afterwards, the solution was then poured in Et_2O. The suspension was sonicated for 5 minutes and filtered off. The product was then washed with Et_2O and recovered with MeOH. A yellowish foam was collected after drying under a high vacuum (420 mg, 0.93 mmol, 94 %).

^{1}H NMR (600 MHz, MeOD) δ 8.17 – 8.12 (m, 2H), 7.39 – 7.34 (m, 4H), 7.12 – 7.06 (m, 4H), 6.81 – 6.76 (m, 2H), 4.01 (s, 3H), 3.85 (s, 6H).

^{13}C NMR (151 MHz, MeOD) δ 159.7 (C_{quat}), 157.8 (C_{quat}), 143.6, 135.0 (C_{quat}), 128.0, 115.5, 109.9, 54.8 (CH_3), 44.1 (CH_3).

^{15}N NMR (61 MHz, MeOD) δ -212.97 (N-pyridinium), -254.58 (N-triarylamine).

ATR-IR (v, cm^{-1}): 3376.0*m*, 3021.5*w,* 2838.0*w,* 1648.8*s,* 1603.5*m,* 1523.1*m,* 1504.6*s,* 1380.9*m,* 1240.8*s,* 1203.7*s,* 1024.3*s,* 832.7*s,* 783.2*m,* 731.7*m,* 579.2*s,* 480.2*m.*

GC-MS: m/z calc 321.1597, found 321.1.

Melting point: 120 ± 4 °C (Chloride salt).

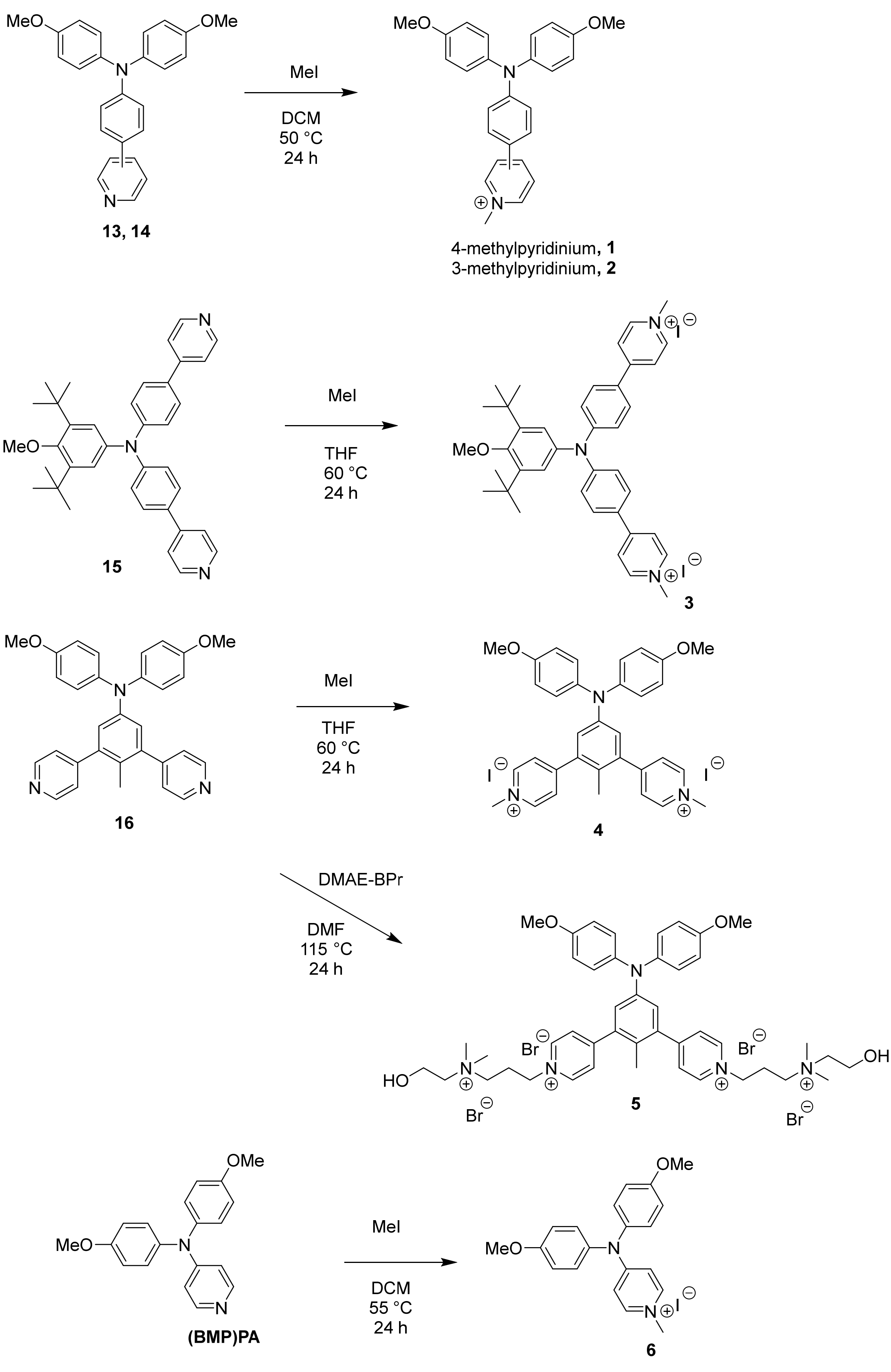

Scheme S4 Pyridines quaternization reactions.

Ullmann-type coupling general procedure for the synthesis of TAA

A literature procedure was adapted.[12] As an example, the preparation of 4-methoxy-N-(4-methoxyphenyl)-N-(4-nitrophenyl)aniline (***17***) is described: 4-nitroaniline (1 eq, 4.45 g, 32 mmol), 4-iodoanisole (2.05 eq, 15.5 g, 66 mmol), copper (2.05 eq, 4.20 g, 66 mmol), anhydrous K_2CO_3 (3 eq, 13.4 g, 96.6 mmol), KI (a pinch), 18-crown-6 (a pinch) were suspended in o-DCB (80 mL). The suspension was degassed by bubbling N_2 for 20 minutes and afterwards refluxed for 72 h. Afterwards, the suspension was cooled, filtered over celite and the solids were washed with DCM. The organic phase was then concentrated under reduced pressure (removing as much o-DCB as possible) and the oily residue recrystallized from EtOH. The product was isolated as red crystals (5.95 g, 16.98 mmol, 55 %). Spectroscopic data in agreement with a previous report.[12]

*2-methoxy-N,N-bis(4-methoxyphenyl)-4-nitroaniline (**18**):* 2-methoxy-4-nitroaniline and 4-iodoanisole were used. Red solid. Yield: 72 % (5.00 g, 13mmol).

^{1}H NMR (400 MHz, CD_2Cl_2) δ 7.76 – 7.69 (m, 2H), 6.97 – 6.88 (m, 1H), 6.91 – 6.84 (m, 4H), 6.84 – 6.75 (m, 4H), 3.77 (s, 6H), 3.64 (s, 3H).

^{13}C NMR (101 MHz, CD_2Cl_2) δ 156.2 (C_{quat}), 152.5 (C_{quat}), 143.7 (C_{quat}), 142.7 (C_{quat}), 140.8 (C_{quat}), 125.1, 124.3, 117.4, 114.3, 108.3, 56.0 (CH_3), 55.4 (CH_3).

GC-MS: m/z calc. 380.1372, found 380.3.

ATR-IR (v, cm^{-1}): 1579*w*, 1495*m*, 1463*m*, 1312*m*, 1280*m*, 1182*m*, 1166*m*, 1093*m*, 1024*m*, 831*m*, 816*m*, 556*w*.

Melting point: 107 ± 3 °C

*N-(2,4-dimethoxyphenyl)-2,4-dimethoxy-N-(4-nitrophenyl)aniline (**19**):* 4-nitroaniline and 2,4-dimethoxyiodobenzene were used. Brown solid. Yield: 68 %.

^{1}H NMR (400 MHz, CD_2Cl_2) δ 7.89 – 7.81 (m, 2H), 7.18 (d, J = 8.6 Hz, 2H), 6.50 (d, J = 2.7 Hz, 2H), 6.41 (dd, J = 8.7, 2.7 Hz, 2H), 6.32 – 6.22 (m, 2H), 3.73 (s, 6H), 3.70 (s, 6H).

^{13}C NMR (101 MHz, CD_2Cl_2) δ 160.1 (C_{quat}), 156.5 (C_{quat}), 154.7 (C_{quat}), 137.9 (C_{quat}), 130.3, 125.9 (C_{quat}), 125.3, 112.4, 105.3, 99.7, 55.6 (CH_3), 55.5 (CH_3).

GC-MS: m/z calc 410.1477, found 410.0.

ATR-IR (v, cm^{-1}): 2937.0*w*, 2836.0*w,* 1585.0*s,* 1504.6*s,* 1494.3*s,* 1438.6*m,* 1315.0*s,* 1292.3*s,* 1275.8*s,* 1238.7*s,* 1205.7*s,* 1183.0*s,* 1158.32*s,* 1106.8*s,* 1026.4*s,* 931.6*m,* 830.6*s,* 752.3*s,* 694.6*m,* 634.8*m,* 566.8*m,* 492.6*m.*

Melting point: 173 ± 5 °C.

Nitro group(s) reduction general procedure

All the nitro compounds were reduced by improving a literature procedure.[13] In a typical procedure, the corresponding nitro compound was dissolved in a mixture of MeCN/EtOH (1.2:1.0 v/v, ≈ 0.25 M). Afterwards, an excess of $SnCl_2$*$2H_2O$ (6 eq per nitro group) was added and the mixture was refluxed for 20 - 22h. The solution was then cooled down and poured into an excess of 5 M NaOH solution and the resulting white suspension was then filtered over celite (the solids were washed with a bit of MeCN). This step was found to make further processing easier by removing all the inorganics. The filtered solution was then extracted with EtOAc. The organic phase was then washed with water and brine, dried over sodium sulfate and concentrated under reduced pressure to yield the corresponding amine. The amines were used without further processing.

*N1, N1-bis(4-methoxyphenyl)benzene-1,4-diamine (**20**)*: Off-white powder. Yield: 86 % (2.03 g, 6.36 mmol). Spectroscopic data is consistent with previous reports.[13]

*2-methoxy-N1,N1-bis(4-methoxyphenyl)benzene-1,4-diamine (**21**)*: Gray solid. Yield: 88 % (3.7 g, 10.6 mmol).

^{1}H NMR (400 MHz, CD_2Cl_2) δ 6.91 – 6.79 (m, 4H), 6.78 – 6.66 (m, 4H), 6.33 – 6.22 (m, 2H), 3.73 (s, 6H), 3.64 (s, 3H).

^{13}C NMR (101 MHz, CD_2Cl_2) δ 157.2 (C_{quat}), 154.0 (C_{quat}), 146.2 (C_{quat}), 142.2 (C_{quat}), 131.1, 126.6 (C_{quat}), 121.8, 114.1, 107.5, 100.2, 55.6 (O*C*H_3), 55.4 (O*C*H_3).

ATR-IR (v, cm^{-1}):): 3450.2*w*, 3366.8*w,* 2932.9*w,* 2832.0*w,* 1496.3*s*, 1463.3*s*, 1438.6*m*, 1428.3*m,* 1327.3*m,* 1310.8*m,* 1267.5*m*, 1232.5*m*, 1203.7*s*, 1177.0*s*, 1166.5*s*, 1125.3*m*, 1104.7*m*, 1030.5*m*, 820.3*s*, 795.6*m,* 727.6*m,* 645.1*m,* 585.3*s,* 566.8*s*, 527.6*s*, 408.0*m*.

Melting point: 115 ± 3 °C

GC-MS: m/z calc. 350.16, found 350.3 (found).

*N1, N1-bis(2,4-dimethoxyphenyl)benzene-1,4-diamine (**22**)*: Dark solid. Yield: 75 % (1.5 g, 3.8 mmol).

^{1}H NMR (400 MHz, CD_2Cl_2) δ 7.03 (d, J = 8.6 Hz, 2H), 6.50 (d, J = 2.8 Hz, 2H), 6.49 – 6.36 (m, 4H), 6.38 – 6.29 (m, 2H), 3.77 (s, 6H), 3.68 (s, 6H).

^{13}C NMR (101 MHz, CD_2Cl_2) δ 158.0 (C_{quat}), 156.4 (C_{quat}), 141.9 (C_{quat}), 138.6 (C_{quat}), 130.5, 129.6 (C_{quat}), 129.1, 127.9, 120.1, 117.4, 115.6, 114.9, 104.7, 100.2, 100.0, 55.7 (OCH_3), 55.4 (OCH_3).

GC-MS: m/z calc. 308.1736, found 308.1

ATR-IR (ν, cm⁻¹): 3460.5*w*, 3378.1*w*, 2932.9*w*, 2836.8*w*, 1580.8*m*, 1500.5*s*, 1436.6*s*, 1415.9*m*, 1269.6*s*, 1205.7*s*, 1154.2*s*, 1121*s*, 1032.6*s*, 939.8*m*, 816.2*s*, 801.7*s*, 737.9*m*, 632.8*m*, 509.1*s*.

Melting point: 125 ± 4 °C

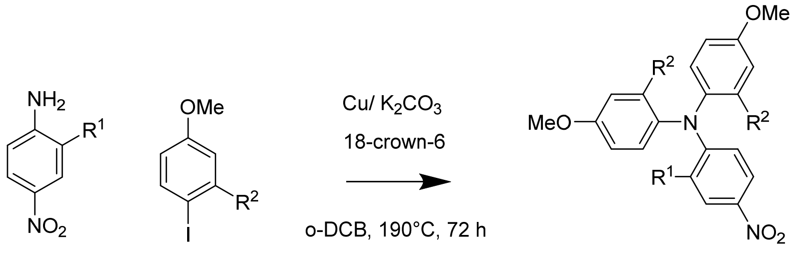

$R^1 = R^2 = H$, **17**
$R^1 = OMe$, $R^2 = H$, **18**
$R^1 = H$, $R^2 = OMe$, **19**

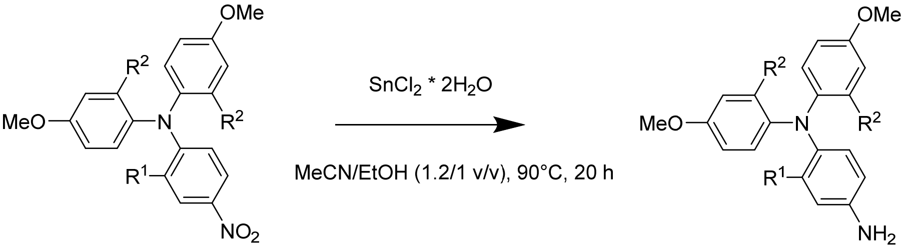

$R^1 = R^2 = H$, **20**
$R^1 = OMe$, $R^2 = H$, **21**
$R^1 = H$, $R^2 = OMe$, **22**

Scheme S5: Ullmann-type coupling and subsequent nitro reductions.

Non-symmetric Viologens synthesis (Zincke reaction precursors)

1-(2,4-dinitrophenyl)-[4,4'-bipyridin]-1-ium chloride (***23***, Zincke salt) was prepared according to a literature procedure.[14] ***23*** was then further quaternized with an excess of the selected alkylating agent (i.e., 3-bromopropanol or 3-bromo-N-(2-hydroxyethyl)-N,N-dimethylpropan-1-aminium bromide) in a MeCN:EtOH mixture (see Scheme S6).

*1-(2,4-dinitrophenyl)-1'-(3-hydroxypropyl)-[4,4'-bipyridine]-1,1'-diium bromide chloride (**24**)*: Compound *23* ,1-(2,4-dinitrophenyl)-[4,4'-bipyridin]-1-ium chloride (1 eq, 1.05 g, 2.93 mmol), was dissolved in anhydrous MeCN: EtOH (2:1 v/v, 0.3 M) in a pressure flask. 3-bromopropanol (3eq, 0.76 mL, 8.78 mmol) was then added and the mixture was heated to 95°C for 3 days. Afterwards, the obtained suspension was then filtered and the product was washed with MeCN and Et_2O. The product was isolated as a yellow solid (914 mg, 1.84 mmol, 63 %).

^{1}H NMR (400 MHz, D_2O) δ 9.46 – 9.38 (m, 3H), 9.26 – 9.19 (m, 2H), 8.96 (dd, J = 8.7, 2.5 Hz, 1H), 8.87 – 8.80 (m, 2H), 8.68 (d, J = 6.7 Hz, 2H), 8.31 (d, J = 8.8 Hz, 1H), 4.89 (t, J = 7.2 Hz, 2H), 3.74 (t, J = 5.9 Hz, 2H), 2.35 (ddd, J = 13.0, 7.1, 5.8 Hz, 2H).

^{13}C NMR (101 MHz, D_2O) δ 153.20 (C_{quat}), 149.81 (C_{quat}), 149.57 (C_{quat}), 146.62, 145.94, 142.84 (C_{quat}), 138.25 (C_{quat}), 131.21, 130.76, 127.40, 127.26, 122.81, 59.67 (CH_3), 57.84 (CH_3), 32.68 (CH_2).

ATR-IR (ν, cm^{-1}): 3334.80*m*, 3114.26*w*, 2976.17*m*, 1634.42 *m*, 1607.63 *m*, 1527.25 *s*, 1446.86 *m*, 1339.69 *s*, 1240.76 *m*, 1224.27 *s*, 1077.93 *m*, 1053.20*m*, 1038.77*m*, 917.17*m*, 906.87*m*, 859.46*m*, 834.73*s*, 828.55*s*, 762.59*m*, 748.16*m*, 737.86 *m*, 729.62*m*, 715.19*s*, 690.46 *m*, 535.88*s*, 465.80*s*, 447.25*m*

GC-MS: m/z calc. 191.063, 183.9, 156.00 (4,4-bipyridine), 108.9 (M^+ + H^+ - dinitrobenzene) [Exact m/z could not be found due to fragmentation that can be usually observed in viologens]

Melting point: 240 ± 7 °C

*1-(2,4-dinitrophenyl)-1'-(3-((2-hydroxyethyl)dimethylammonio)propyl)-[4,4'-bipyridine]-1,1'-diium di bromide chloride (**25**)*: Compound 23, 1-(2,4-dinitrophenyl)-[4,4'-bipyridin]-1-ium chloride (1 eq, 1.00 g, 2.78 mmol), was dissolved in MeCN: EtOH (2:3 v/v, 0.28 M) in a pressure flask. 3-bromo-N-(2-hydroxyethyl)-N,N-dimethylpropan-1-aminium bromide (3.0 eq, 2.43 g, 8.36 mmol) was then added and the mixture was heated to 95°C for 72 h. The obtained suspension was then filtered and the solid was washed with MeCN and Et_2O. A yellow solid is collected (930 mg, 1.43 mmol, 51 %).

^1H NMR (400 MHz, D_2O) δ 9.51 – 9.41 (m, 3H), 9.34 – 9.28 (m, 2H), 8.99 (dd, J = 8.7, 2.5 Hz, 1H), 8.92 – 8.86 (m, 2H), 8.80 – 8.74 (m, 2H), 8.36 (d, J = 8.8 Hz, 1H), 4.94 (t, J = 7.5 Hz, 2H), 4.12 – 4.05 (m, 2H), 3.75 – 3.66 (m, 2H), 3.65 – 3.58 (m, 2H), 3.27 (s, 7H), 2.76 (dq, J = 10.6, 7.8 Hz, 2H).

^{13}C NMR (101 MHz, D_2O) δ 152.99 (C_{quat}), 150.19 (C_{quat}), 149.82 (C_{quat}), 146.66 (CH), 145.96 (CH), 142.83 (C_{quat}), 138.24 (C_{quat}), 131.21 (CH), 130.77 (CH), 127.78 (CH), 127.34 (CH), 122.81 (CH), 65.29 (CH_2), 61.06 (CH_2), 58.47 (CH_2), 55.38 (CH_2), 51.84 (CH_3), 24.42 (CH_2).

GC-MS: m/z calc. 151.4025, found 151.1, 156.1 (4,4-bpy), 108.8 (M^+ + H^+ - dinitrobenzene)

ATR-IR (ν, cm^{-1}): 3363*m,* 3110*m*, 3005*m*, 1634*s*, 1609.69*s*, 1537.55*s*, 1444.80 *s*, 1341.75*s*, 1226.33*m*, 1077.93*m*, 917.17*m*, 834.73*s*, 715.19*m*, 507.02*s.*

Melting point: 250 ± 8 °C (Decomposition)

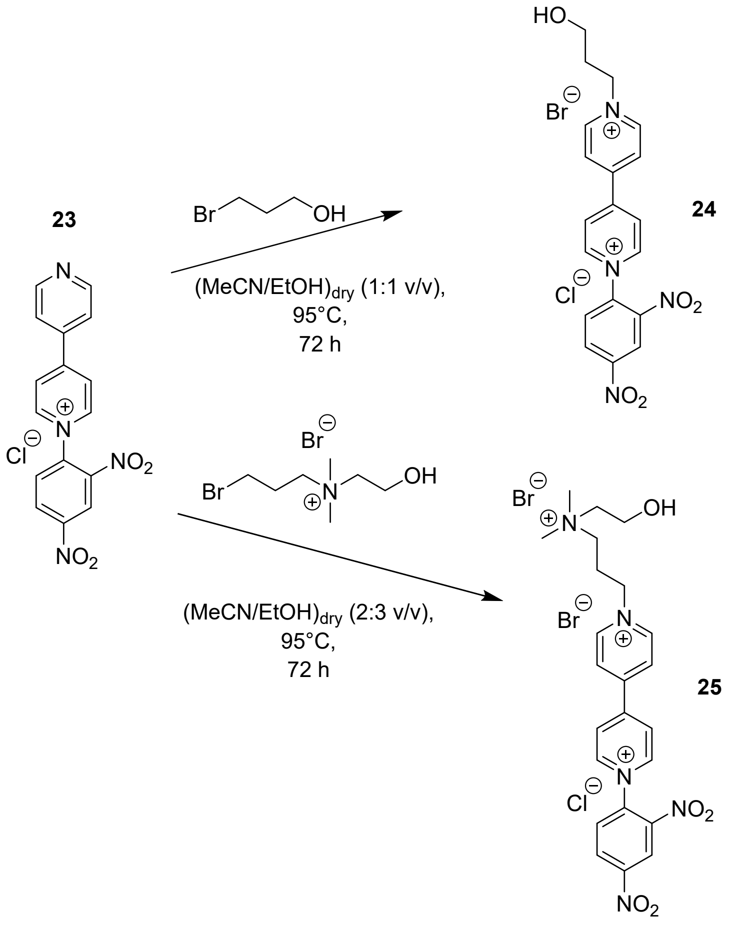

Scheme S6: Non-symmetric viologens synthesis (Zincke reaction precursors).

Zincke reaction

The Zincke reaction was performed either under MWs (by adapting a literature protocol)[15] or under conventional heating in a pressure flask (see Scheme 7).

Microwave procedure: The corresponding viologen (1 eq) and aniline (3 to 3.5 eq) were added to a 35 mL MW tube. The solvent mixture H_2O:EtOH (1:1 v/v, ≈0.1 M) was then added and the tube was sealed with a PTFE cap. The reaction was then carried out at 130 °C for 45 minutes at 150 watts (dynamic mode). Afterwards, the mixture was cooled down, diluted with water and filtered over celite. The obtained solution was then concentrated to dryness under reduced pressure and the residue was dissolved in MeOH or i-PrOH. An excess of Et_2O was added and the resulting suspension filtered off and the product collected.

Note: The thermal procedure gave higher purity products. Longer reaction times gave lower purity in the final product, and thus it is speculated that shorter reaction times will work better. However, the reaction time was not optimized.

Thermal procedure: The corresponding viologen (1 eq) and aniline (2.0 eq) were added to a pressure flask. The solvent mixture H_2O:EtOH (2:1 v/v, ≈0.15 to 0.2 M) was then added and the reaction was then carried out at 105 °C for 24 h. Afterwards, the mixture was cooled down and diluted with water and filtered over celite. The obtained solution was then concentrated to dryness under reduced pressure and the residue was dissolved in MeOH. The obtained solution in MeOH was then poured into an excess of Et_2O and the resulting suspension filtered off and the product collected.

*1-(4-(bis(4-methoxyphenyl)amino)phenyl)-1'-(3-hydroxypropyl)-[4,4'-bipyridine]-1,1'-diium, bromide chloride (***7***)*: MW procedure (45 min), 48 % yield (190 mg).

1H NMR (600 MHz, MeOD) δ 9.43 (d, J = 6.5 Hz, 2H), 9.35 (d, J = 6.4 Hz, 2H), 8.80 (dd, J = 12.3, 6.3 Hz, 3H), 7.73 – 7.68 (m, 2H), 7.22 – 7.15 (m, 4H), 7.05 – 6.95 (m, 6H), 4.94 (t, J = 7.0 Hz, 2H), 3.82 (d, J = 1.7 Hz, 7H), 3.73 (t, J = 5.7 Hz, 2H), 2.38 – 2.31 (m, 2H).

^{13}C NMR (151 MHz, MeOD) δ 157.7 (C_{quat}), 157.6 (C_{quat}), 152.1 (C_{quat}), 149.7 (C_{quat}), 148.9, 146.1, 144.4, 138.9, 138.7 (C_{quat}), 133.2 (C_{quat}), 127.8, 127.7, 126.8, 126.7, 124.6, 117.8, 115.0, 59.6 (CH_2), 57.6 (CH_2), 54.7 (O*C*H_3), 33.1 (CH_2).

^{15}N NMR (61 MHz, MeOD) δ -158.49 (N-pyridinium – aromatic linker), -162.45 (N-pyridinium),-278.35 (N-triarylamine).

HRESI-MS: m/z calc 259.6256, found 259.6261

ATR-IR (v, cm^{-1}): 3332.73*m*, 3021.51*m*, 2833.96*m*, 1632.36*m*, 1584.96*m*, 1494.27*s*, 1325.26*m*, 1234.58*s*, 1193.35*s*, 1106.79*m*, 1026.41*s*, 822.36*s*, 781.14*m*, 715.19*m*, 523.51*s*, 412.21*s*.

Melting point: 217 ± 7 °C (Chloride salt)

*1-(4-(bis(4-methoxyphenyl)amino)phenyl)-1'-(3-((2-hydroxyethyl)dimethylammonio)propyl)-[4,4'-bipyridine]-1,1'-diium dibromide chloride (**8**):* MW procedure (50 min), 36 % yield (120 mg).

^{1}H NMR (600 MHz, MeOD) δ 9.46 – 9.42 (m, 4H), 8.82 (d, J = 6.3 Hz, 4H), 7.74 – 7.68 (m, 2H), 7.23 – 7.17 (m, 4H), 7.05 – 6.97 (m, 6H), 4.95 (t, J = 7.3 Hz, 2H), 4.05 – 4.01 (m, 2H), 3.83 (s, 6H), 3.77 – 3.71 (m, 2H), 3.64 – 3.60 (m, 2H), 3.29 (s, 6H), 2.75 (td, J = 9.3, 5.6 Hz, 2H).

^{13}C NMR (151 MHz, MeOD) δ 157.7 (C_{quat}), 152.1 (C_{quat}), 150.2 (C_{quat}), 148.8 (C_{quat}), 146.1, 144.4, 138.7 (C_{quat}), 133.2 (C_{quat}), 127.8, 127.76, 127.04, 126.8, 124.6, 117.8, 115.0, 65.4 (CH_2), 61.1 (CH_2), 58.2 (CH_2), 55.5 (CH_2), 54.7 (CH_3), 51.4 (CH_3), 24.5 (CH_2).

^{15}N NMR (61 MHz, MeOD) δ -158.49 (N-pyridinium – aromatic linker), -165.42 (N-pyridinium), -278.35 (N-triarylamine), -324.91 (N- aliphatic ammonium).

HRESI-MS: m/z calc. 197.1106, found 197.1110.

ATR-IR (ν, cm^{-1}): 3266.7*m*, 3035.9*m,* 2114.6*w,* 1634.4*m,* 1591.1*m,* 1496.3*s,* 1463.3*s,* 1323.2*m,* 1300.5*m,* 1290.2*m,* 1240.8*s,* 1191.3*m.* 1181.0*s,* 1108.8*m,* 1028.5*s,* 830.6*s,* 719.3*s,* 581.2*s,* 527.6*s,* 414.3*s.*

Melting point: 223 ± 7 °C (Chloride salt)

*1-(4(bis(4methoxyphenyl)amino)3methoxyphenyl)1'(3((2hydroxyethyl)dimethylammonio) propyl)-[4,4'-bipyridine]-1,1'-diium dibromide chloride (**9**)*: Deep purple solid. MW procedure (45 min): 53 % yield (200 mg). Thermal procedure: 85 % yield (1.90 g).

^{1}H NMR (600 MHz, MeOD) δ 9.55 (d, J = 6.7 Hz, 2H), 9.46 (d, J = 6.5 Hz, 2H), 8.89 – 8.83 (m, 4H), 7.64 (d, J = 2.6 Hz, 1H), 7.44 (dd, J = 8.6, 2.6 Hz, 1H), 7.23 (d, J = 8.6 Hz, 1H), 6.94 – 6.88 (m, 4H), 6.88 – 6.82 (m, 4H), 4.96 (t, J = 7.4 Hz, 2H), 4.06 – 4.01 (m, 2H), 3.79 (d, J = 2.9 Hz, 9H), 3.79 – 3.72 (m, 2H), 3.66 – 3.59 (m, 2H), 3.30 (s, 6H), 2.81 – 2.73 (m, 2H).

^{13}C NMR (151 MHz, MeOD) δ 155.92, 155.01 (C_{quat}), 150.20 (C_{quat}), 149.67 (C_{quat}), 146.14, 145.17, 141.14, 140.67 (C_{quat}), 137.88 (C_{quat}), 127.16, 127.13, 126.87, 124.30, 116.70, 114.18, 109.08, 65.41 (CH_2), 61.06 (CH_2), 58.22 (CH_2), 55.55 (CH_3), 55.47 (CH_2), 54.65 (CH_3), 51.40 (CH_3), 24.54 (CH_2).

^{15}N NMR (61 MHz, MeOD) δ -159.01 (N-pyridinium – aromatic linker), -165.94 (N-pyridinium), -290.75 (N-triarylamine), -325.42 (N- aliphatic ammonium)

HRESI-MS: calc 207.1141, found 414.9801 (2*m/z)

ATR-IR (v, cm^{-1}): 3349.2*s*, 3038.2*m,* 2836.0*w,* 2116.7*w,* 1634.4*m,* 1498.4*s,* 1315.0*m,* 1267.5*m,* 1236.6*s,* 1106.8*m,* 1030.5*s,* 1014.0*s,* 939.8*m,* 830.6*s,* 719.3*s,* 521.4*s*

Melting point: 228 ± 6 °C (Chloride salt)

*1-(4-(bis(2,4-dimethoxyphenyl)amino)phenyl)-1'-(3-((2 hydroxyethyl)dimethylammonio) propyl)-[4,4'-bipyridine]-1,1'-diium dibromide chloride (**10**):* MW procedure (60 min): 24 % yield (120 mg). Thermal procedure: 90 % yield (1.00 g).

^{1}H NMR (600 MHz, MeOD) δ 9.45 – 9.41 (m, 2H), 9.41 – 9.37 (m, 2H), 8.82 – 8.76 (m, 4H), 7.64 – 7.58 (m, 2H), 7.29 (d, J = 8.7 Hz, 2H), 6.71 (d, J = 2.7 Hz, 2H), 6.62 – 6.55 (m, 4H), 4.94 (t, J = 7.4 Hz, 2H), 4.03 (dt, J = 5.2, 2.7 Hz, 2H), 3.83 (d, J = 15.0 Hz, 11H), 3.78 – 3.71 (m, 2H), 3.64 – 3.59 (m, 2H), 3.29 (s, 6H), 2.79 – 2.71 (m, 2H).

^{13}C NMR (151 MHz, MeOD) δ 160.1 (C_{quat}), 156.7 (C_{quat}), 152.4 (C_{quat}), 150.3 (C_{quat}), 148.2 (C_{quat}), 146.1, 144.1, 131.7 (C_{quat}), 130.2, 126.9, 126.8, 126.2 (C_{quat}), 124.0, 114.2, 105.3, 99.4, 65.4 (CH_2), 61.0 (CH_2), 58.1 (CH_2), 55.5 (CH_2), 54.8 (CH_3), 54.7 (CH_3), 51.4 (CH_3), 24.5 (CH_2).

^{15}N NMR (61 MHz, MeOD) δ -157.50 (N-pyridinium – aromatic linker), -165.42 (N-pyridinium), -293.21 (N-triarylamine), -324.91 (N- aliphatic ammonium).

HRESI-MS: m/z calc 217.1177, found 217.1173.

ATR-IR (v, cm^{-1}): 3369.8*m*, 2932.9*w,* 1632.4*m,* 1585.0*m,* 1502.5*s,* 1275.8*m,* 1205.7*s,* 1156.2*s,* 1024.3*s,* 824.4*s,* 636.9*m,* 605.9*s,* 573.0*s,* 556.5*s,* 529.7*s,* 505.0*s,* 495.6*s,* 486.4*s,* 470.0*s,* 461.7*s,* 447.2*s,* 437.0*m,* 420.5*s.*

Melting point: 228 ± 6 °C (Chloride salt)

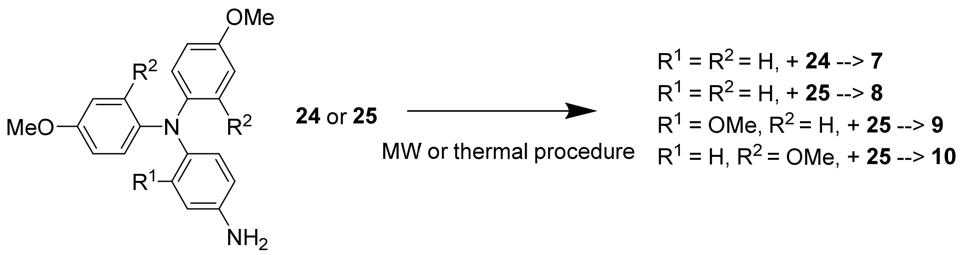

Scheme S7: Zincke reaction.

NMR spectra

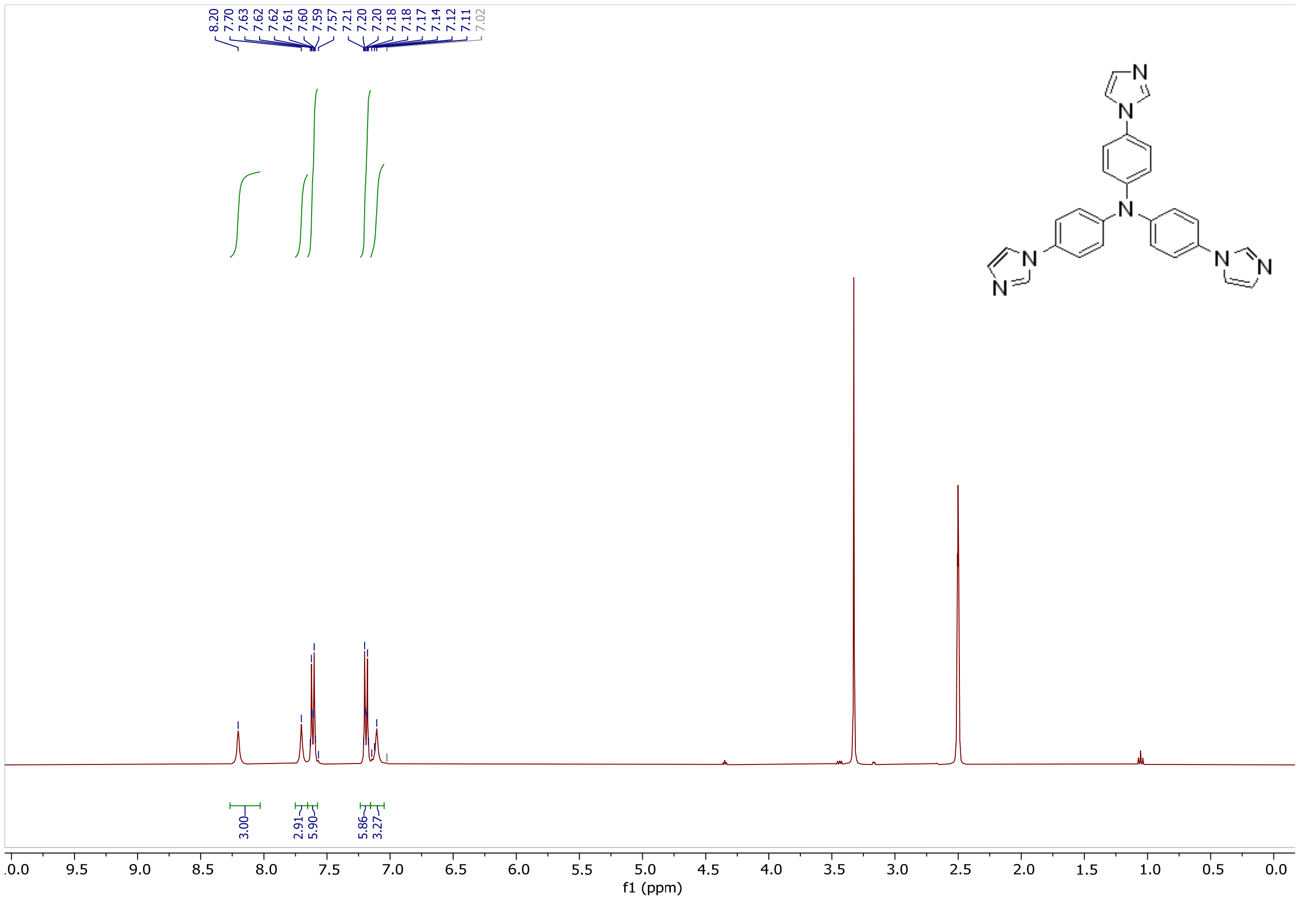

Fig. S13: TIPA ^{1}H-NMR (dmso-d_6).

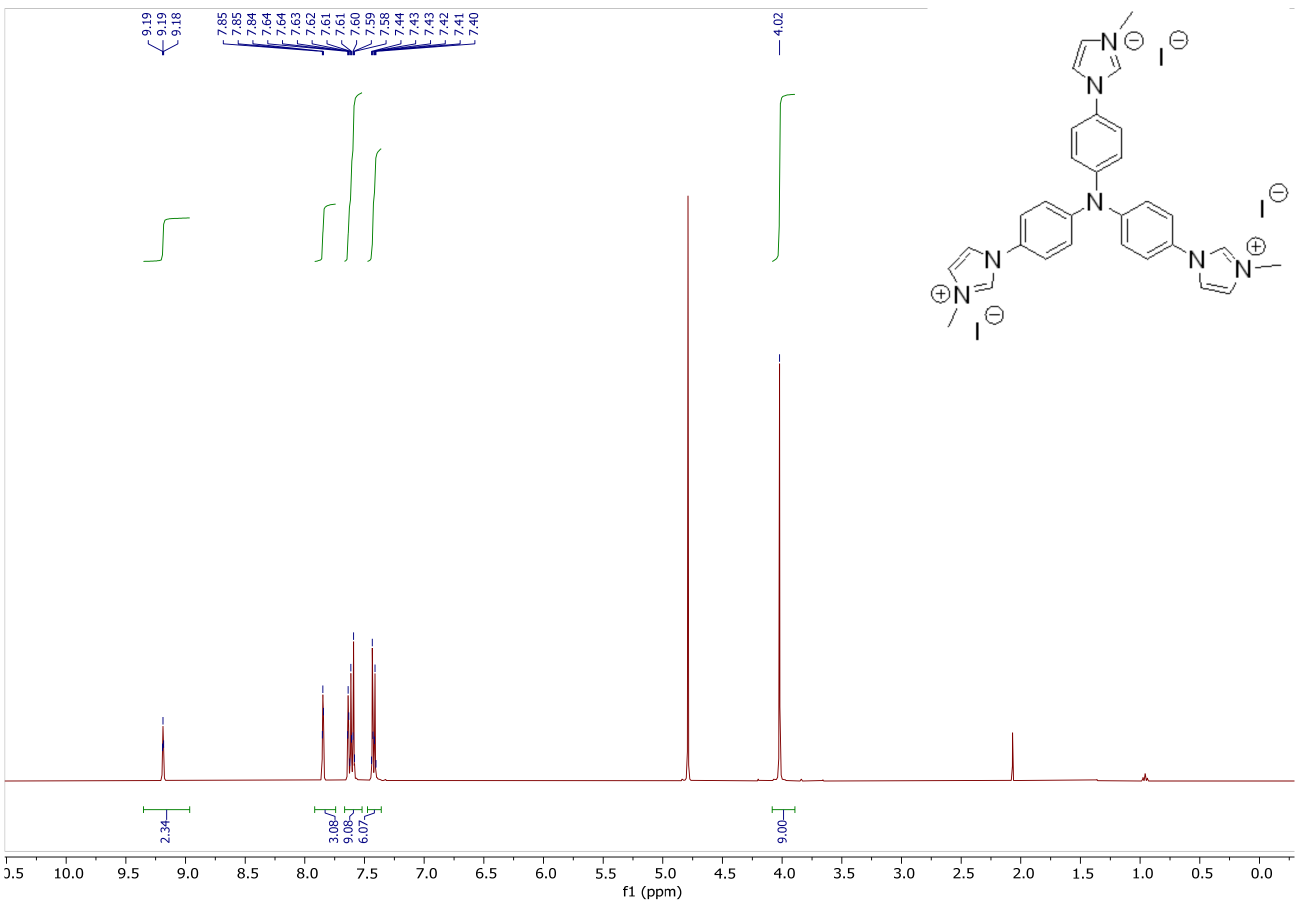

Fig. S14: TMIPA ^{1}H-NMR (D_2O).

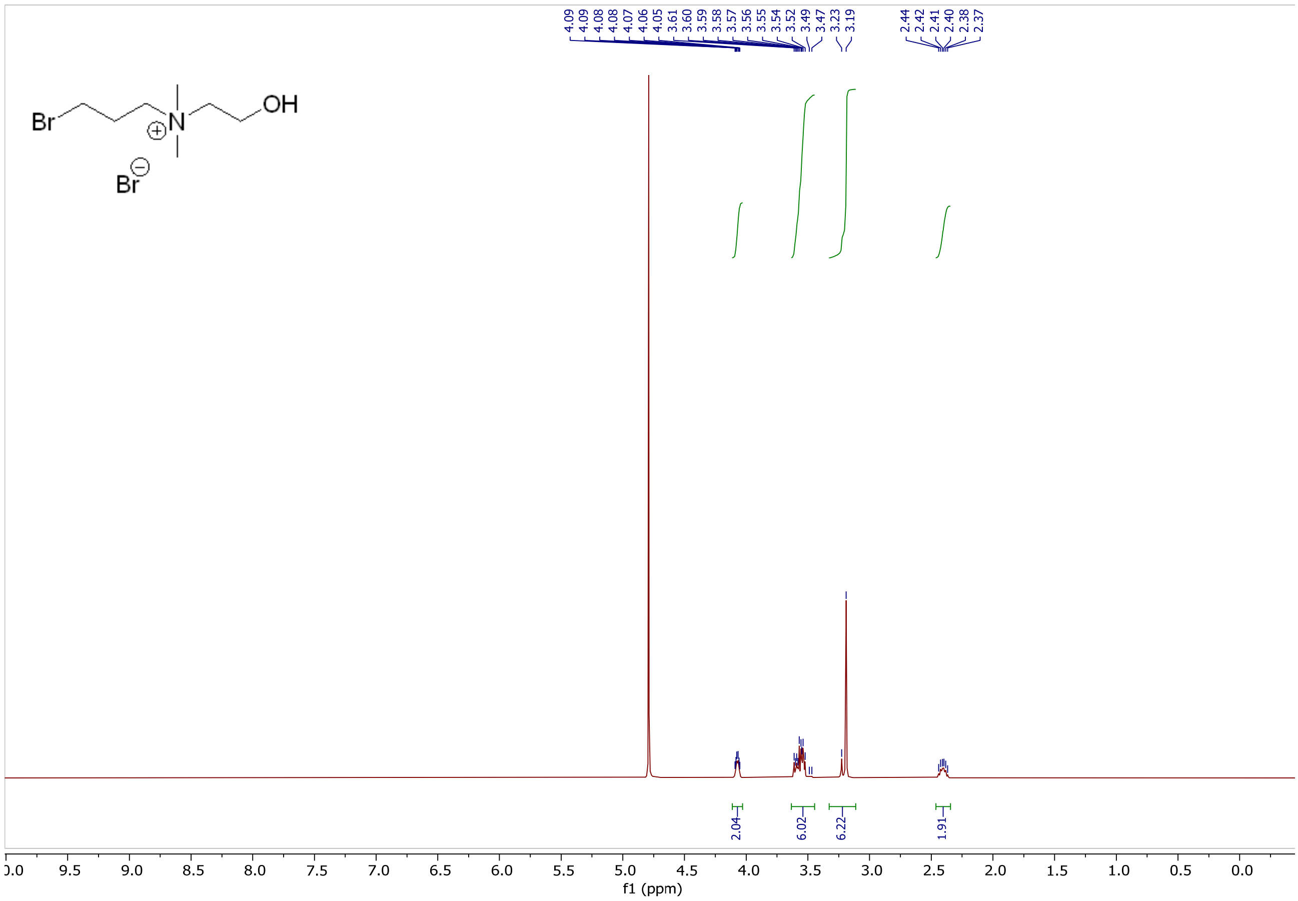

Fig. S15: (DMAE-BPr) ^{1}H-NMR (D_2O).

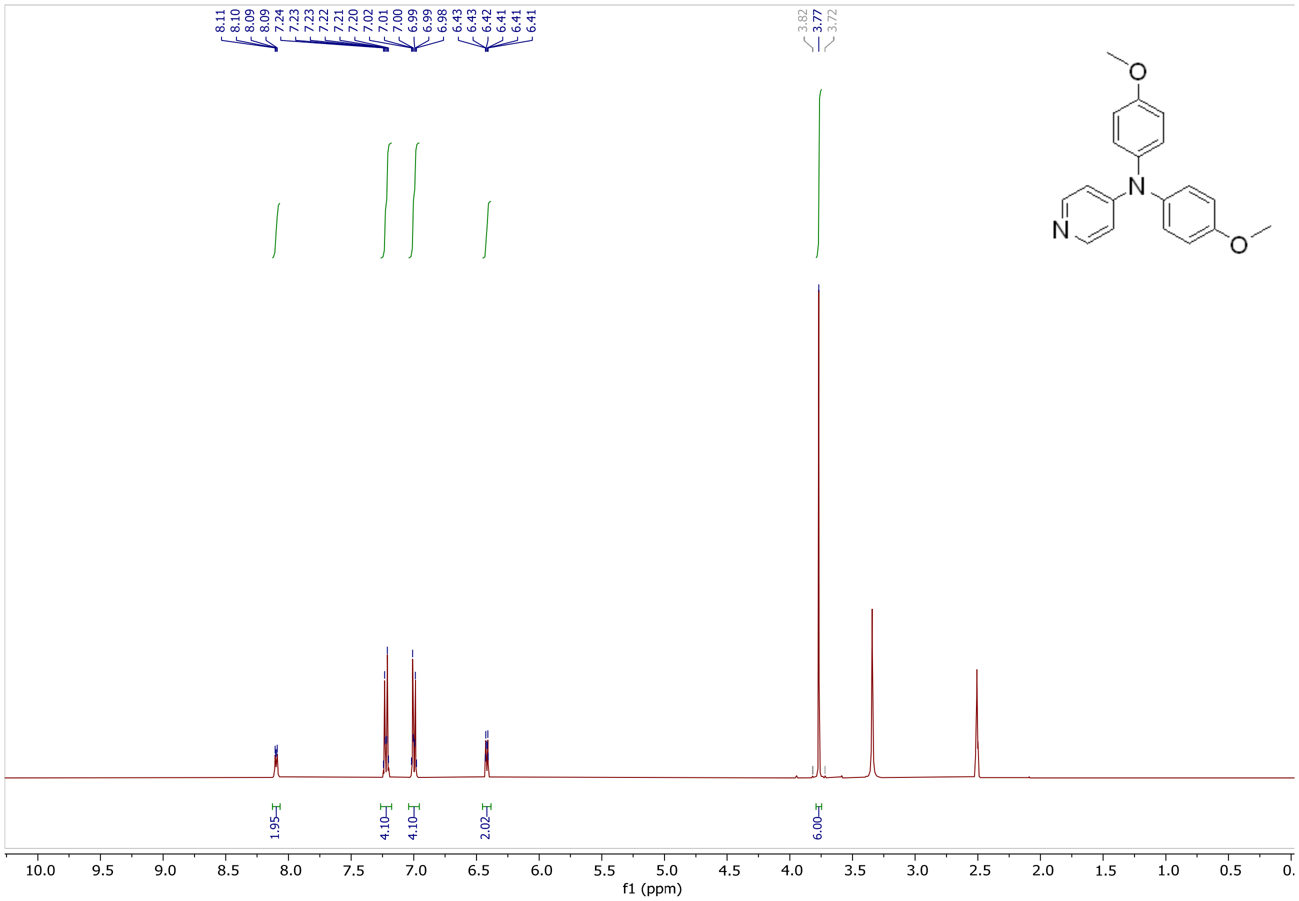

Fig. S16: (BMP)PA ^{1}H-NMR (dmso-d_6).

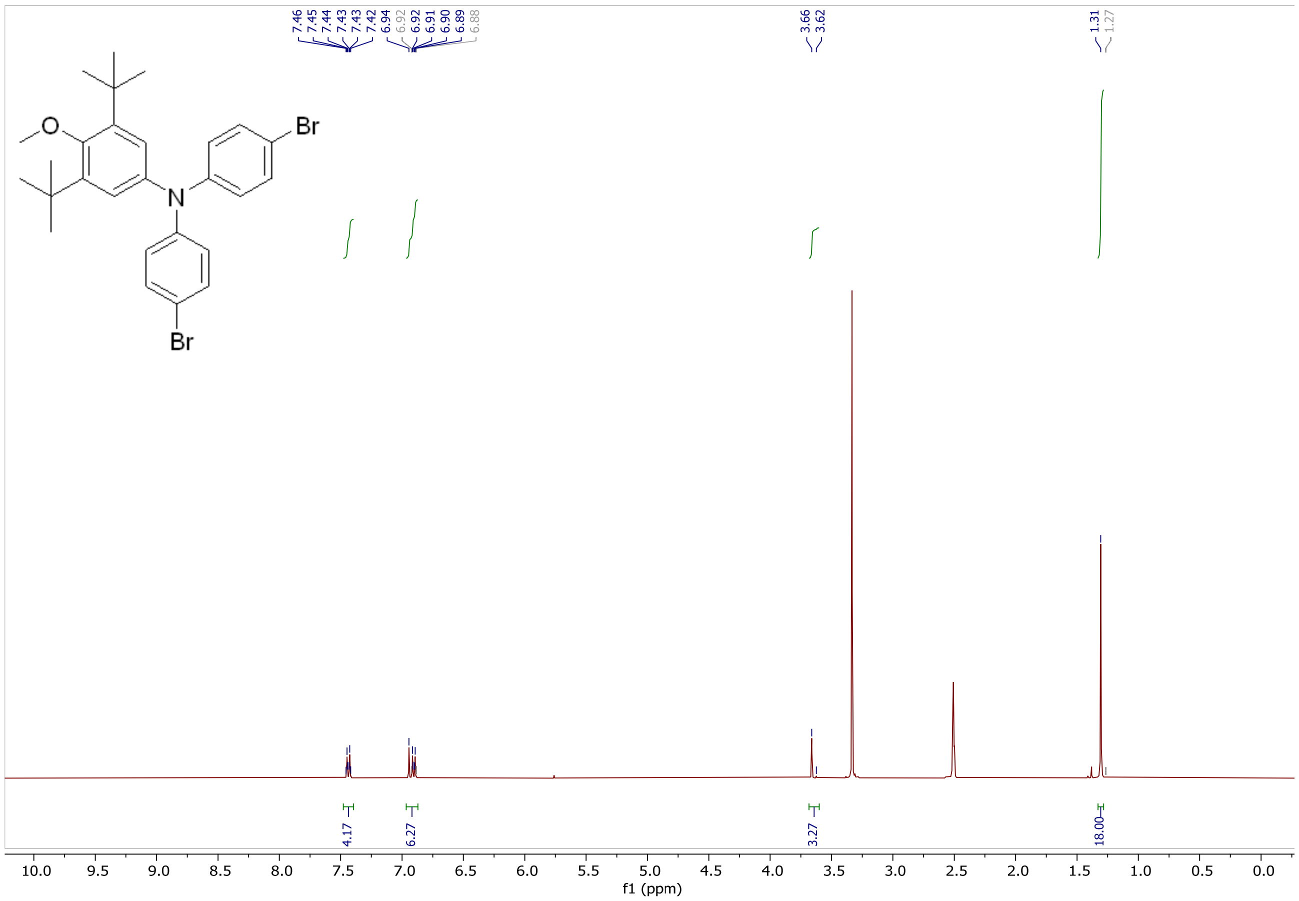

Fig. S17: Compound **11** ^{1}H-NMR (dmso-d_6).

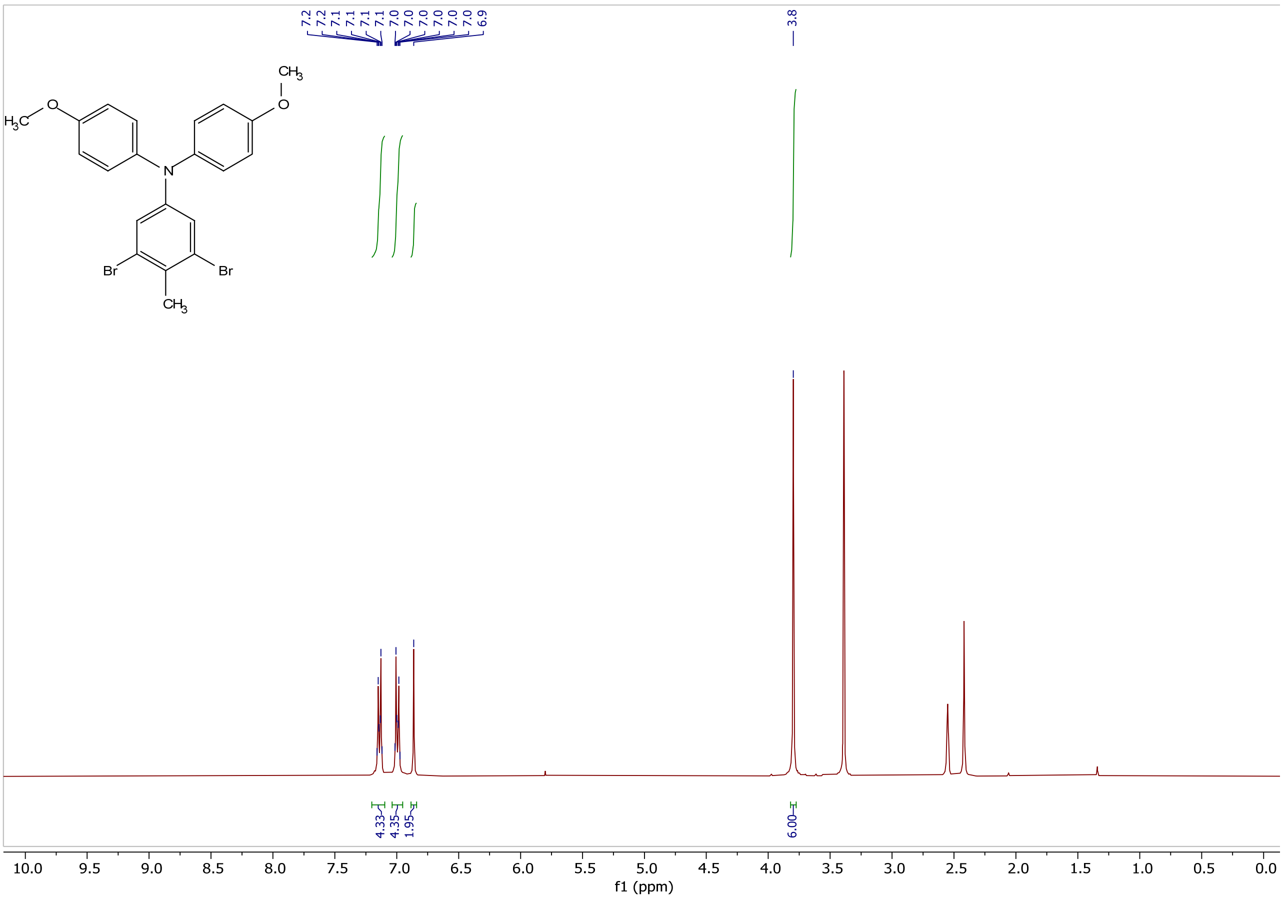

Fig. S18: Compound **12** ^{1}H-NMR (dmso-d_6).

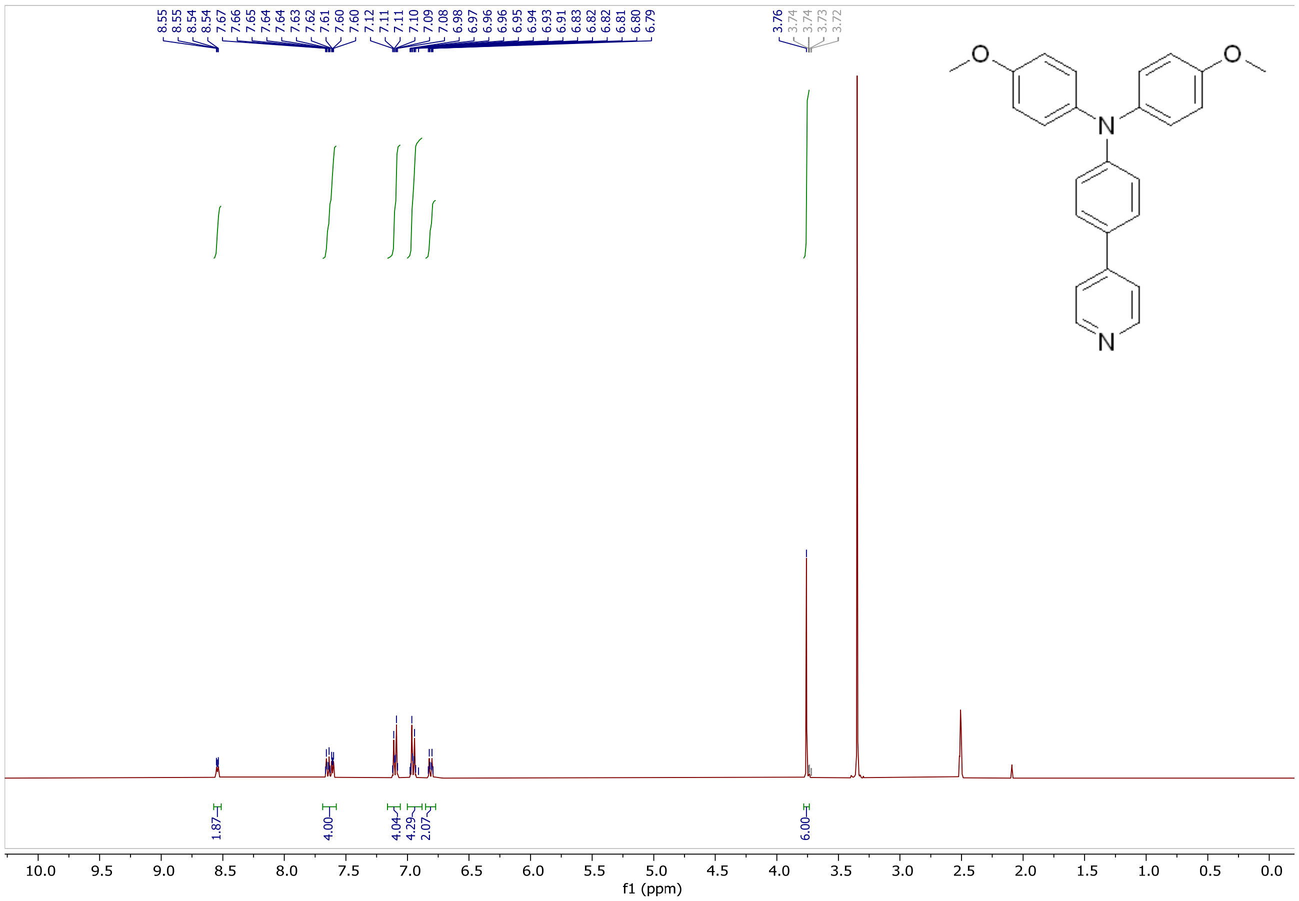

Fig. S18: Compound **13** ^{1}H-NMR (dmso-d_6).

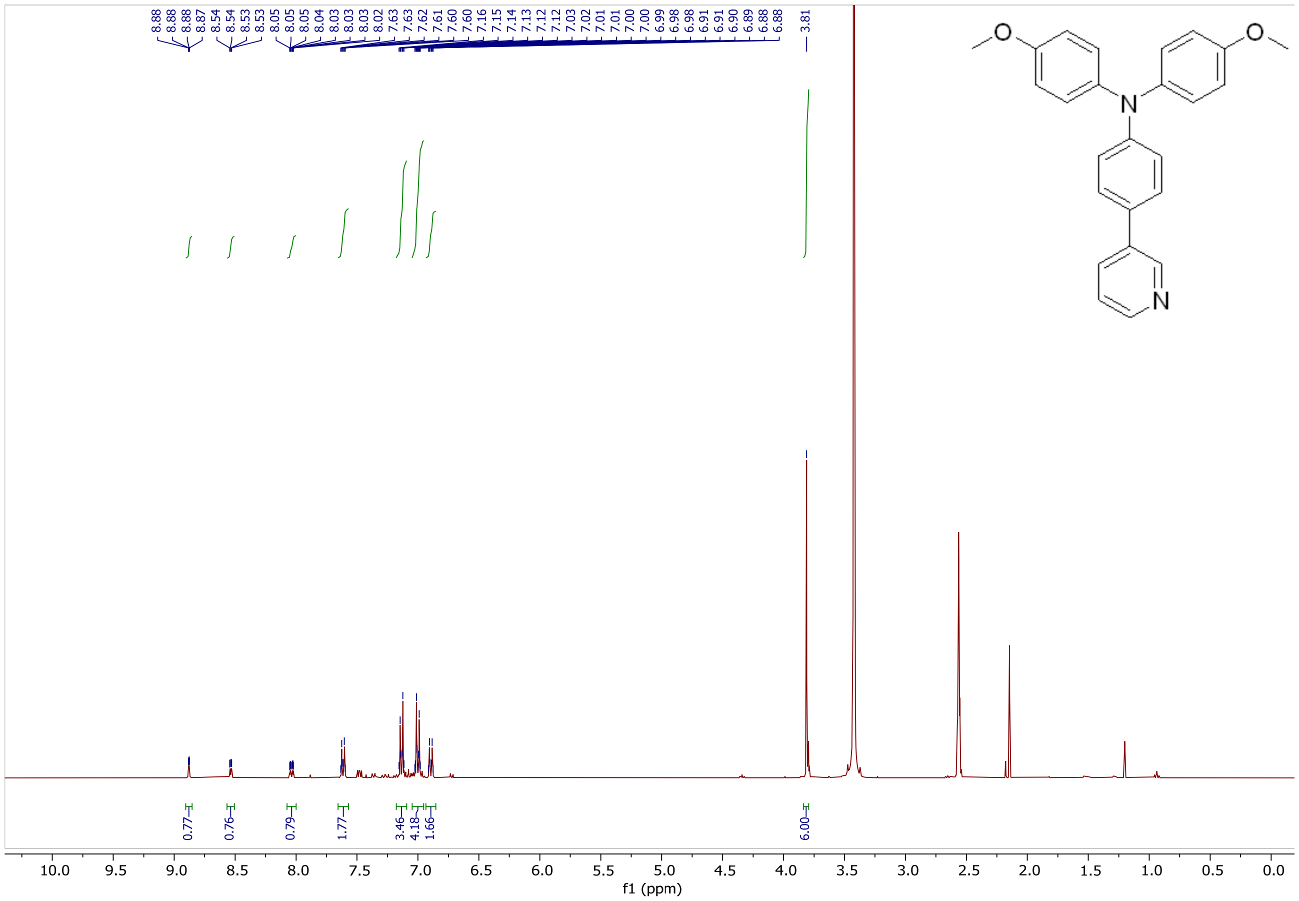

Fig. S19: Compound **14** ^{1}H-NMR (dmso-d_6).

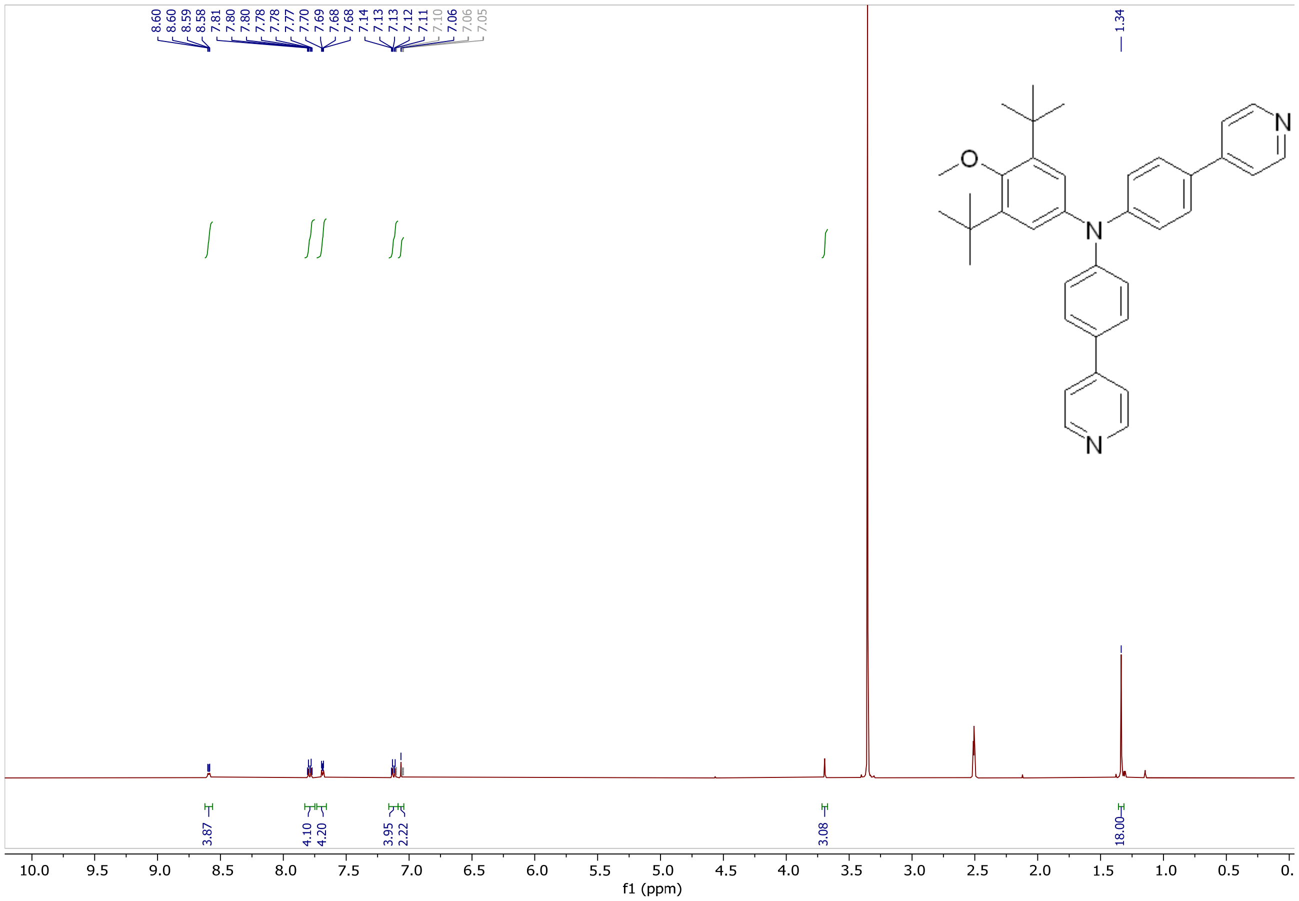

Fig. S20: Compound **15** ^{1}H-NMR (dmso-d_6).

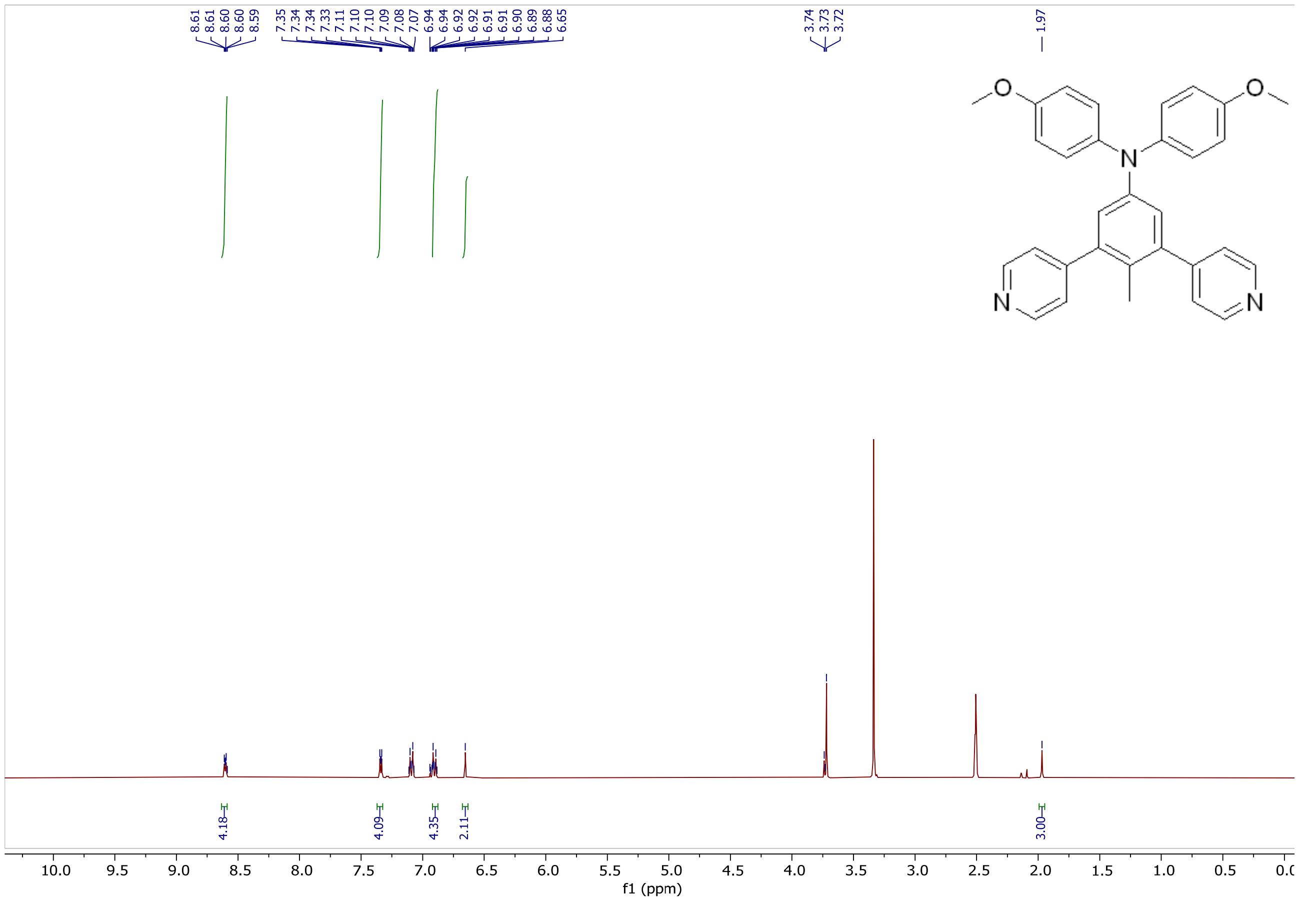

Fig. S21: Compound **16** ^{1}H-NMR (dmso-d$_6$).

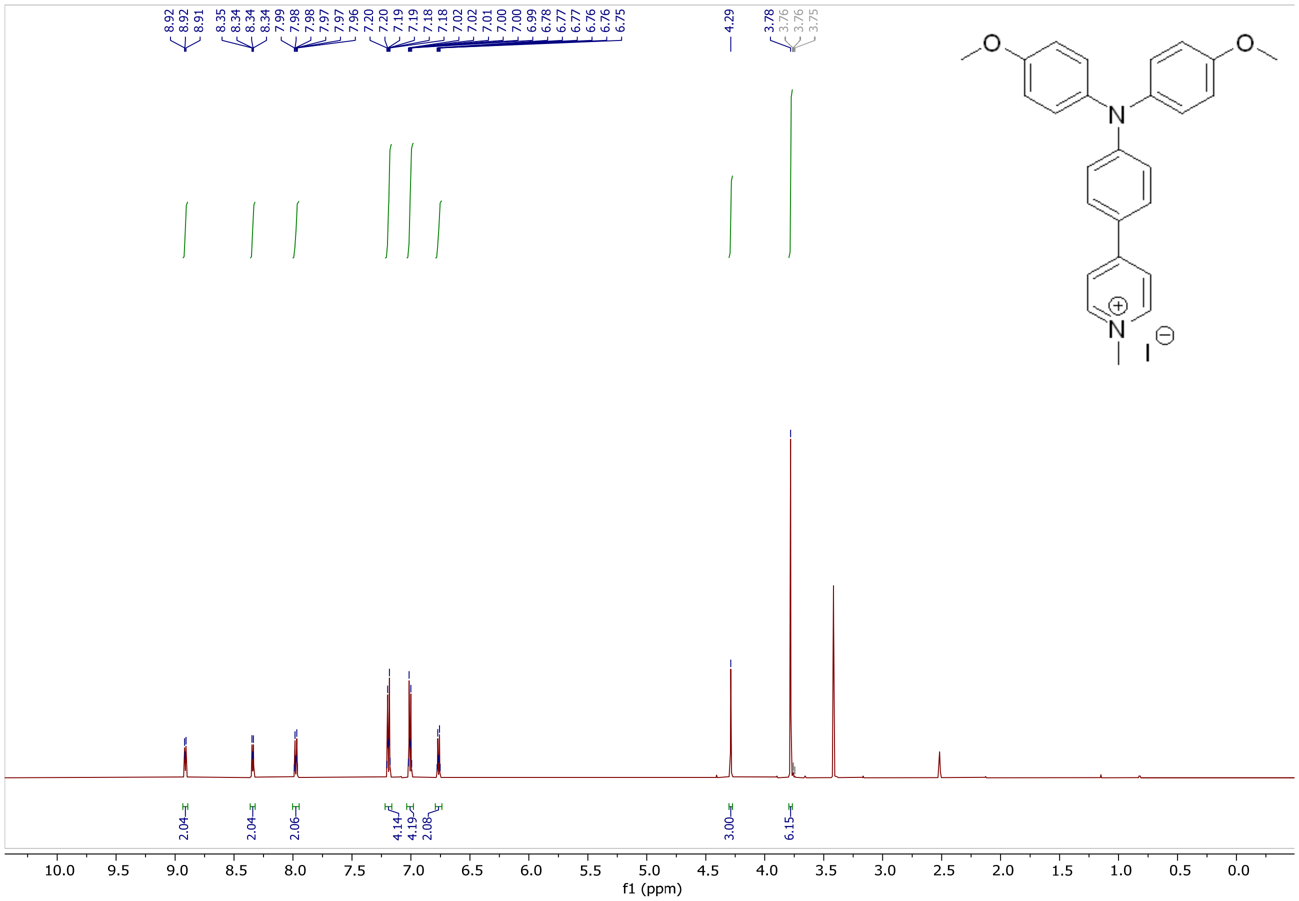

Fig. S22: Compound **1** ^{1}H-NMR (600 MHz, dmso-d_6).

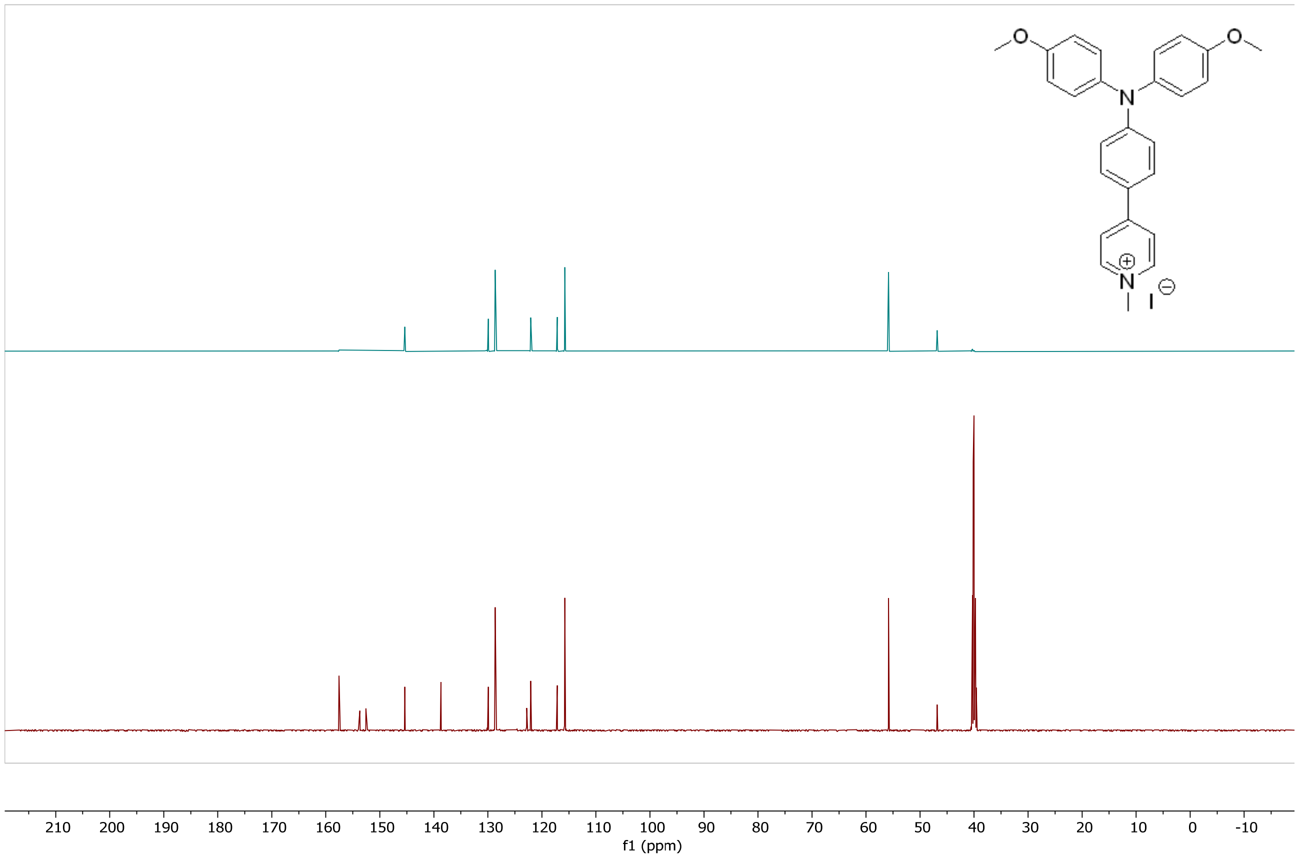

Fig. S23: Compound **1** ^{13}C-NMR (600 MHz, dmso-d$_6$): top (DEPT-90), bottom ^{13}C.

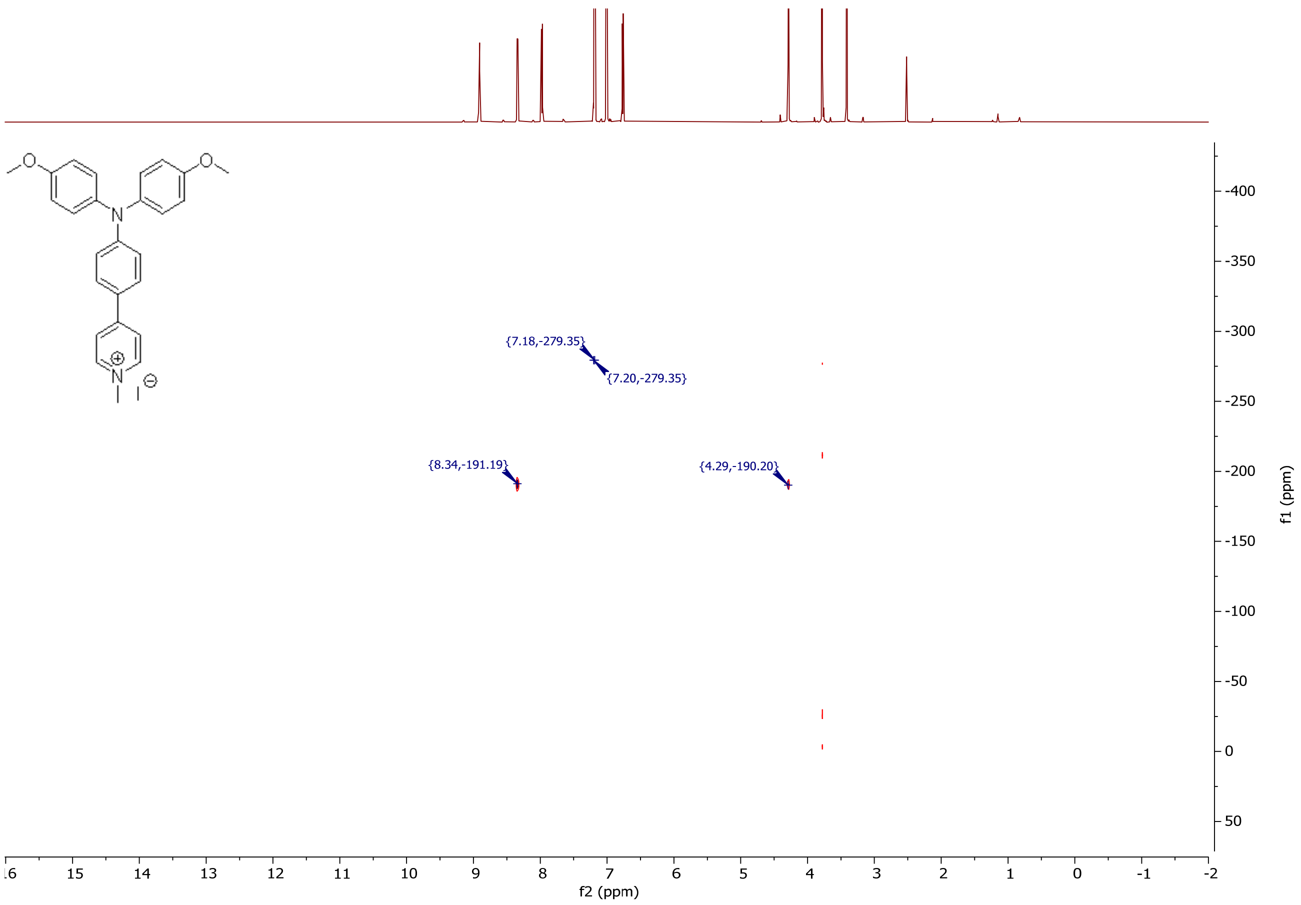

Fig. S24: Compound **1** ^{15}N-HMBC (600 MHz, dmso-d_6).

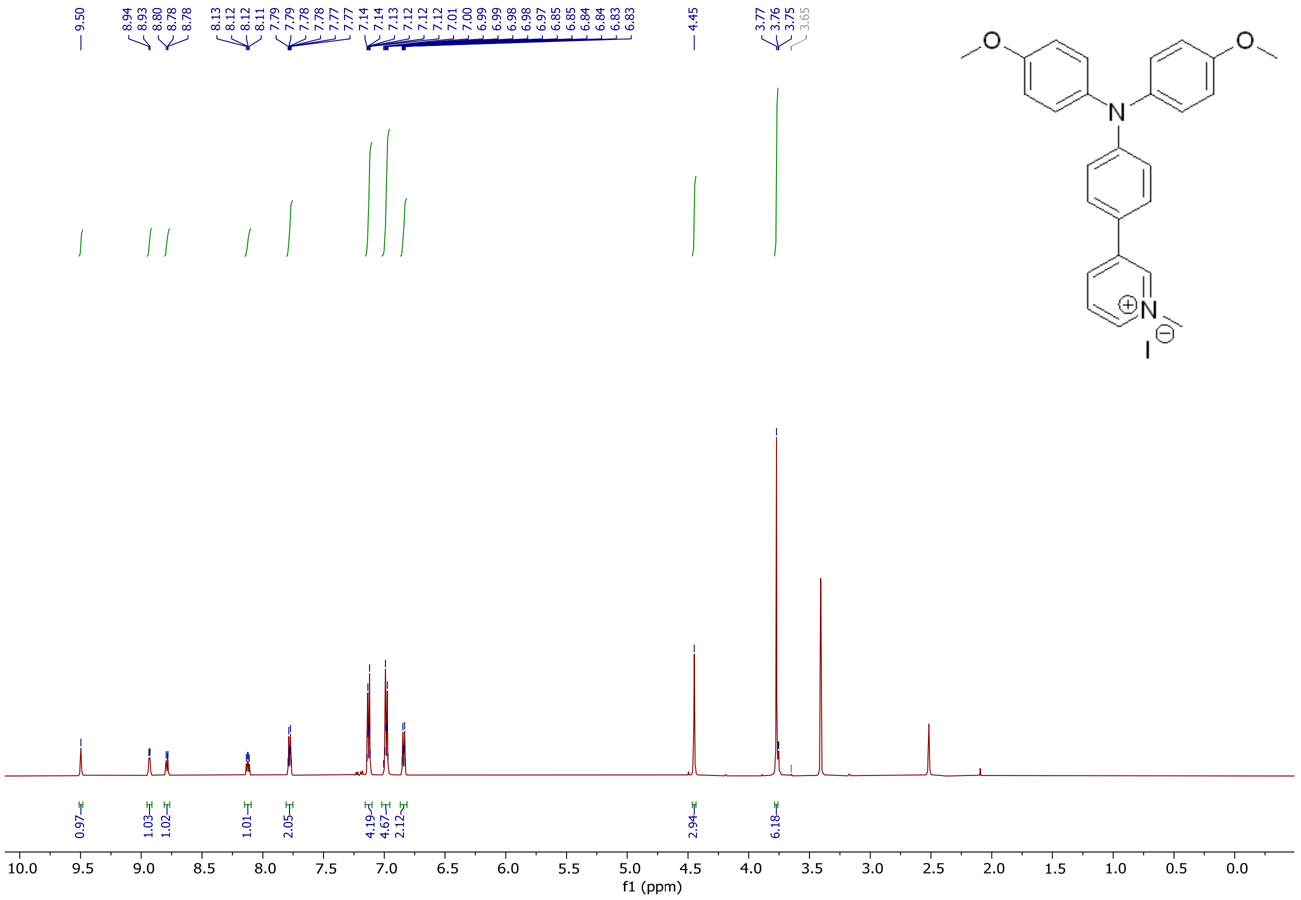

Fig. S25: Compound **2** ^{1}H-NMR (600 MHz, dmso-d_6).

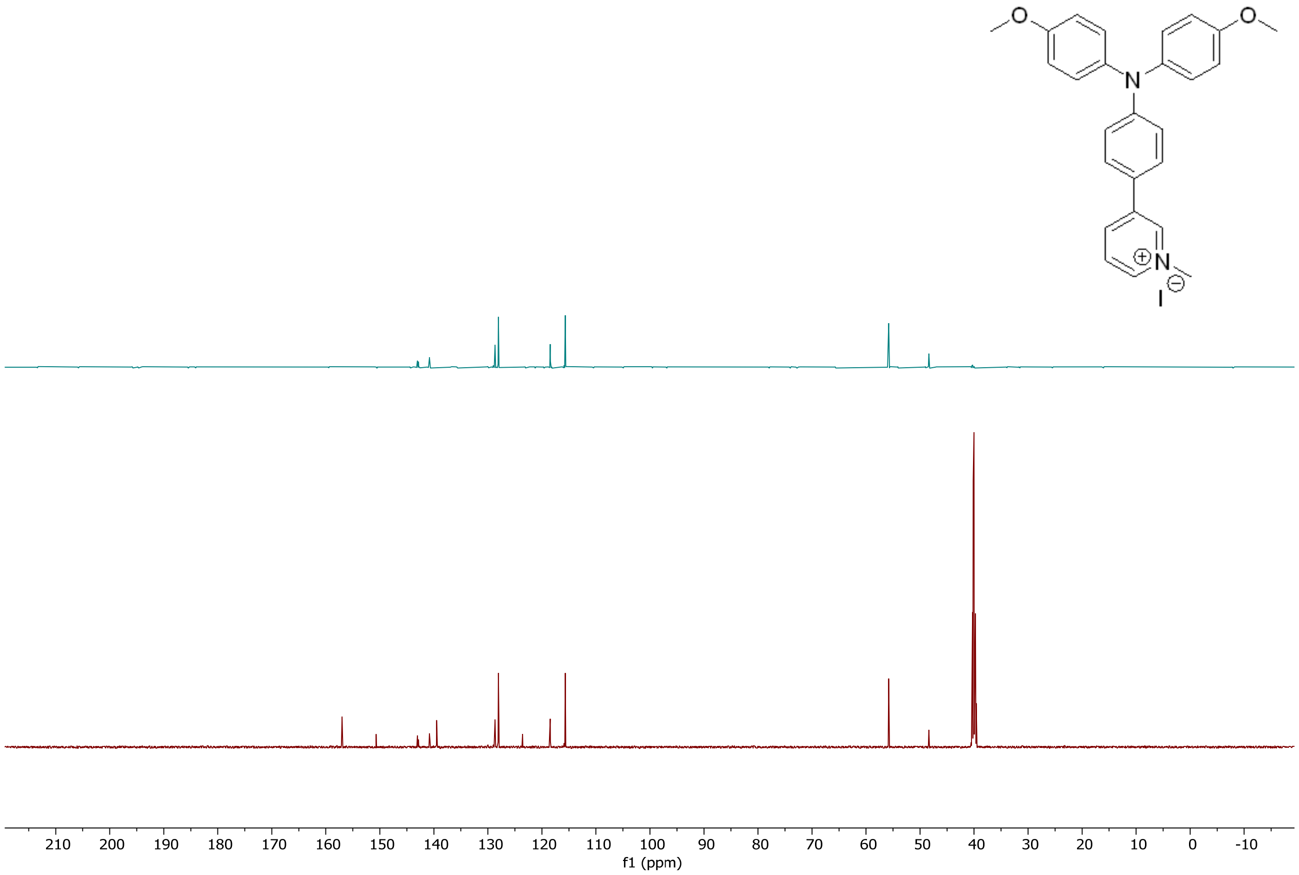

Fig. S26: Compound **2** ^{13}C-NMR (600 MHz, dmso-d$_6$): top (DEPT-90), bottom ^{13}C.

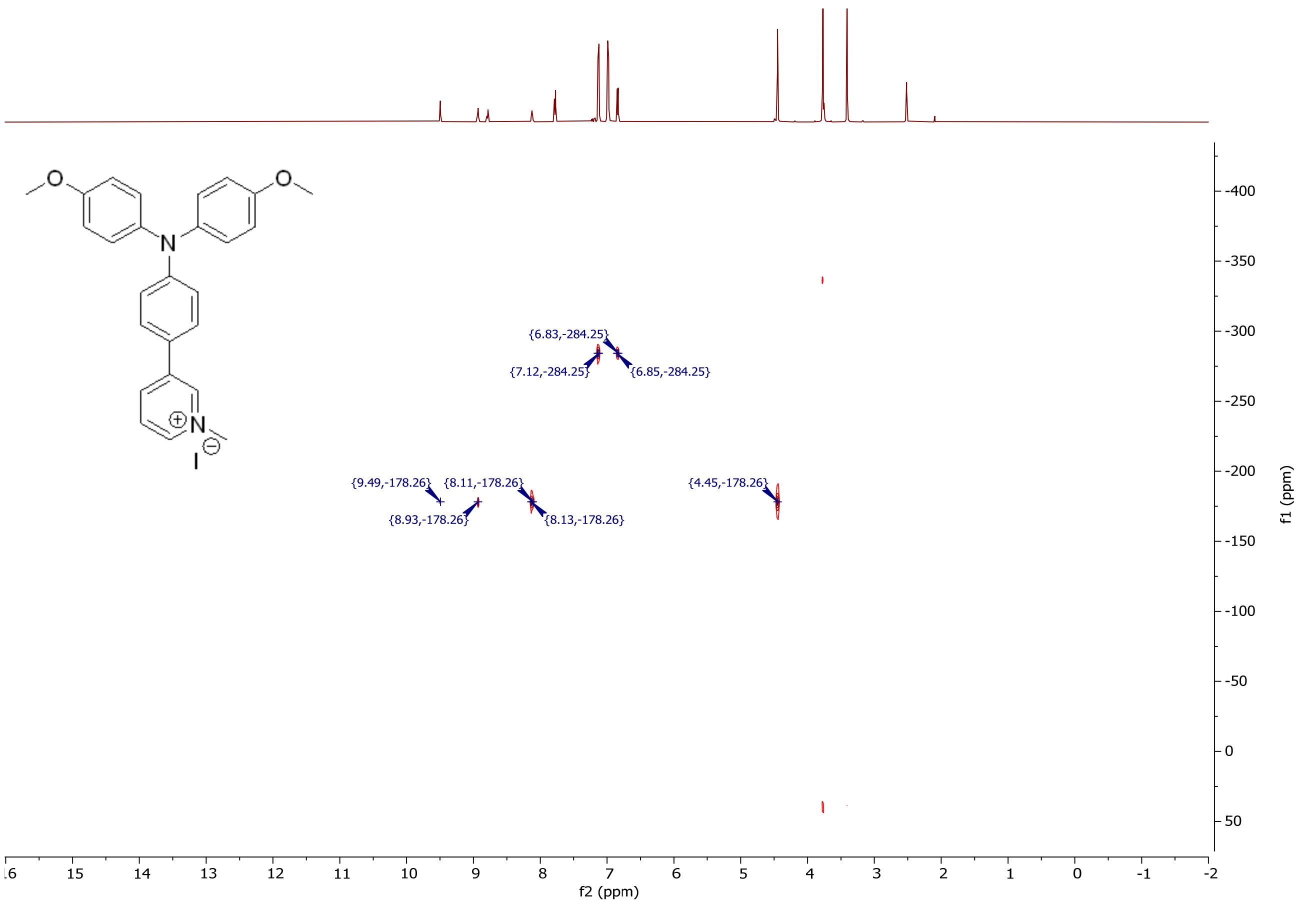

Fig. S27: Compound **2** ^{15}N-HMBC (600 MHz, dmso-d$_6$).

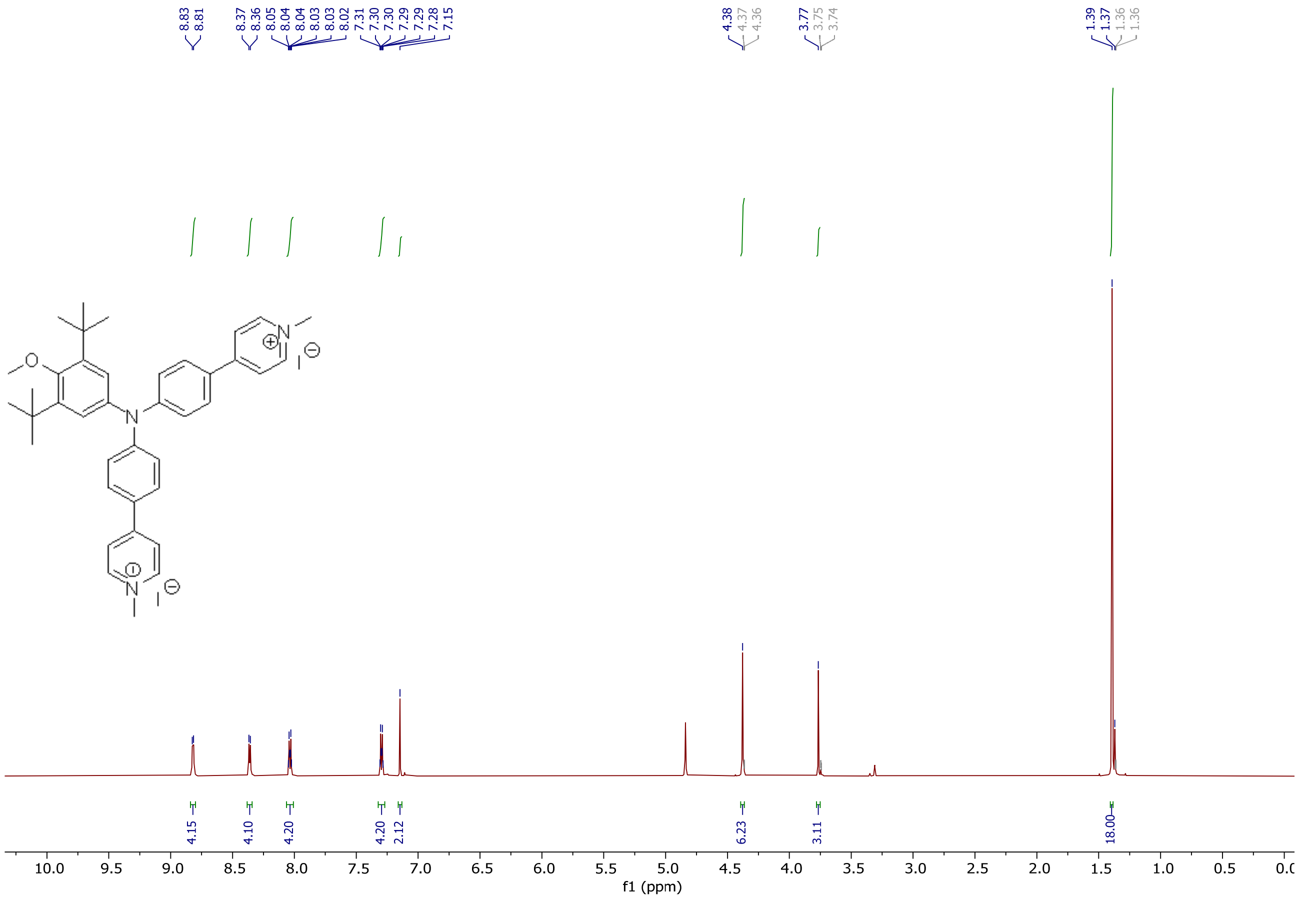

Fig. S28: Compound **3** ^{1}H-NMR (600 MHz, D_2O+TMSP-d_4).

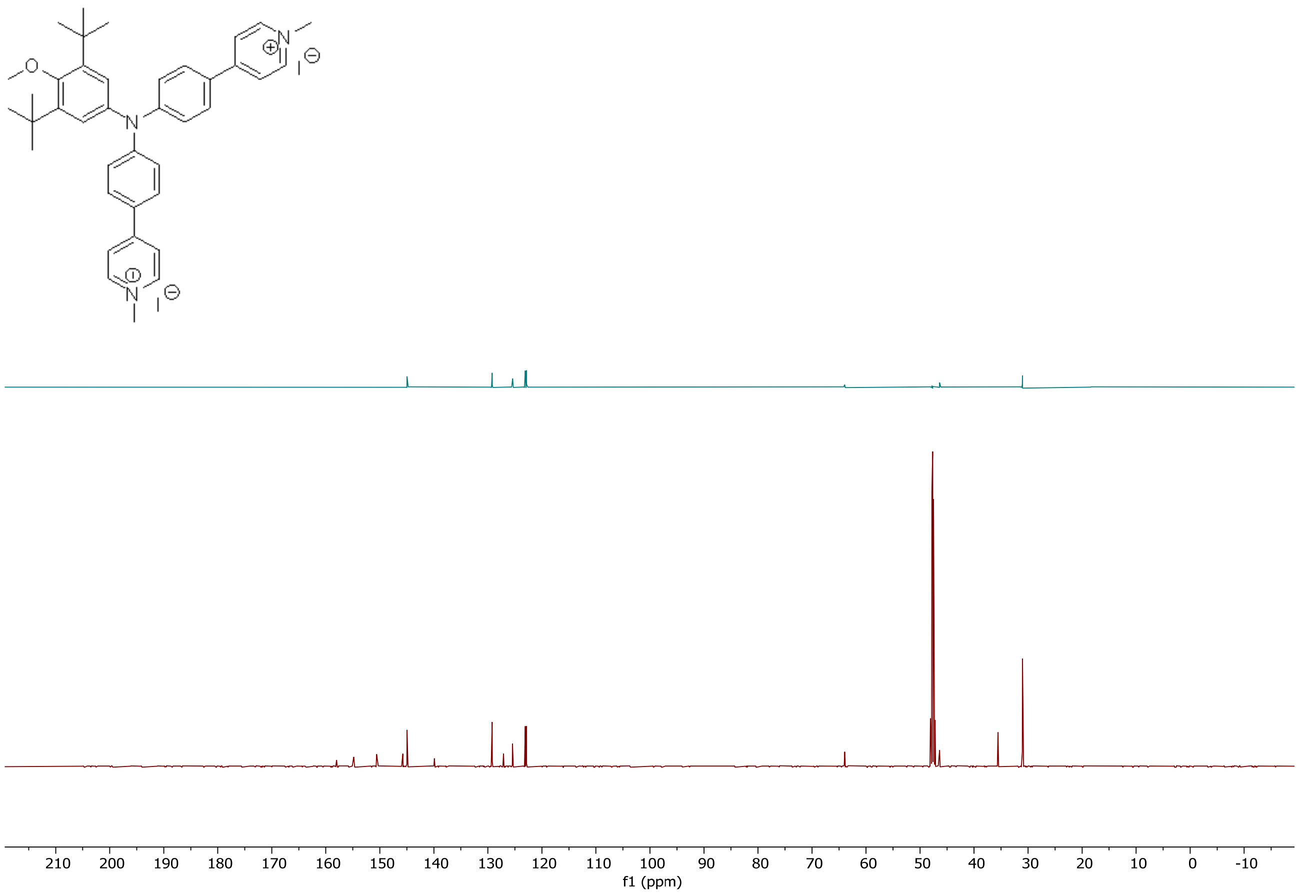

Fig. S29: Compound **3** ^{13}C-NMR (600 MHz, D_2O+TMSP-d$_4$): top (DEPT-90), bottom ^{13}C.

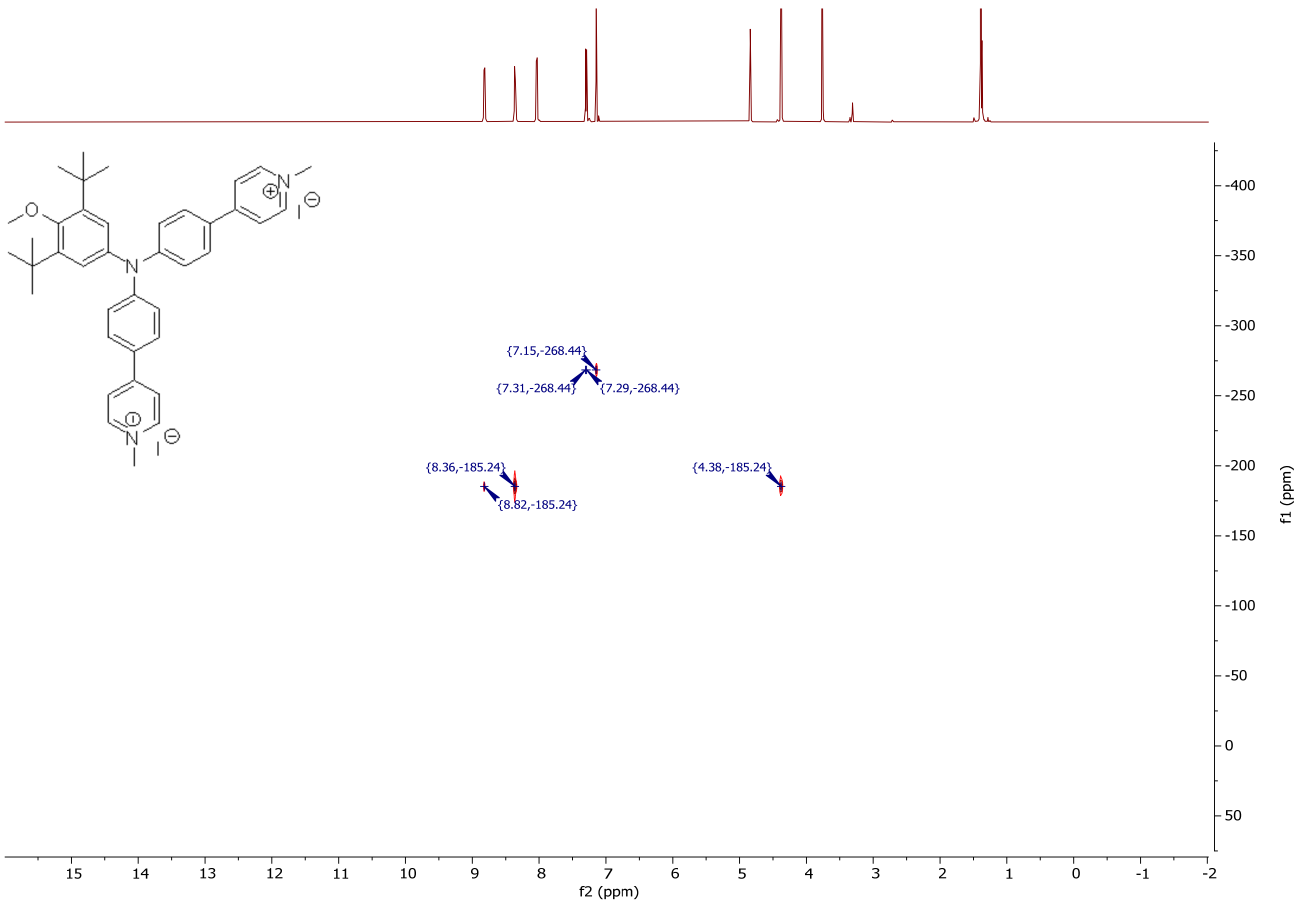

Fig. S30: Compound **3** ^{15}N-HMBC (600 MHz, D_2O).

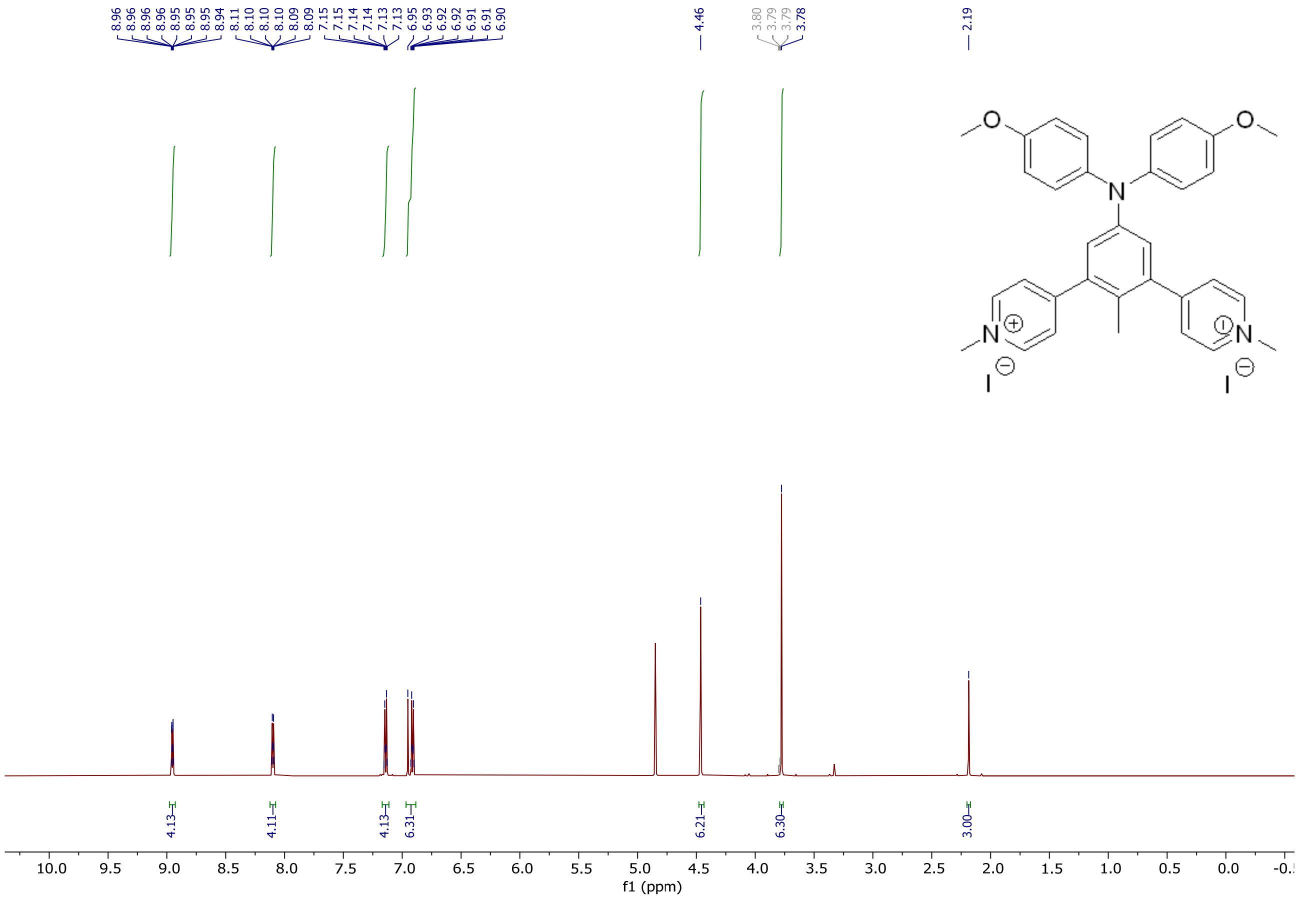

Fig. S31: Compound **4** ^{1}H-NMR (600 MHz, CD_3OD).

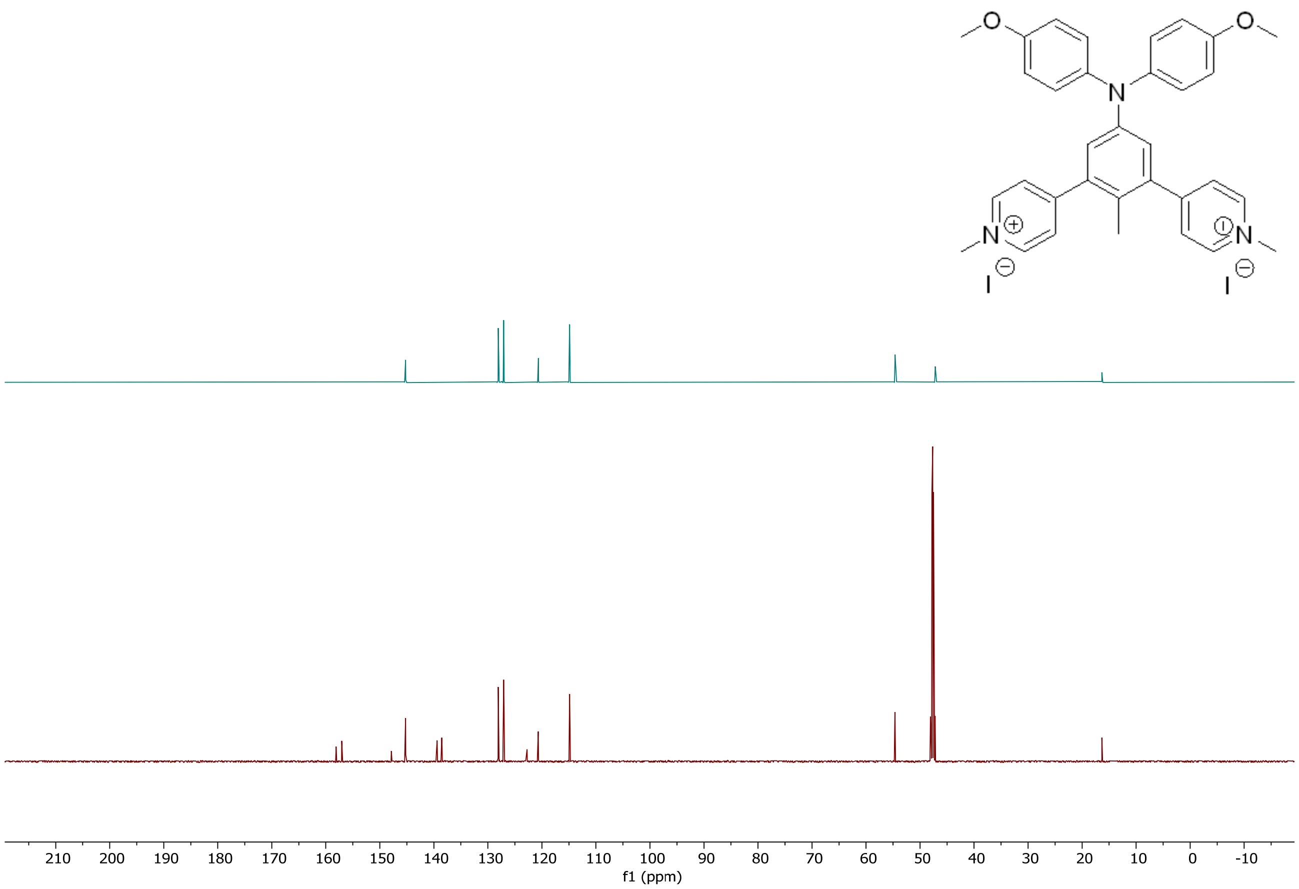

Fig. S32: Compound **4** ^{13}C-NMR (600 MHz, CD_3OD): top (DEPT-90), bottom ^{13}C.

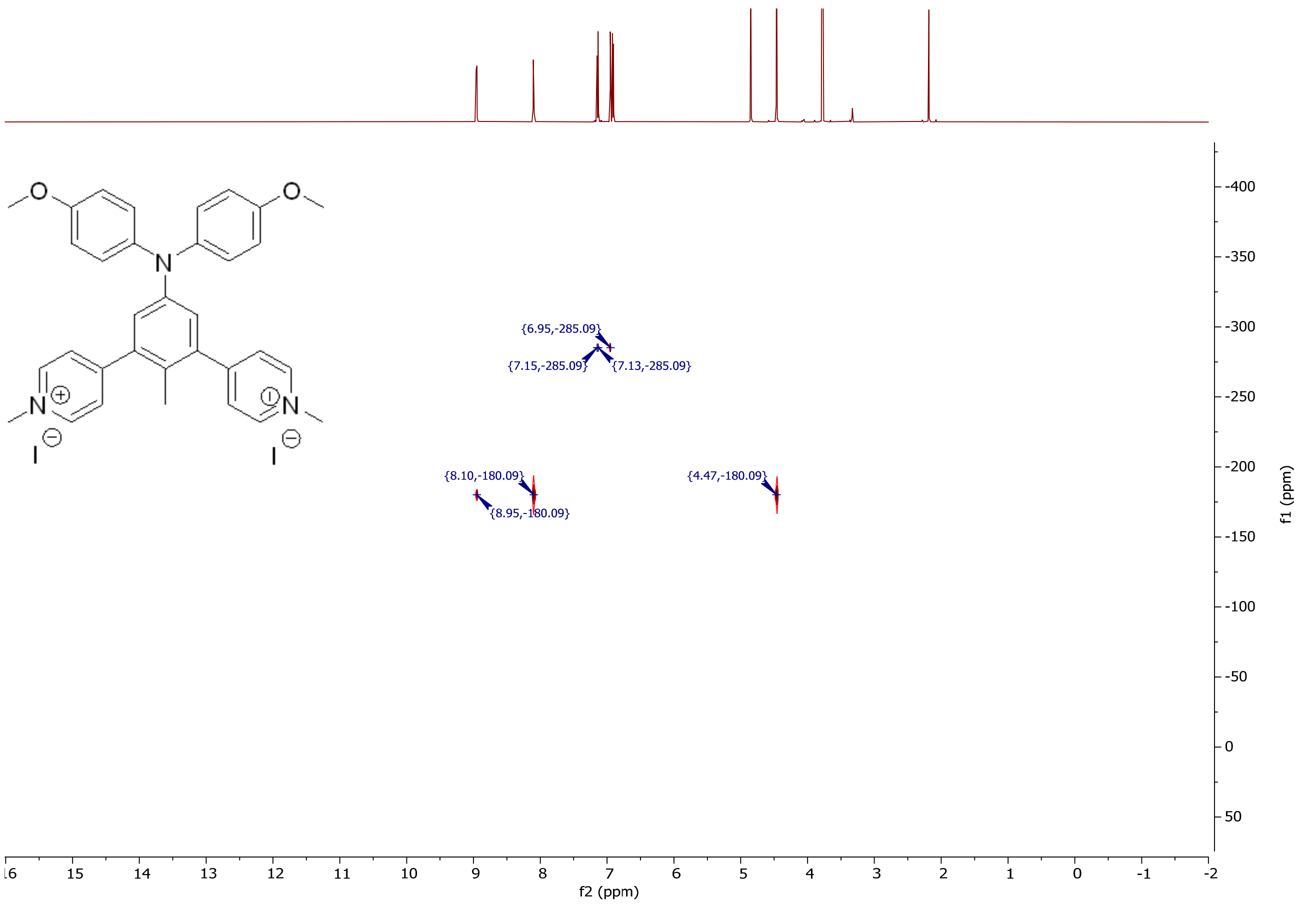

Fig. S33: Compound **4** ^{15}N-HMBC (600 MHz, CD_3OD).

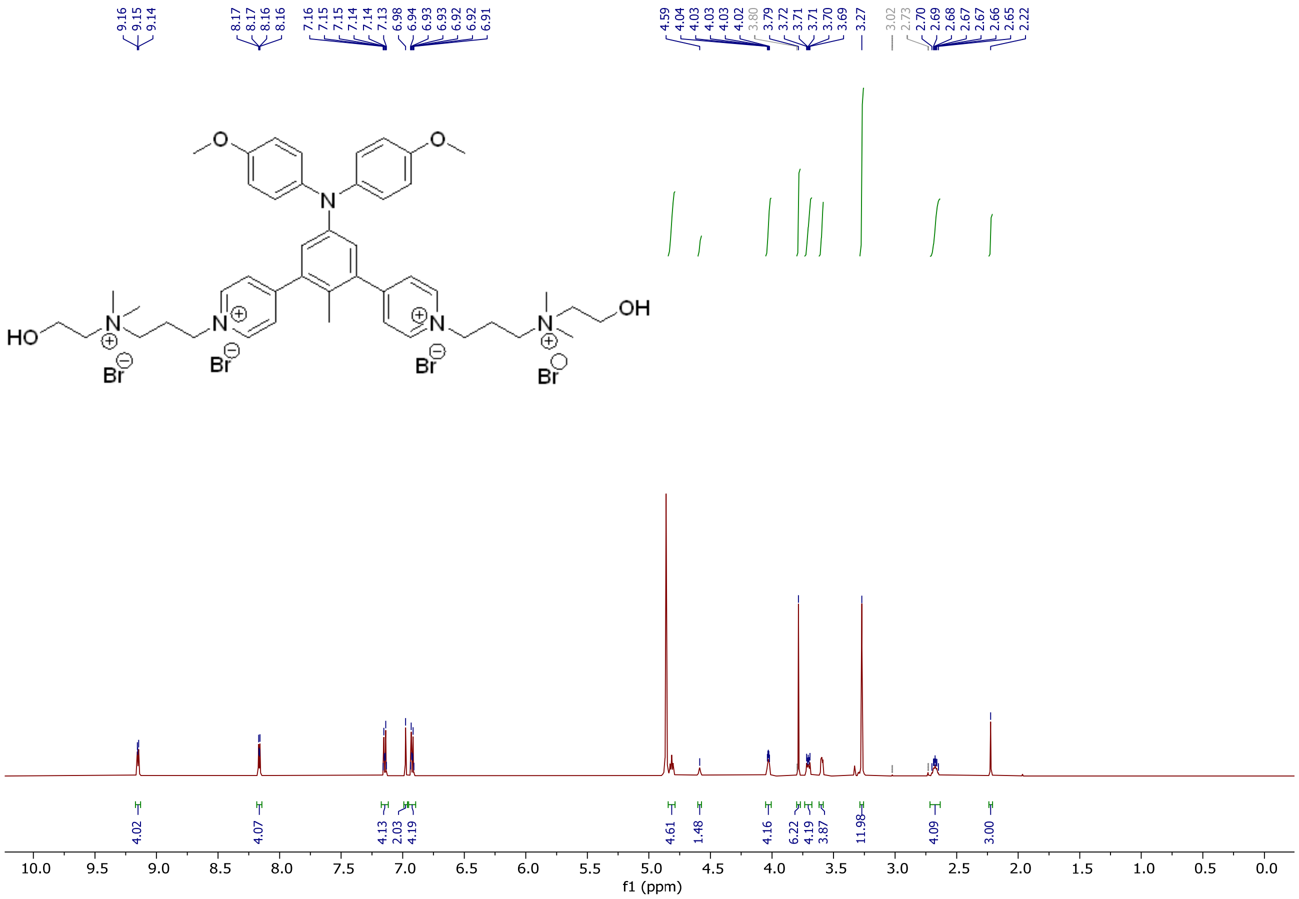

Fig. S34: Compound **5** ^{1}H-NMR (600 MHz, CD_3OD).

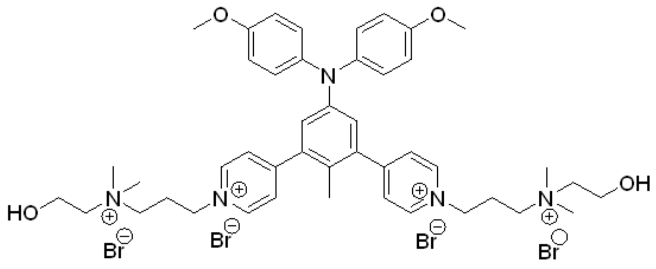

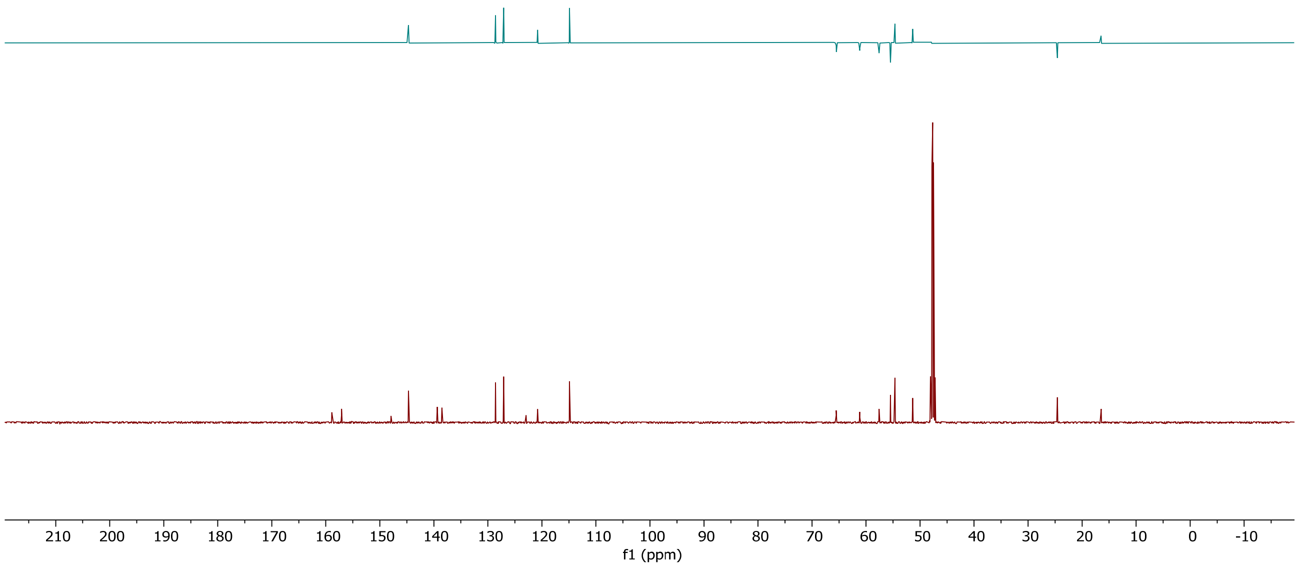

Fig. S35: Compound **5** ^{13}C-NMR (600 MHz, CD_3OD): top (DEPT-90), bottom ^{13}C.

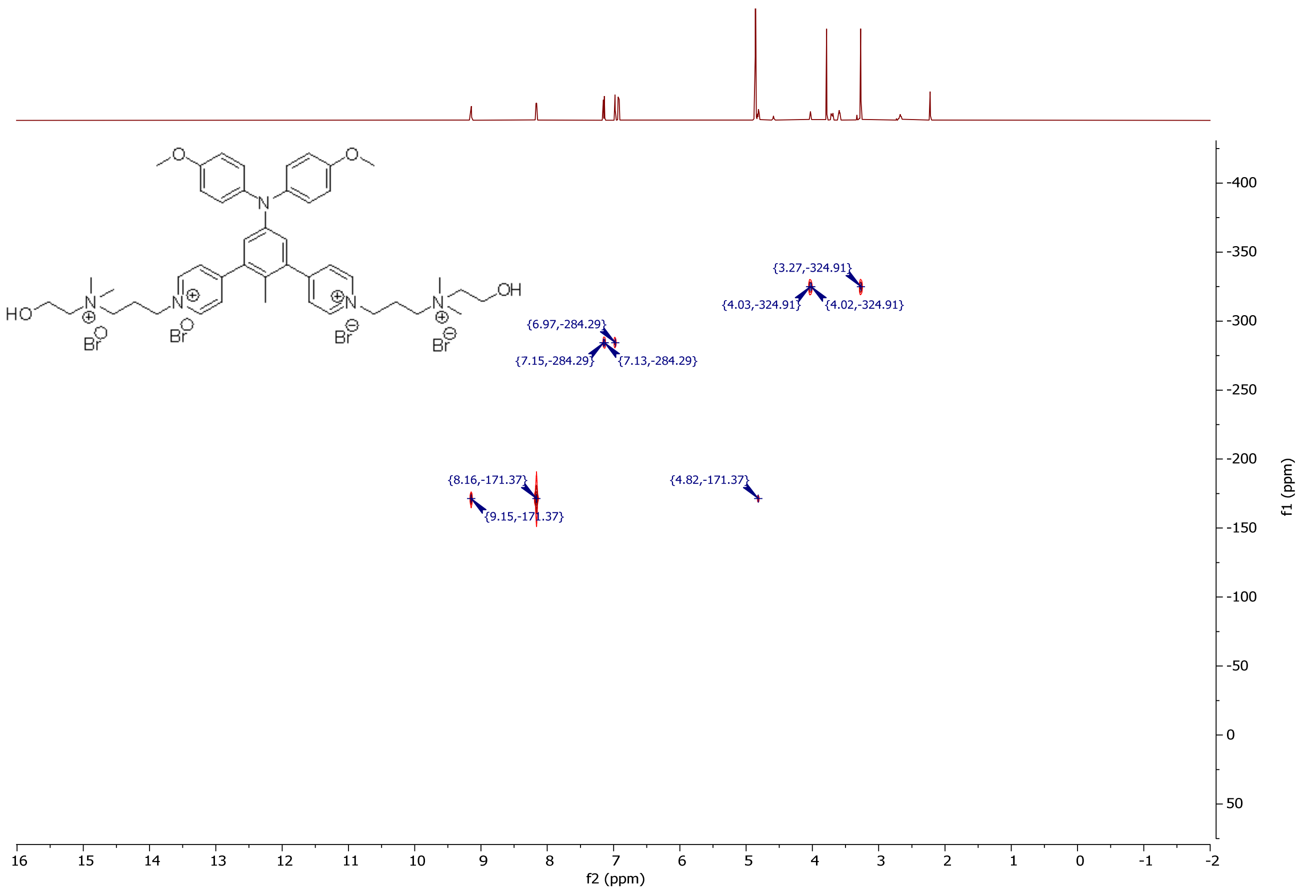

Fig. S36: Compound **5** ^{15}N-HMBC (600 MHz, CD_3OD).

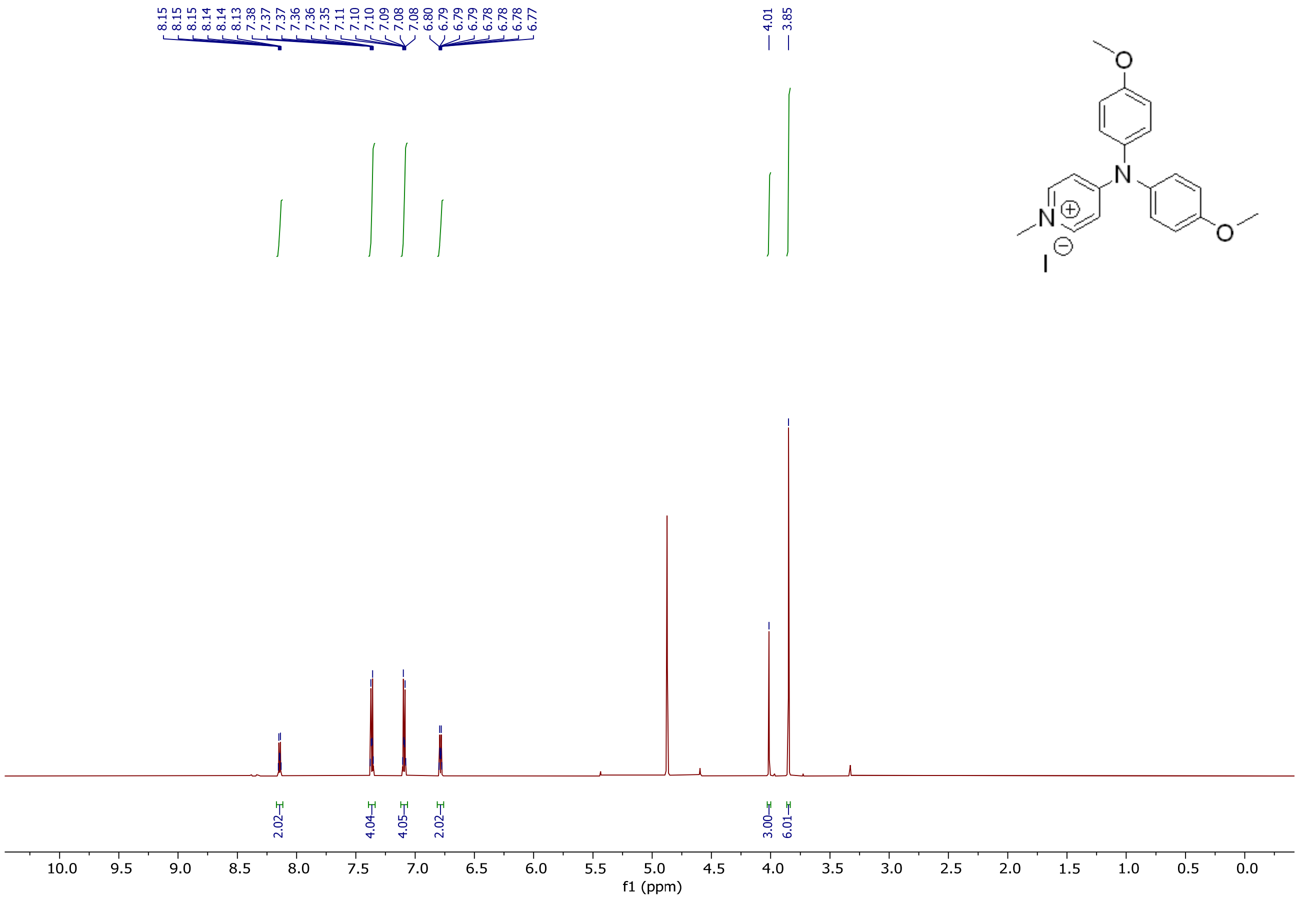

Fig. S37: Compound **6** ^{1}H-NMR (600 MHz, CD_3OD).

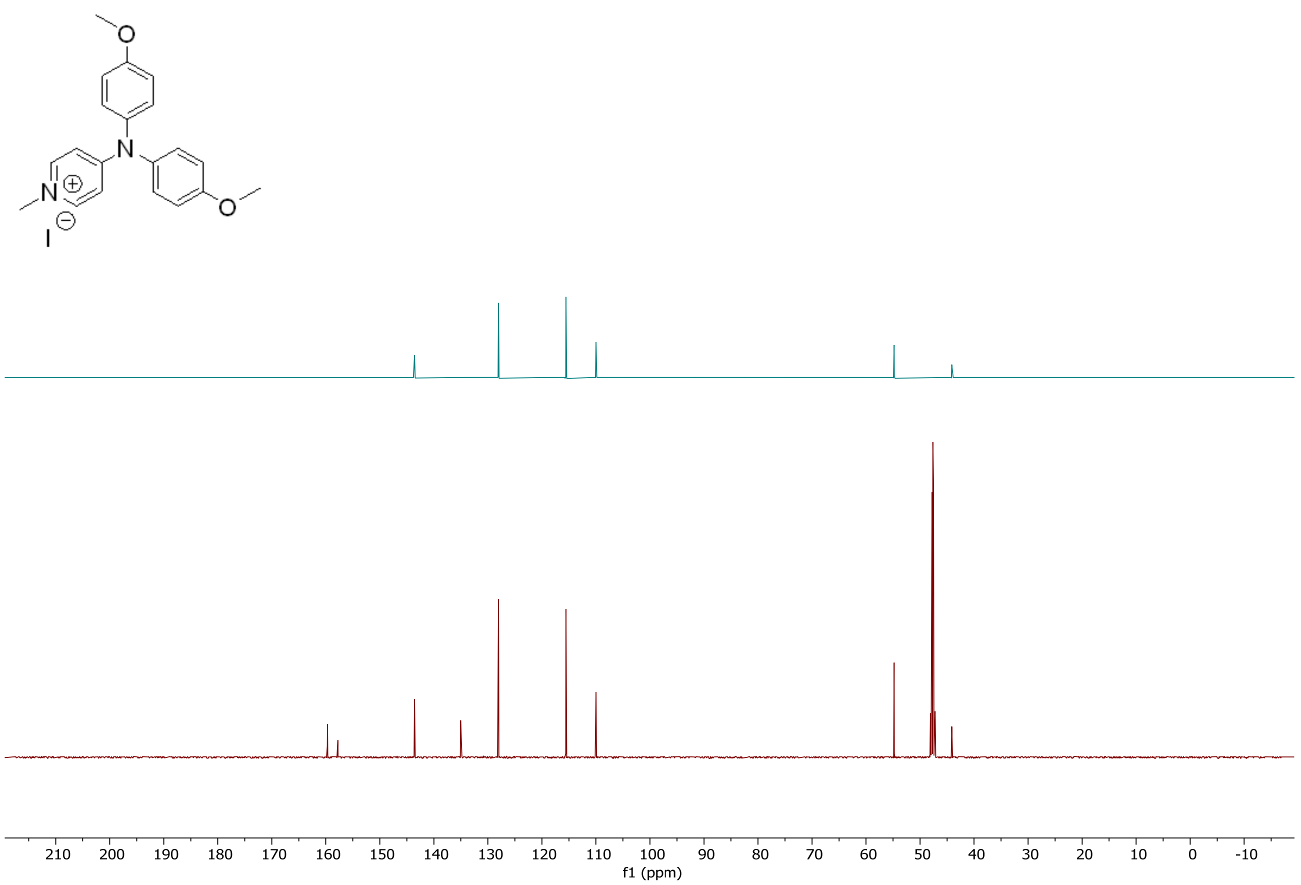

Fig. S38: Compound **6** ^{13}C-NMR (600 MHz, CD_3OD): top (DEPT-90), bottom ^{13}C.

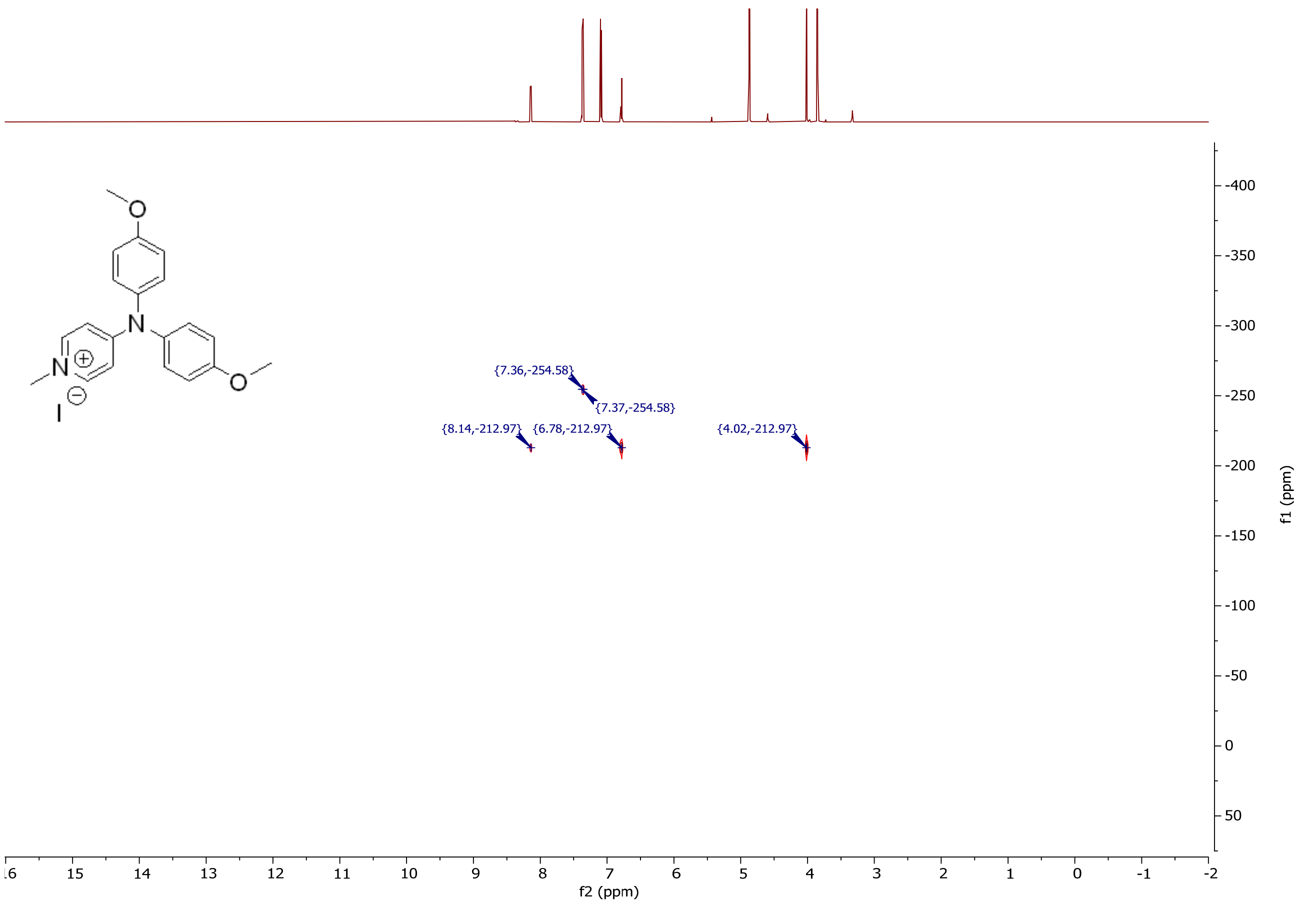

Fig. S39: Compound **6** ^{15}N-HMBC (600 MHz, CD_3OD).

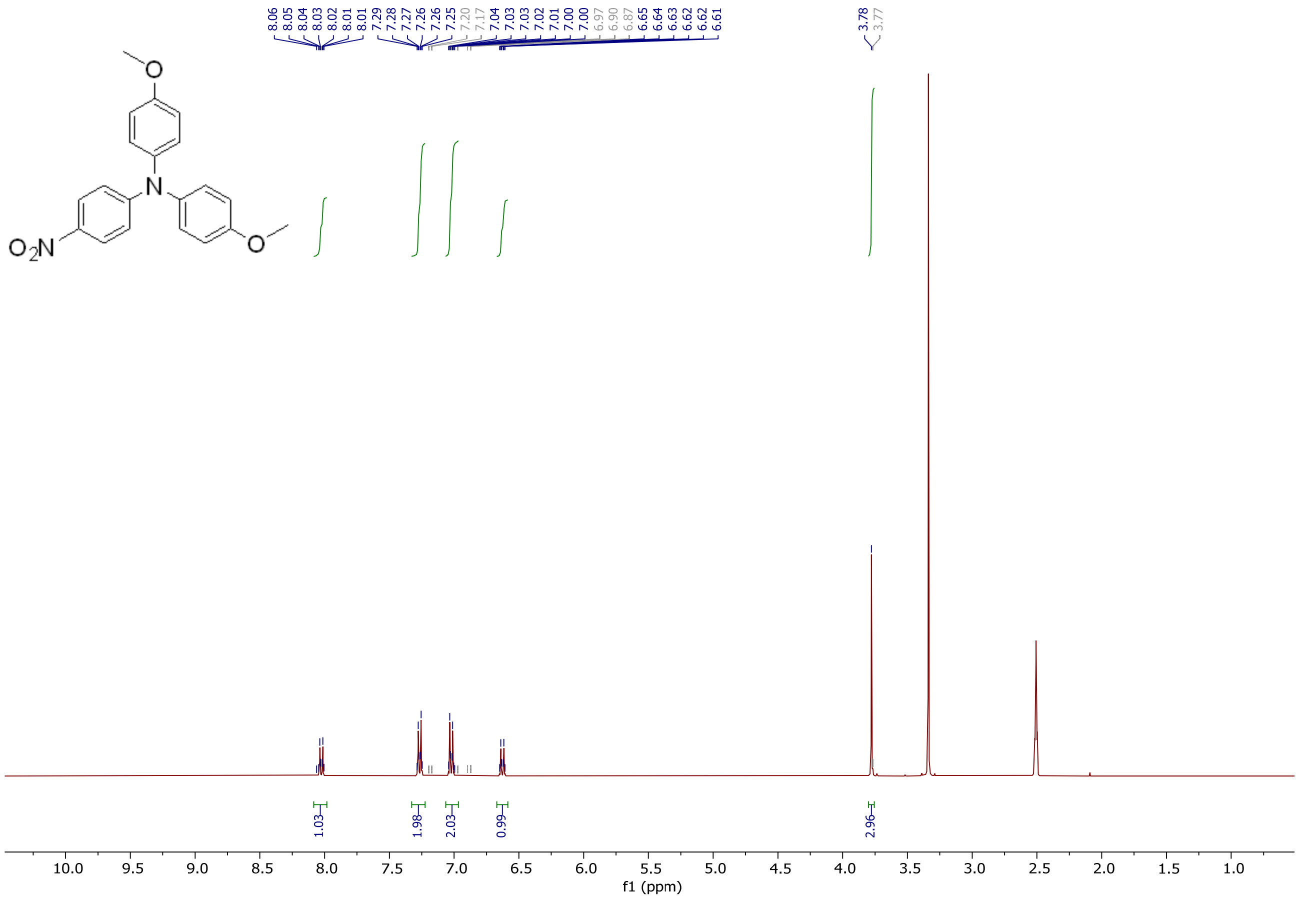

Fig. S40: Compound **17** ^{1}H-NMR (400 MHz, dmso-d_6).

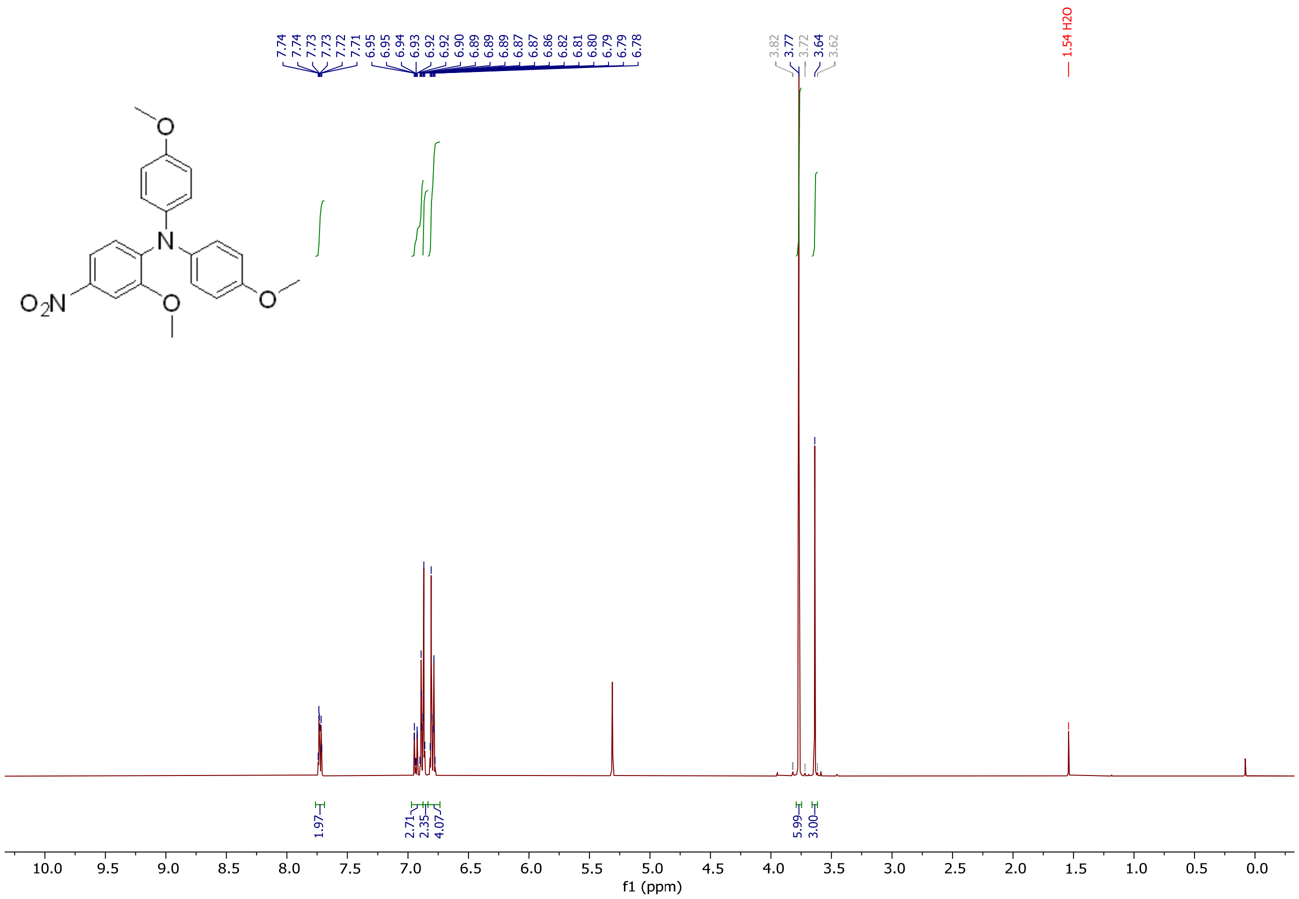

Fig. S41: Compound **18** ^{1}H-NMR (400 MHz, DCM-d_2).

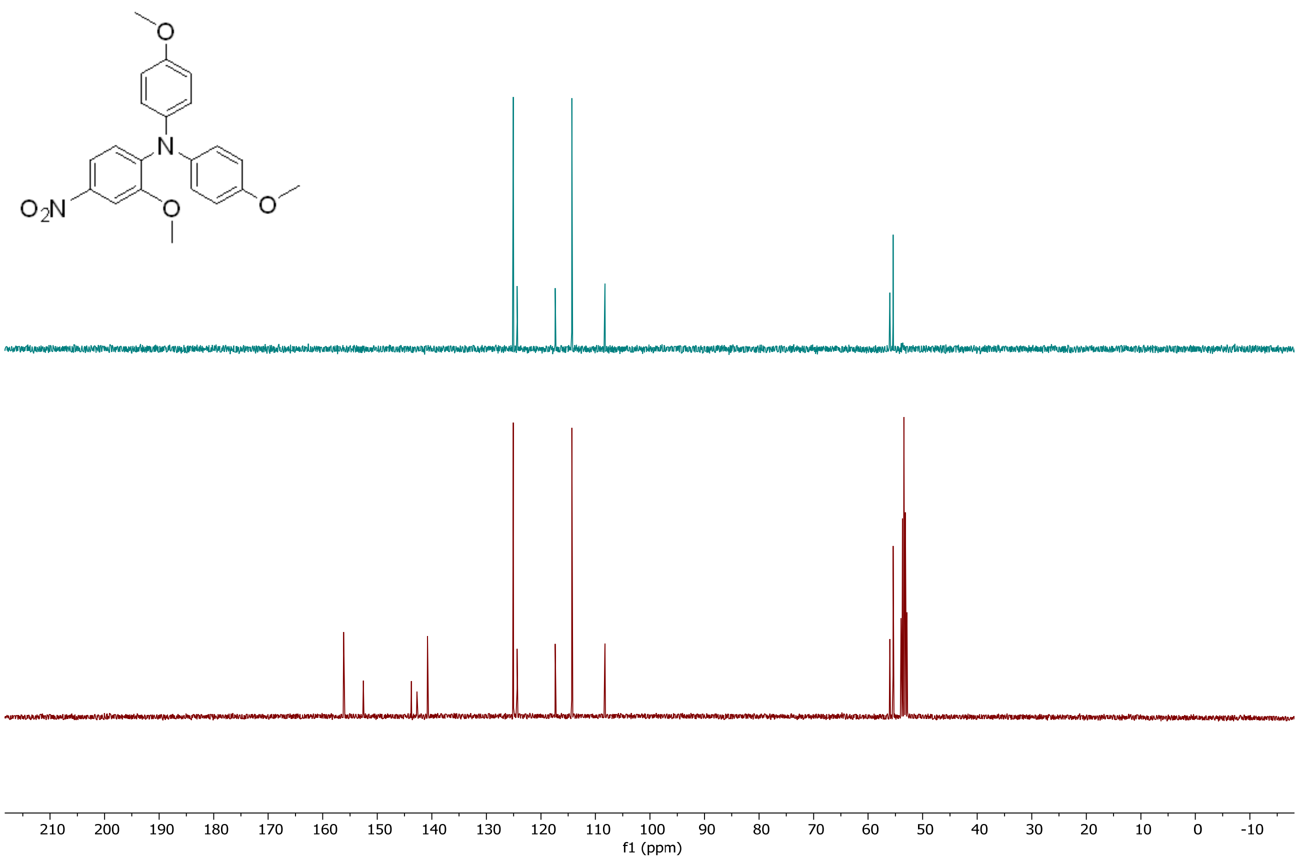

Fig. S42: Compound **18** ^{13}C-NMR (400 MHz, DCM-d_2): top (DEPT-90), bottom ^{13}C

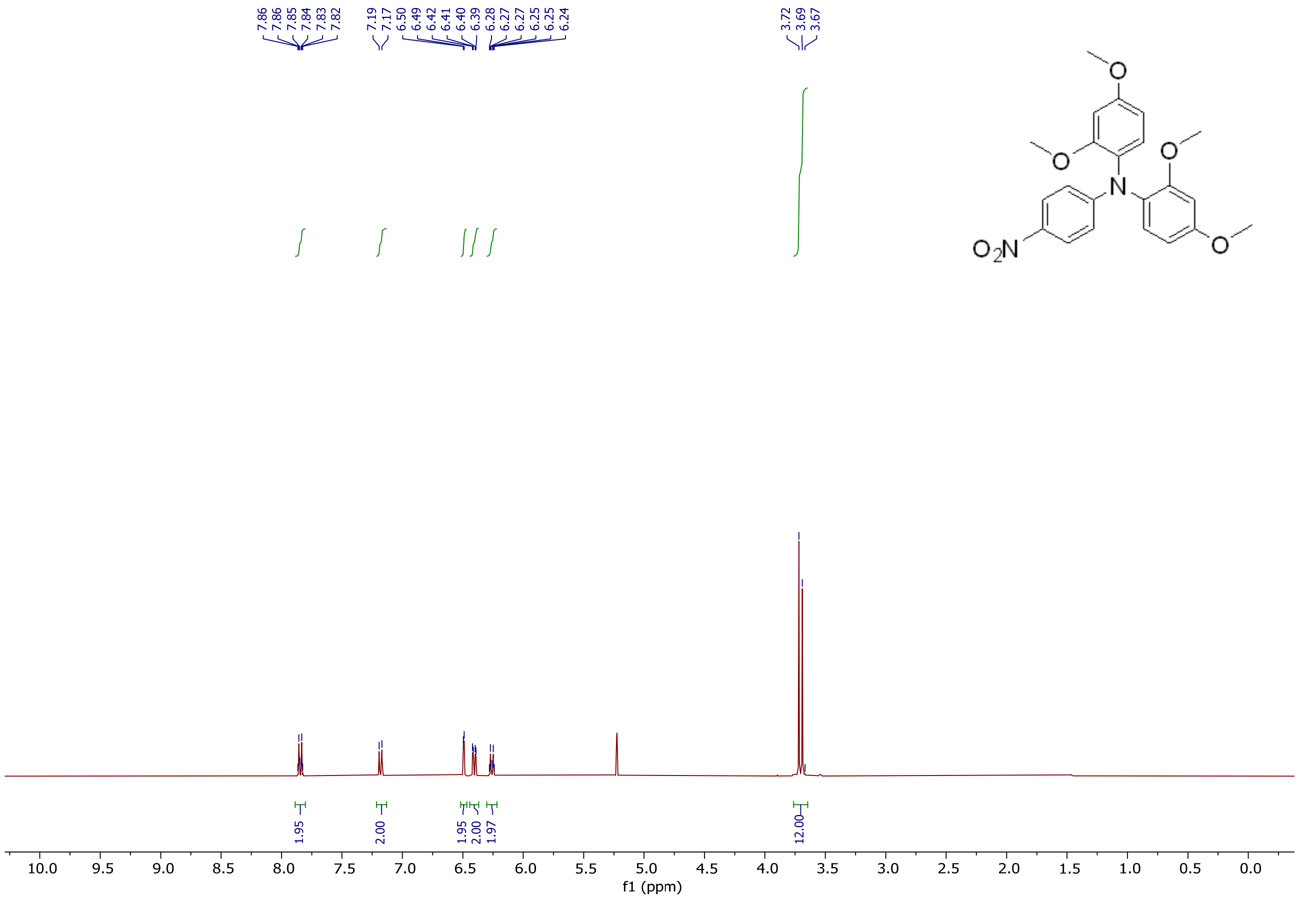

Fig. S43: Compound **19** ^{1}H-NMR (400 MHz, DCM-d_2).

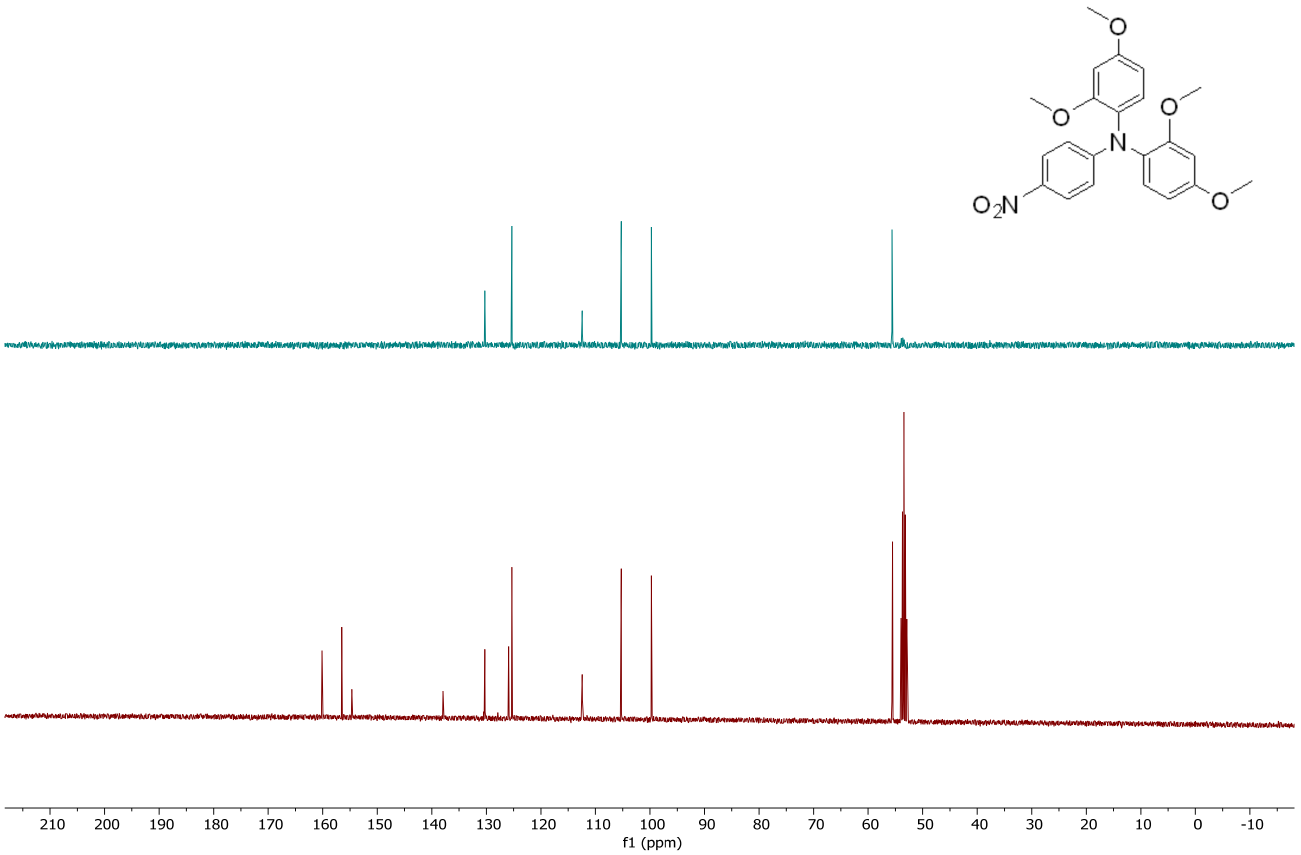

Fig. S44: Compound **19** ^{13}C-NMR (400 MHz, DCM-d$_2$): top (DEPT-90), bottom ^{13}C.

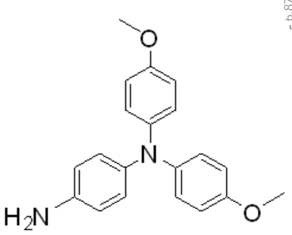

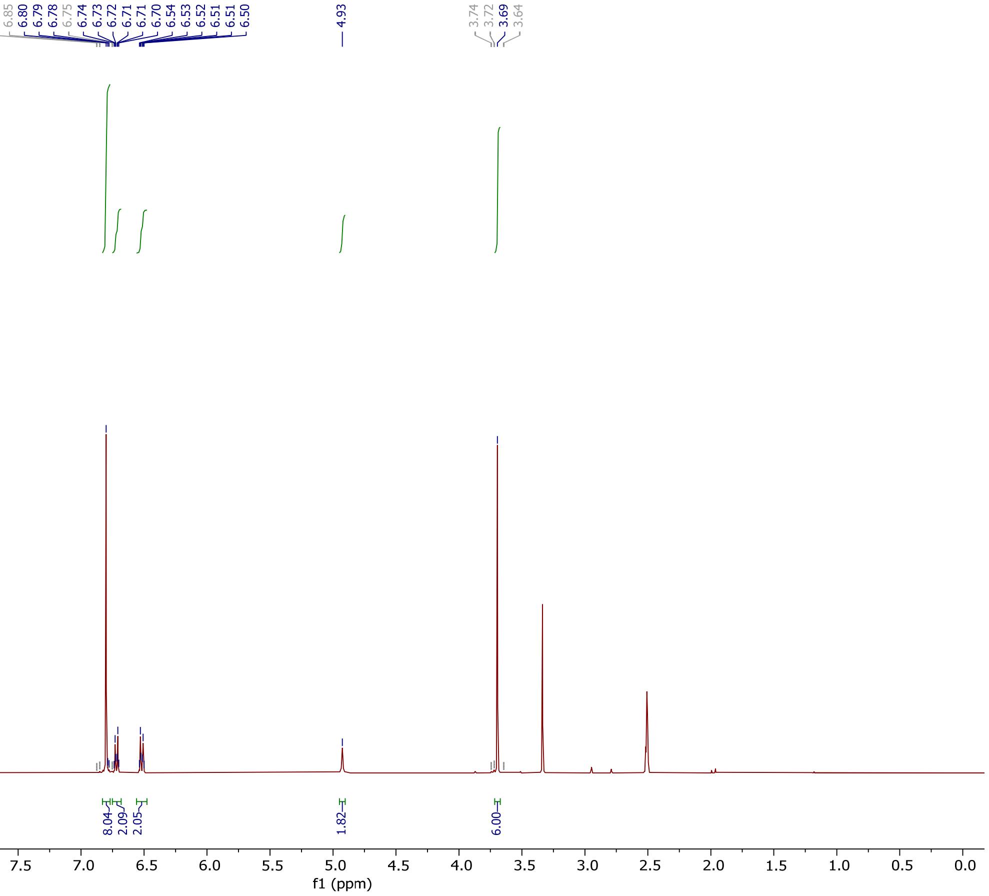

Fig. S45: Compound **20** ^{1}H-NMR (400 MHz, dmso-d$_6$).

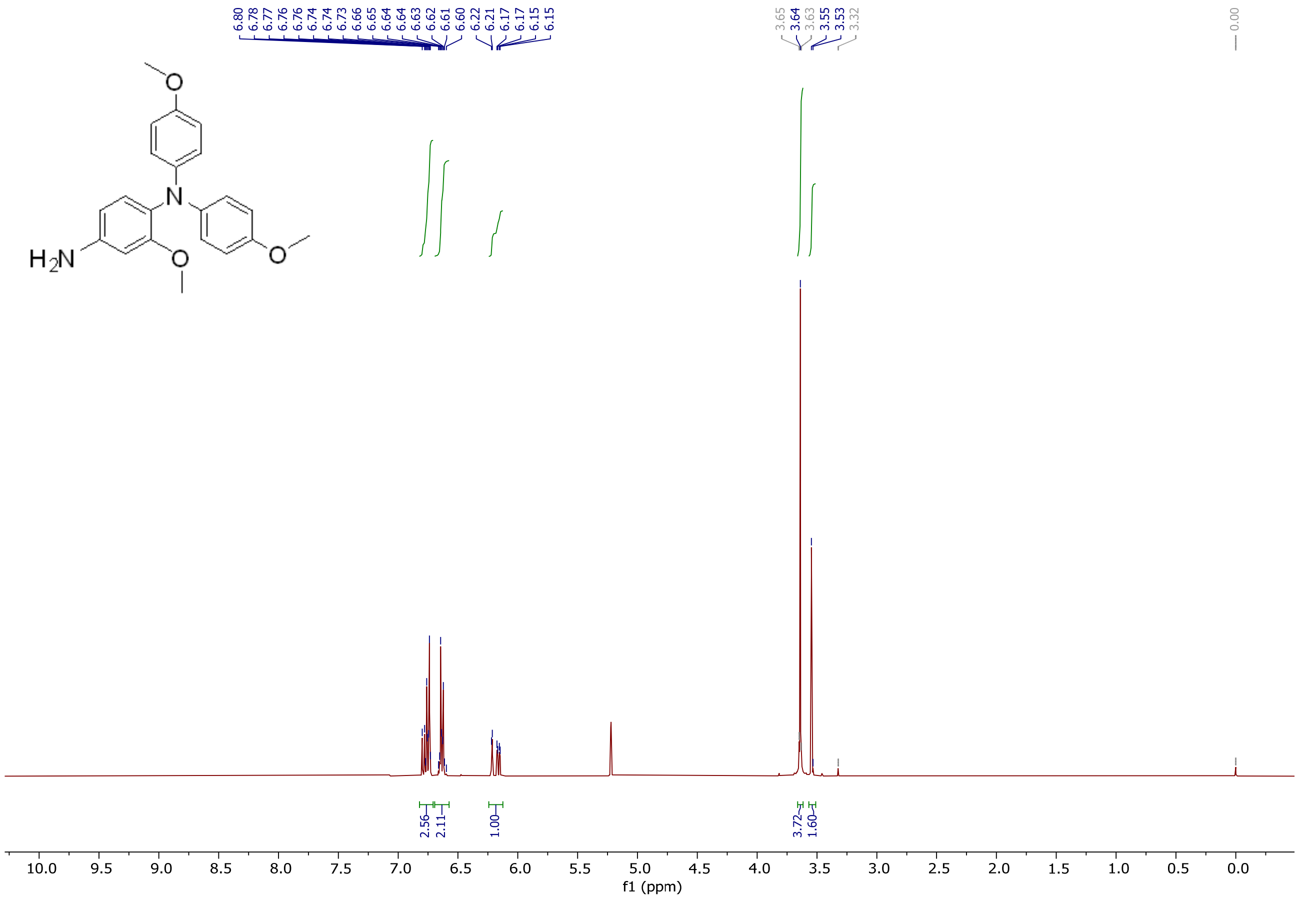

Fig. S46: Compound **21** ^{1}H-NMR (400 MHz, DCM-d_2).

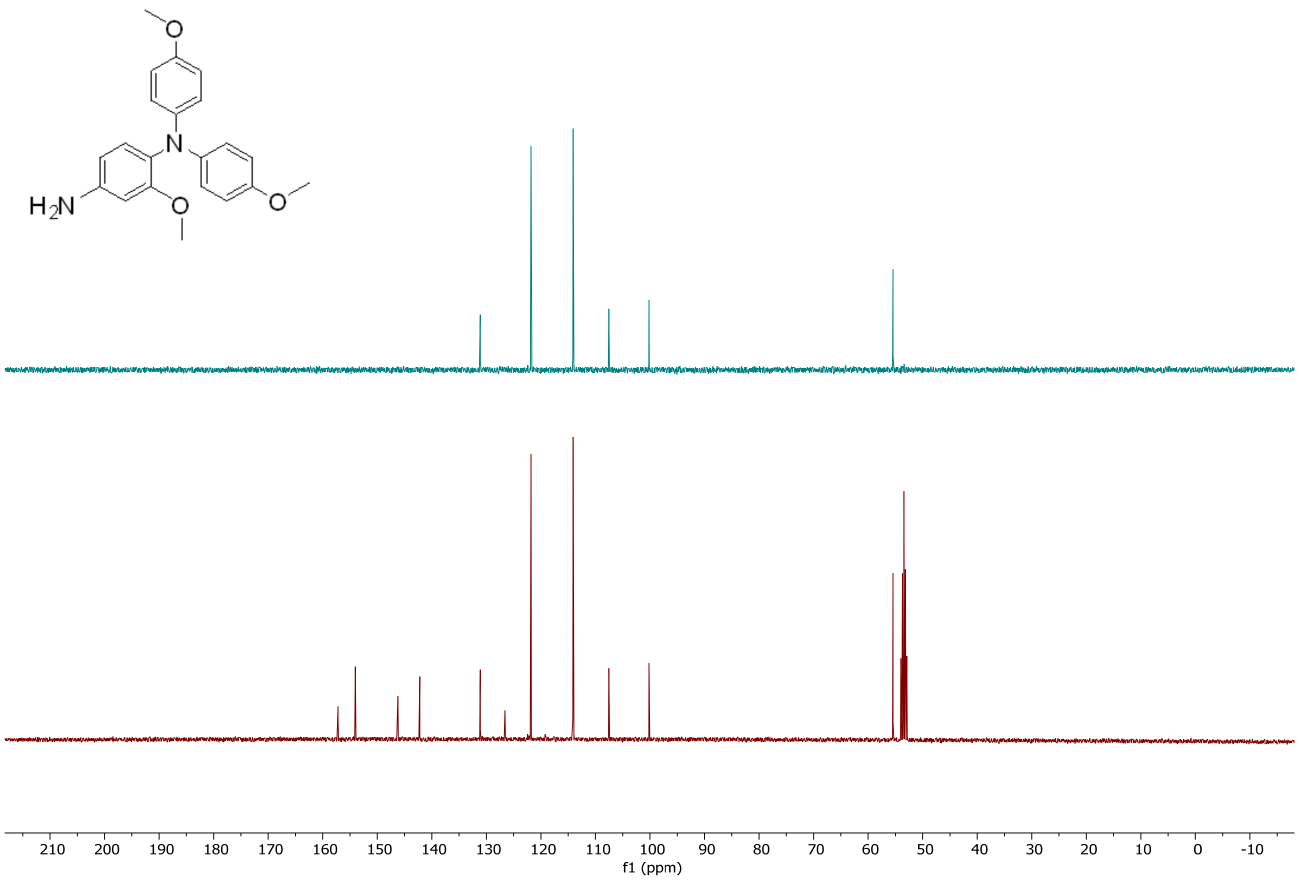

Fig. S47: Compound **21** ^{13}C-NMR (400 MHz, DCM-d$_2$): top (DEPT-90), bottom ^{13}C.

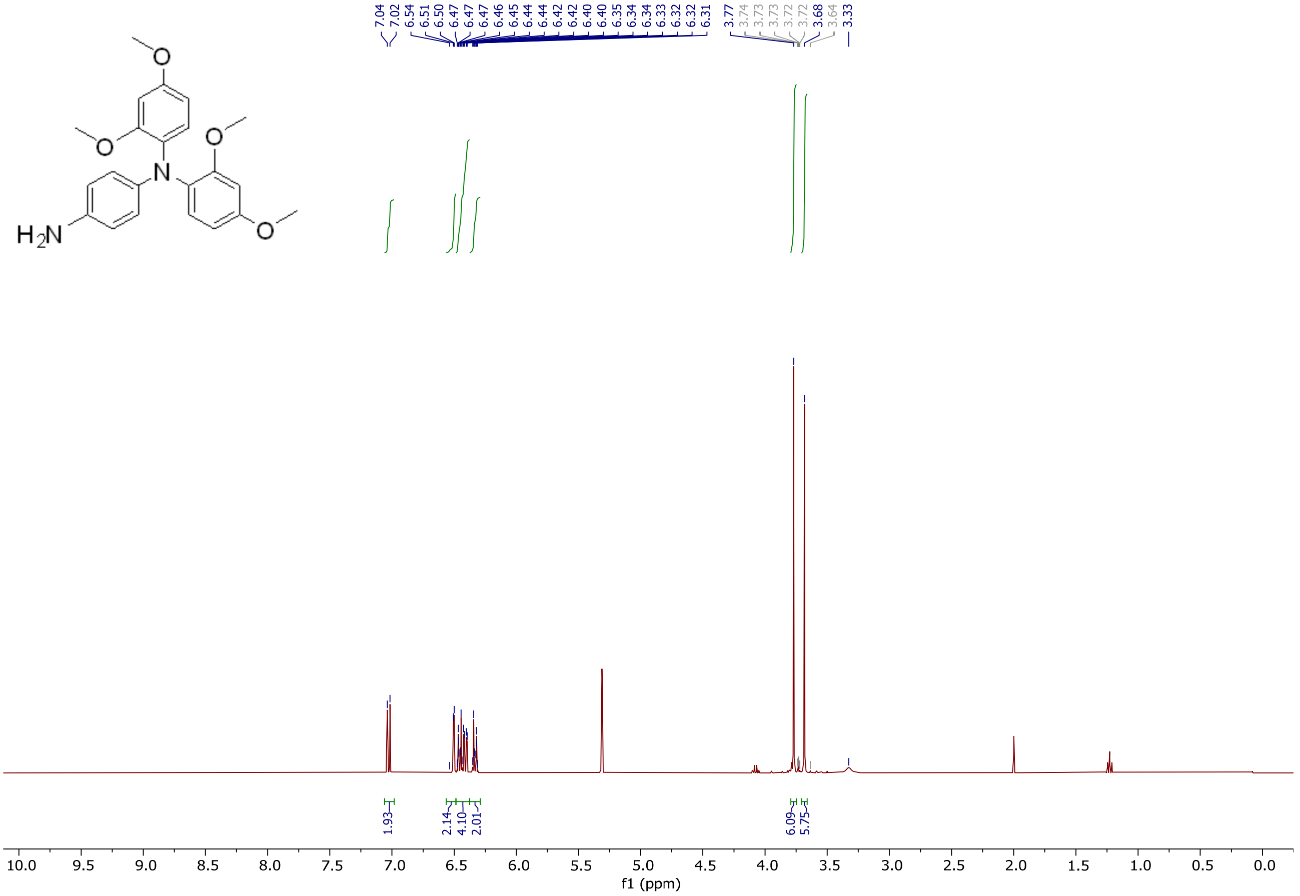

Fig. S48: Compound **22** ^{1}H-NMR (400 MHz, DCM-d_2).

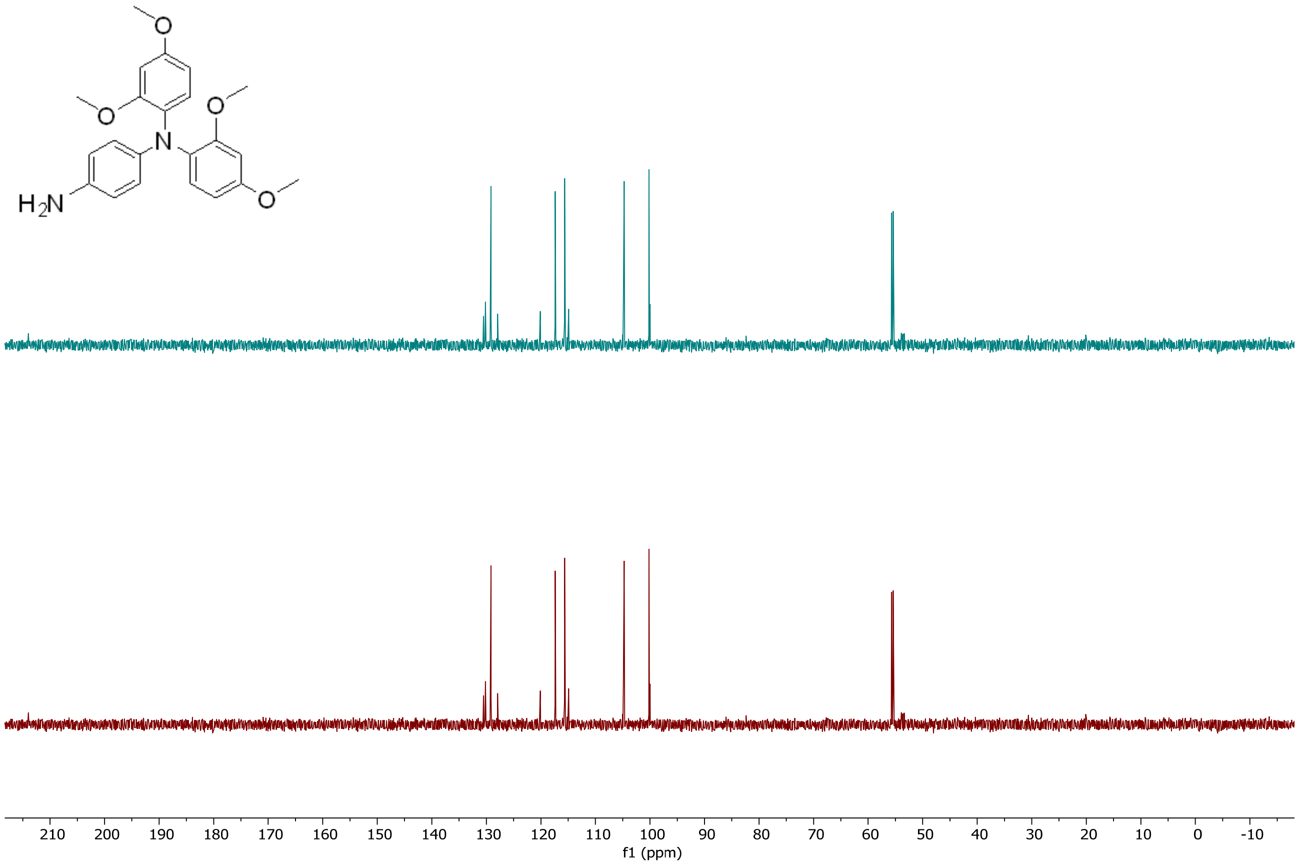

Fig. S49: Compound **22** ^{13}C-NMR (400 MHz, DCM-d$_2$): top (DEPT-90), bottom ^{13}C.

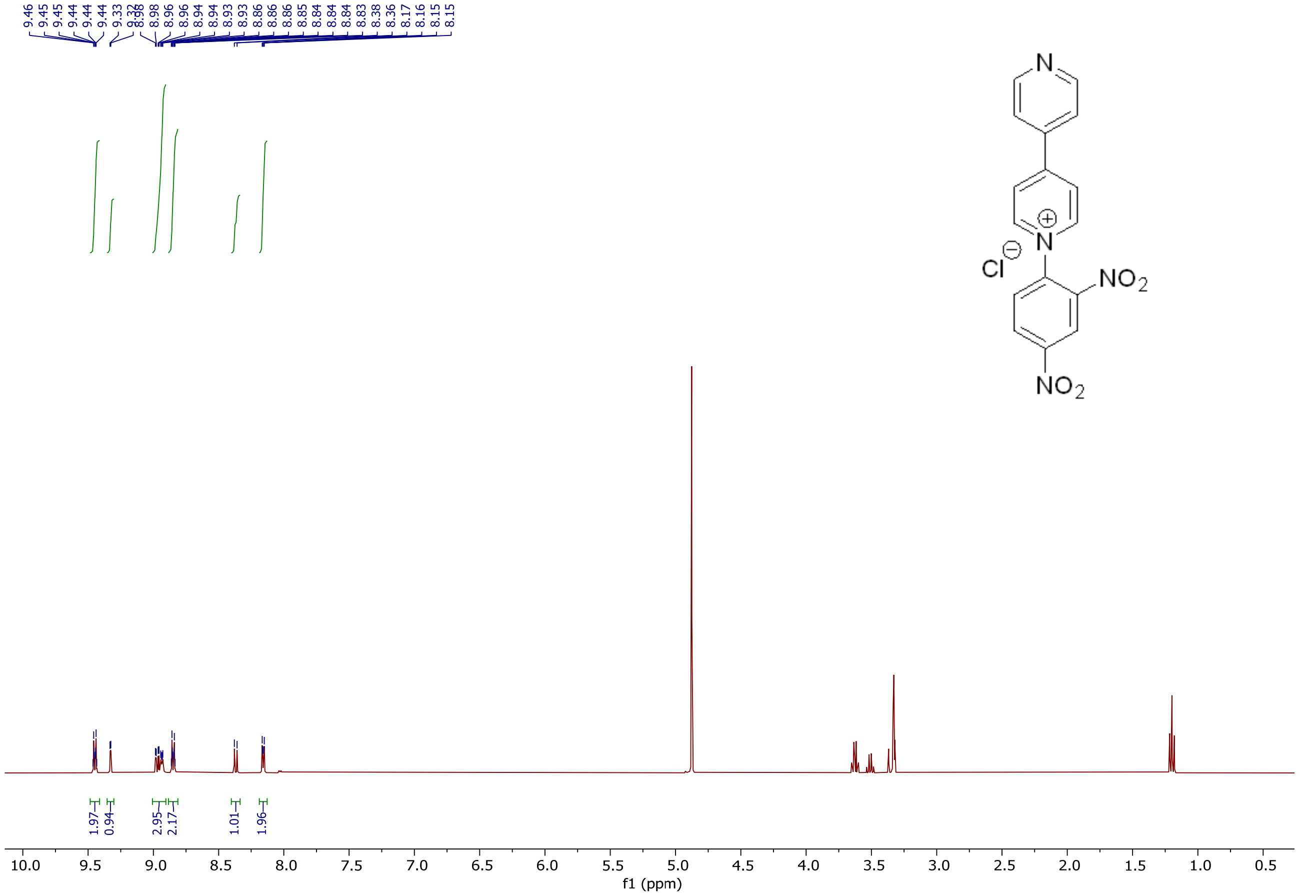

Fig. S50: Compound **23** ^{1}H-NMR (400 MHz, D_2O).

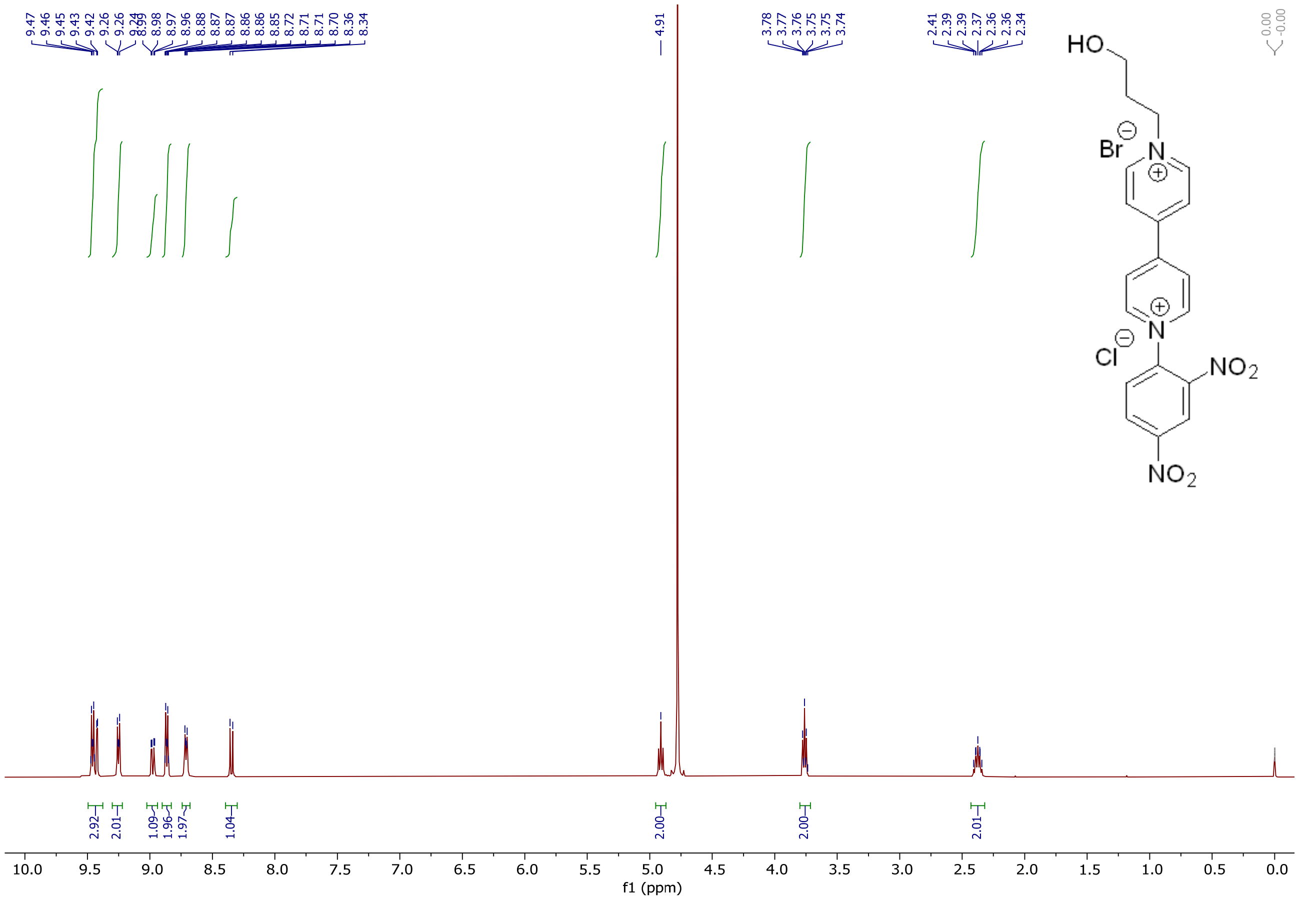

Fig. S51: Compound **24** ^{1}H-NMR (400 MHz, D_2O).

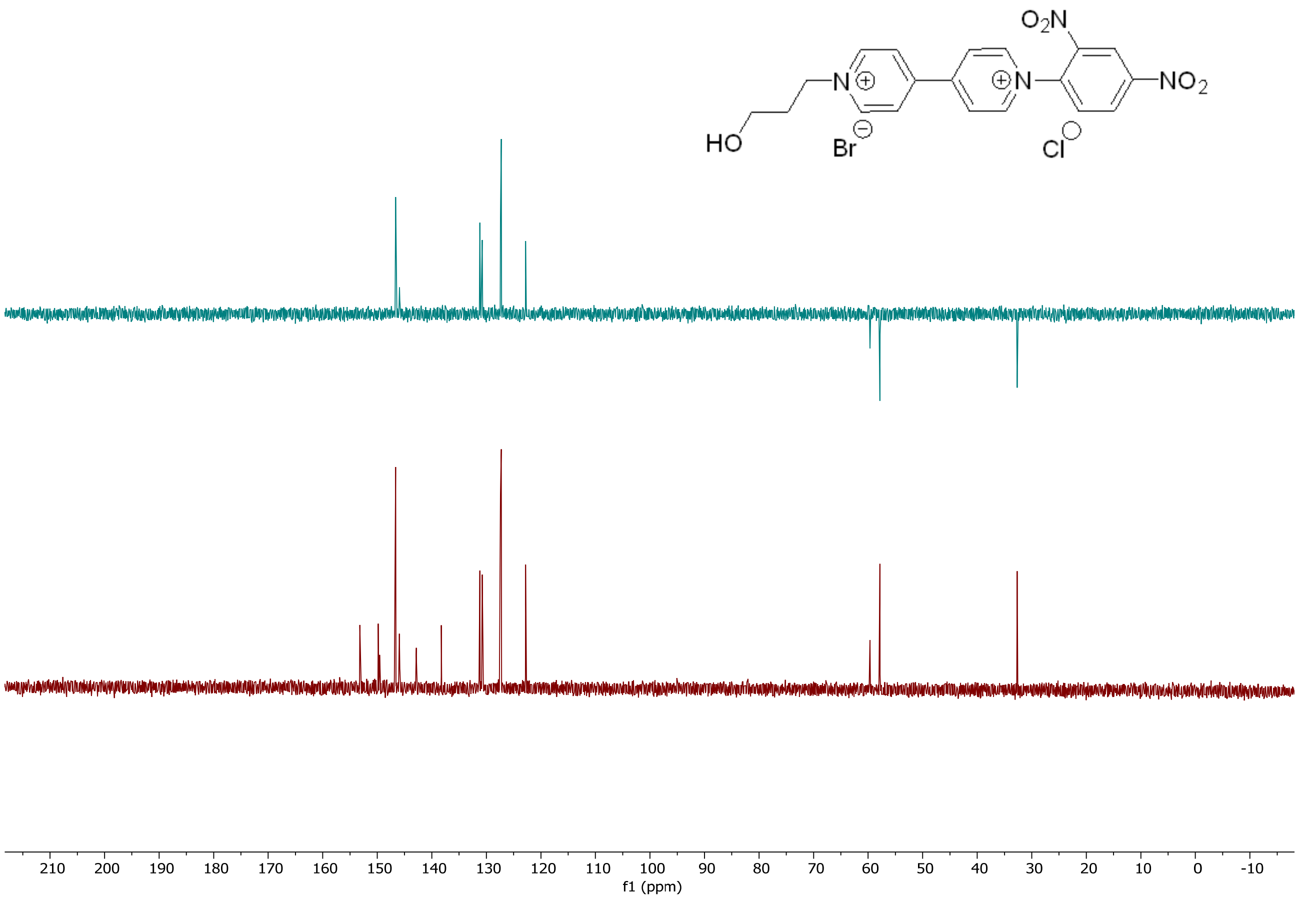

Fig. S52: Compound **24** ^{13}C-NMR (400 MHz, D_2O + TMSP-d_4): top (DEPT-90), bottom ^{13}C.

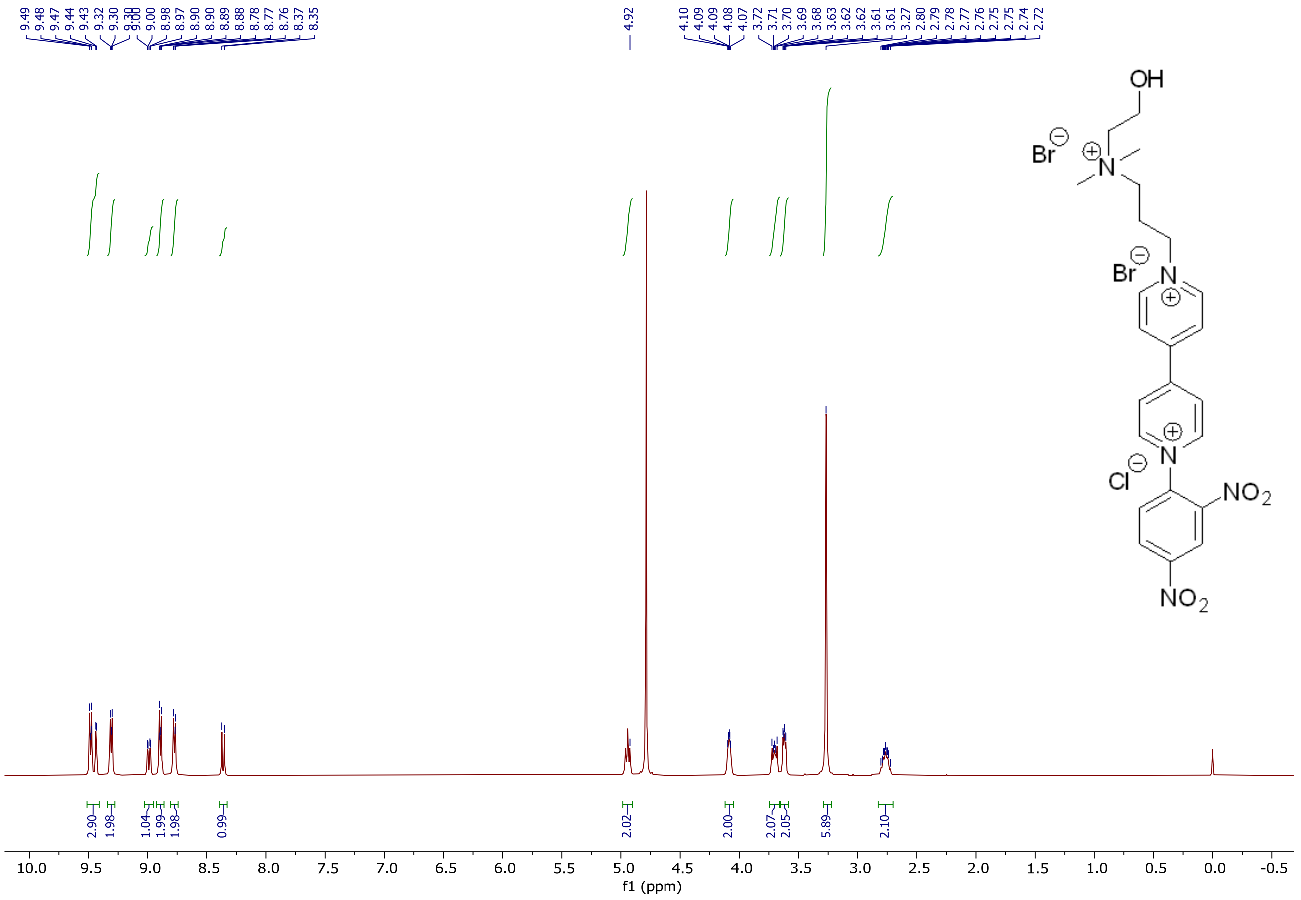

Fig. S53: Compound **25** ^{1}H-NMR (400 MHz, D_2O).

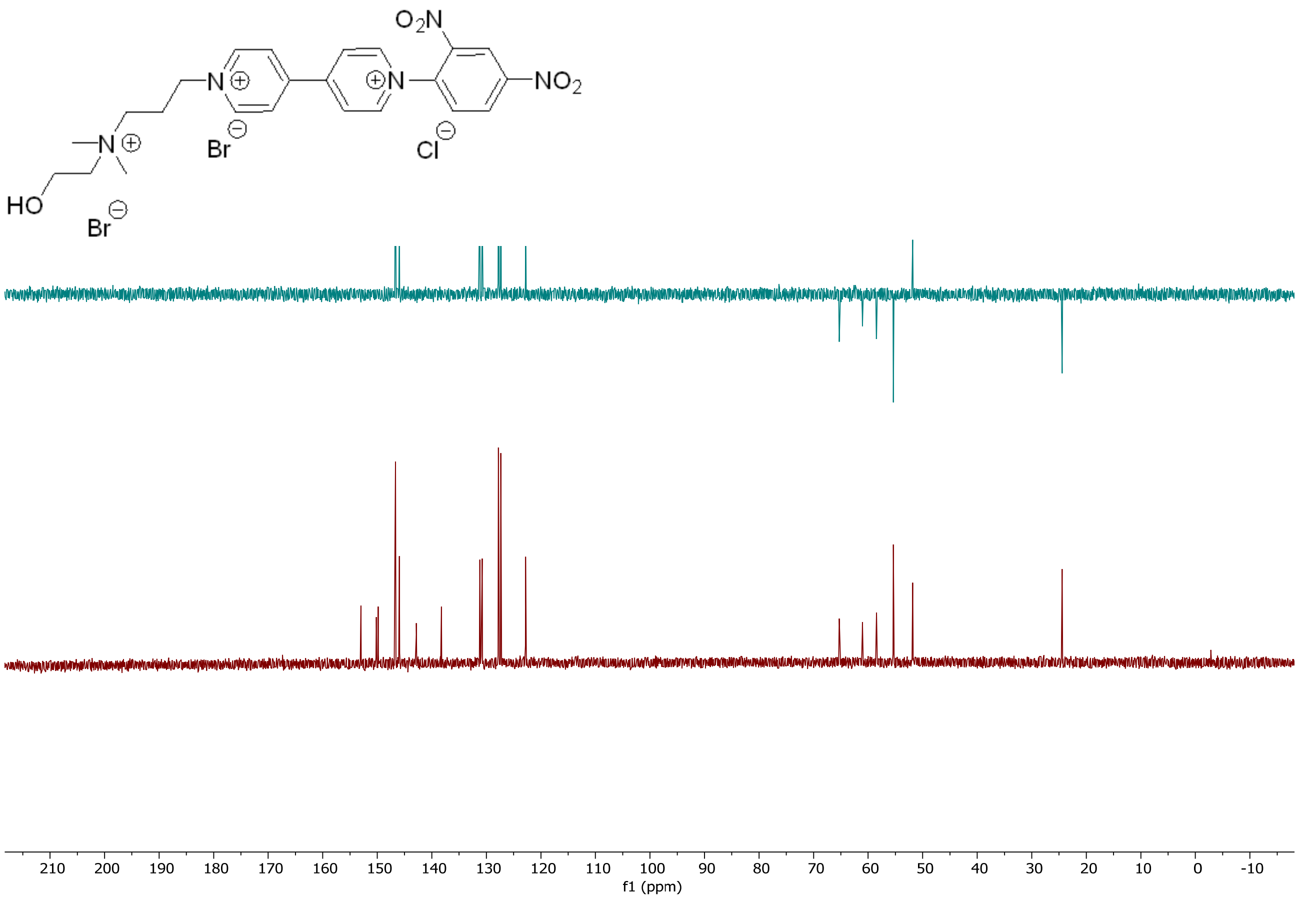

Fig. S54: Compound **25** ^{13}C-NMR (400 MHz, D_2O + TMSP-d_4): top (DEPT-90), bottom ^{13}C.

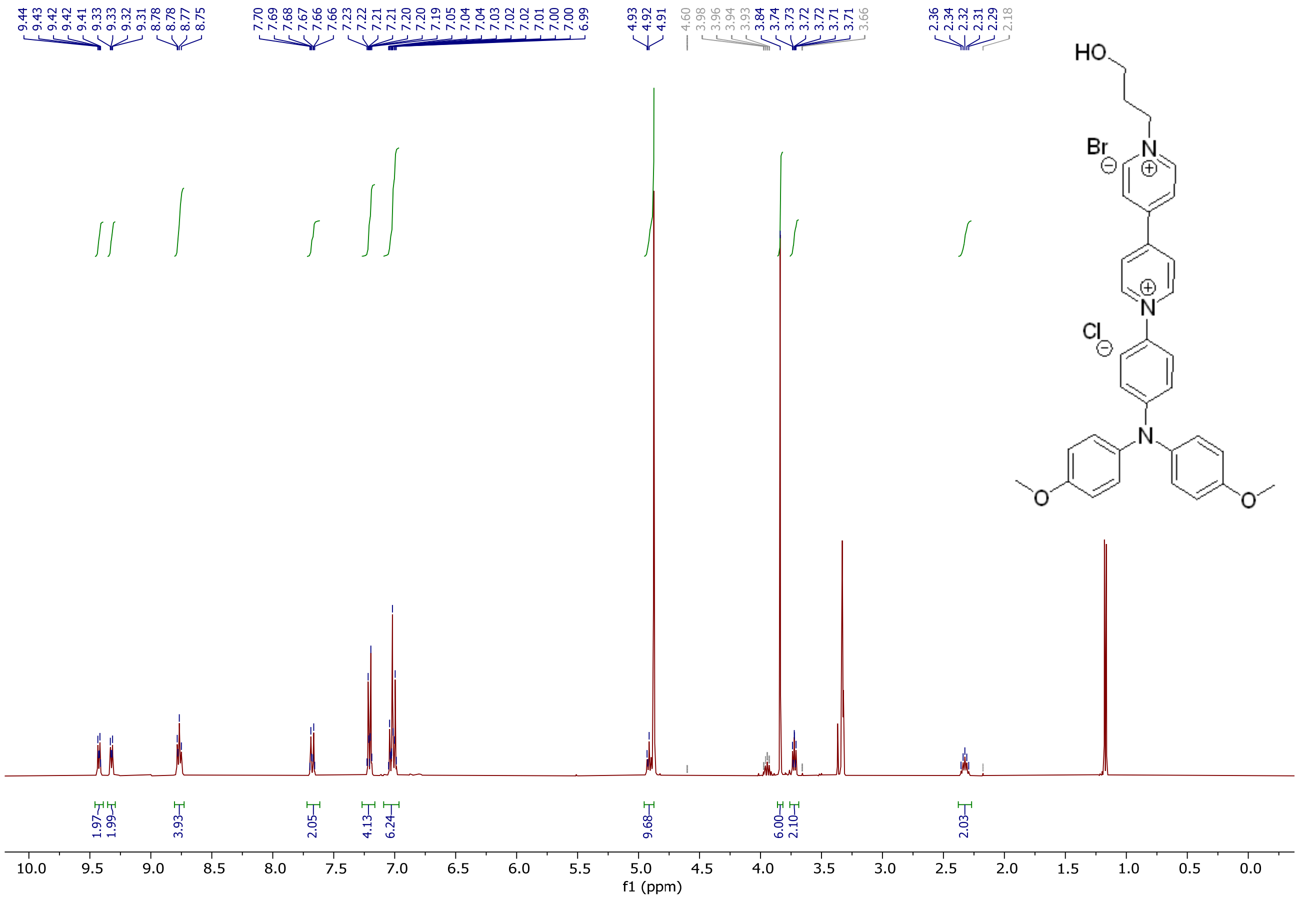

Fig. S55: Compound **7** ^{1}H-NMR (600 MHz, CD_3OD).

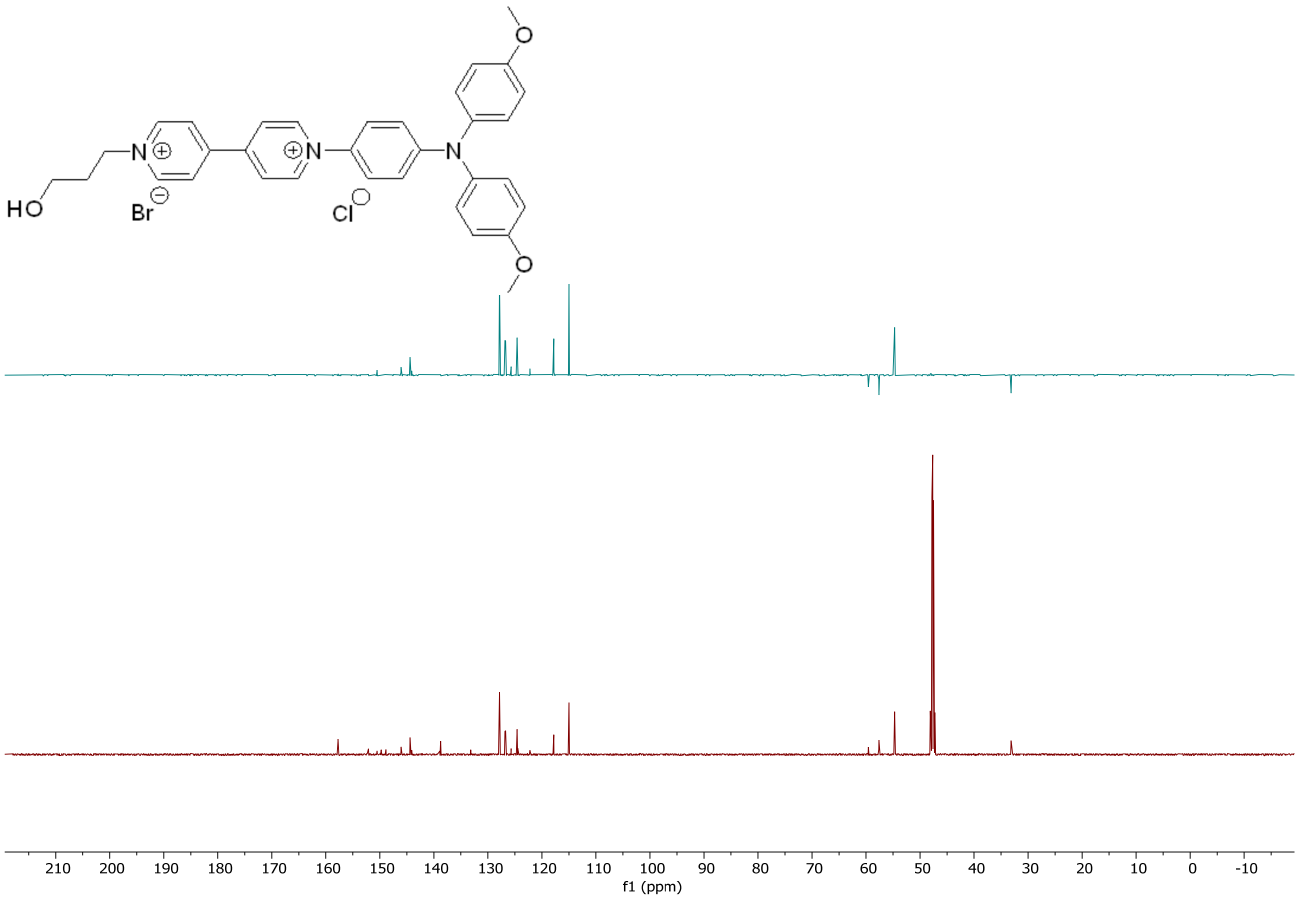

Fig. S56: Compound **7** ^{13}C-NMR (400 MHz, D_2O + TMSP-d_4): top (DEPT-90), bottom ^{13}C.

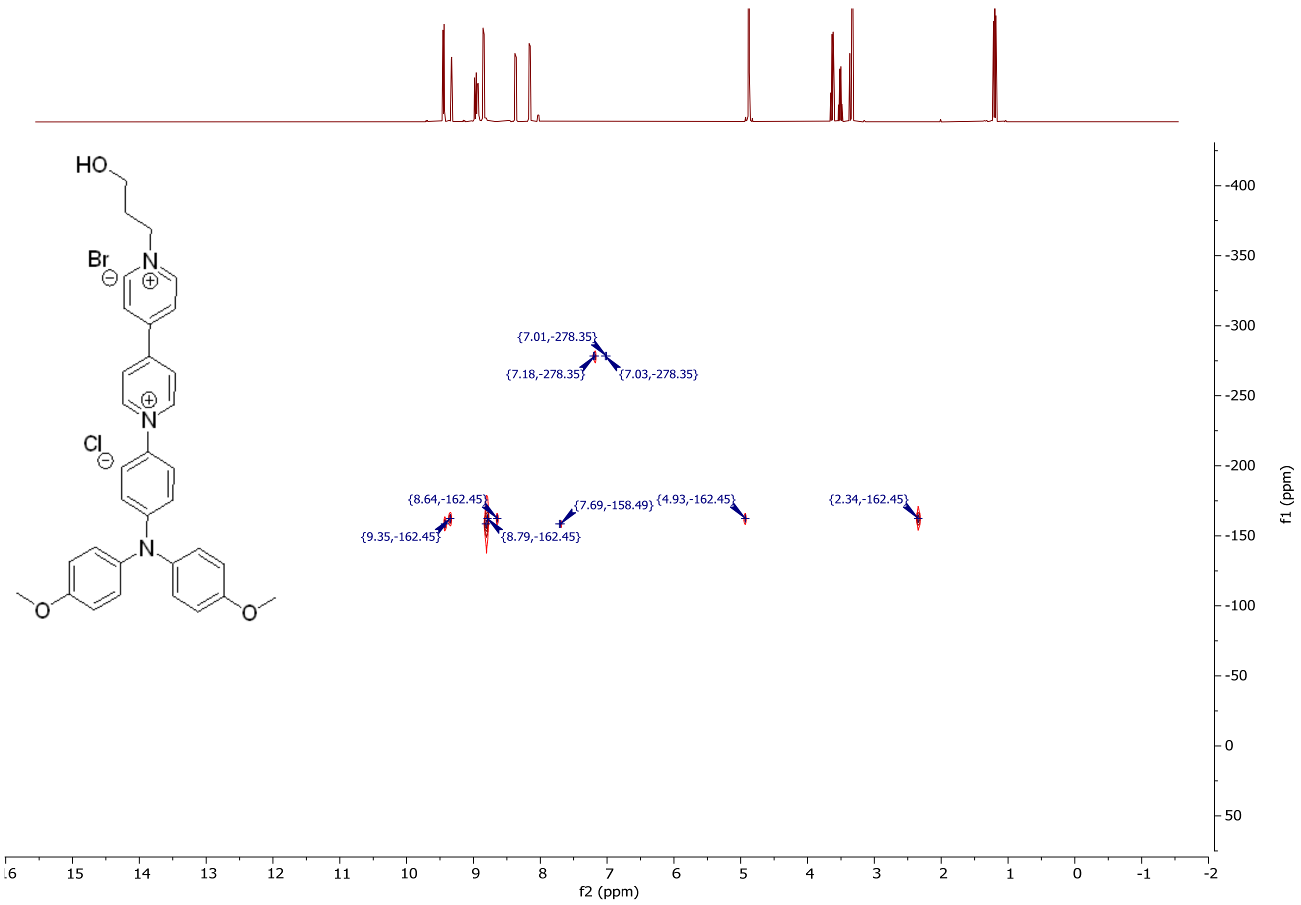

Fig. S57: Compound **7** ^{15}N-HMBC (600 MHz, CD_3OD).

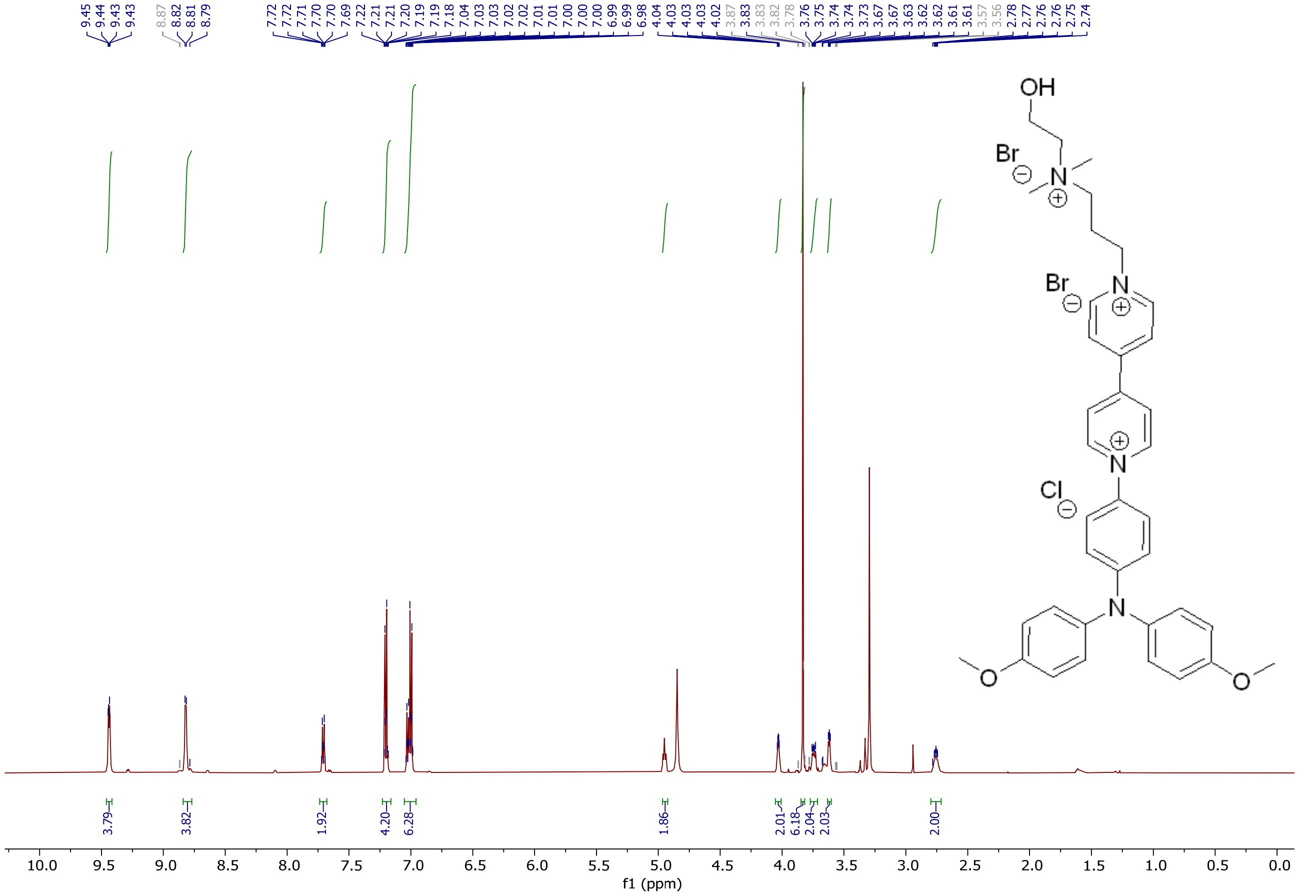

Fig. S58: Compound **8** ^{1}H-NMR (600 MHz, CD_3OD).

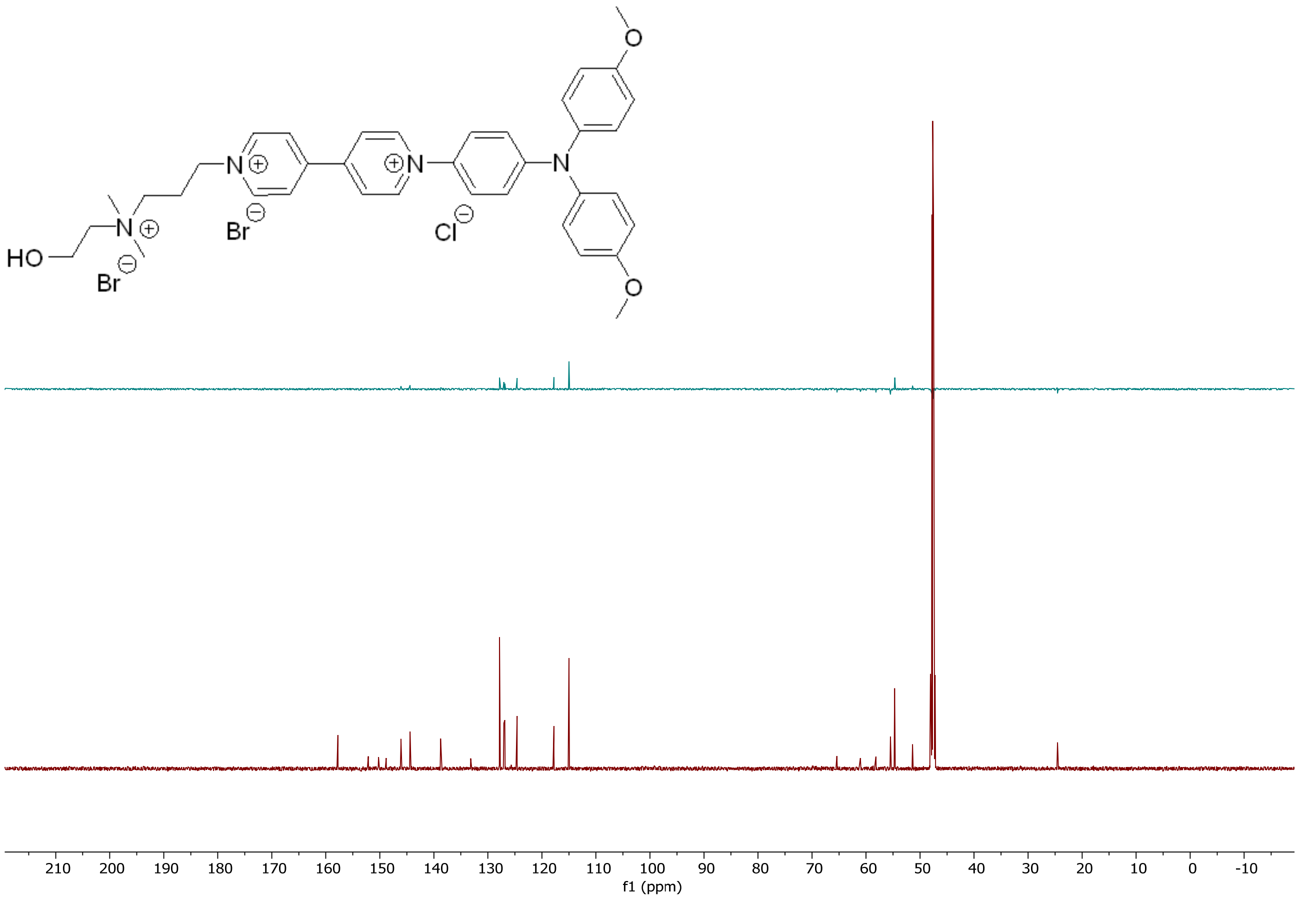

Fig. S59: Compound **8** ^{13}C-NMR (400 MHz, D_2O + TMSP-d_4): top (DEPT-90), bottom ^{13}C.

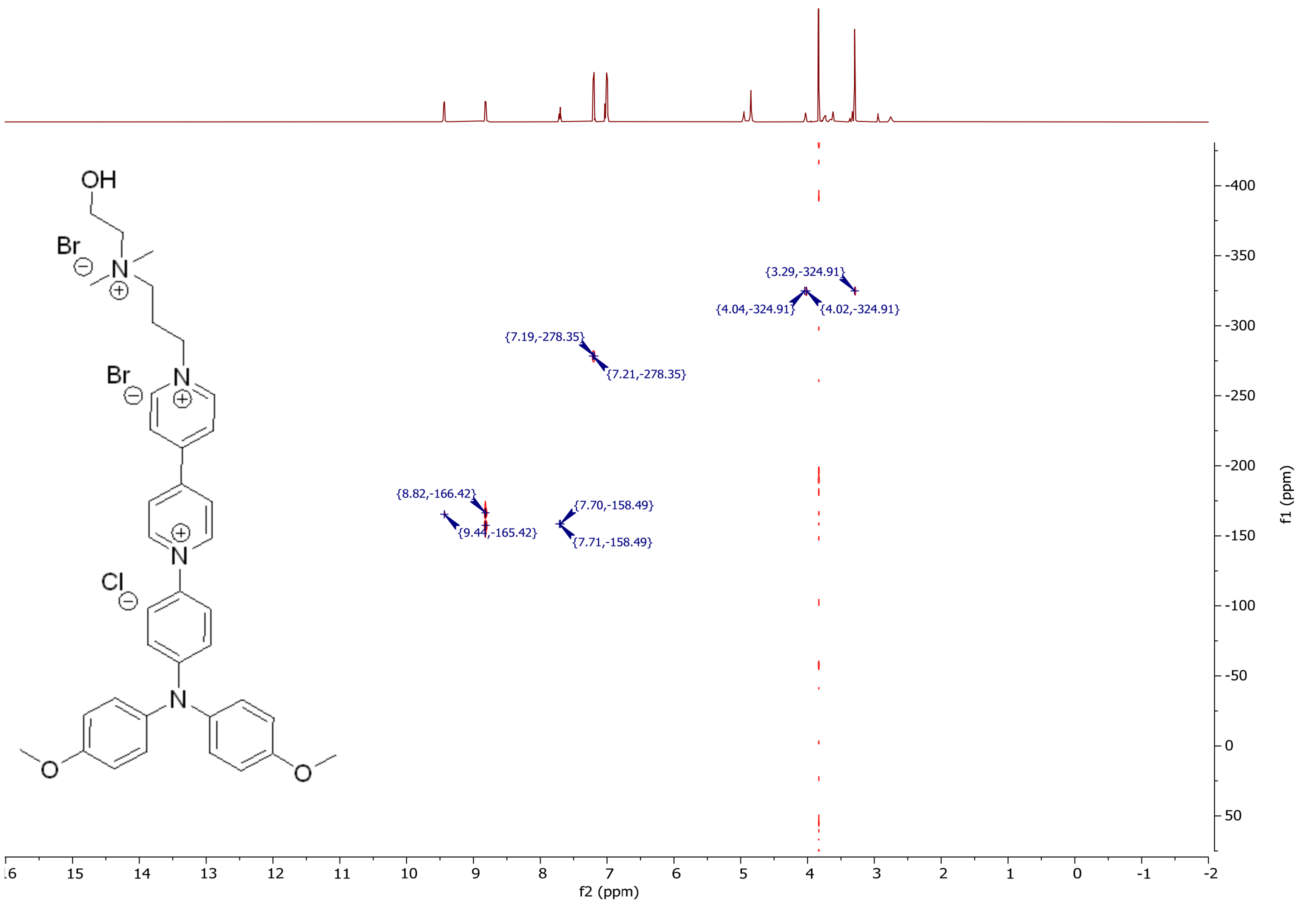

Fig. S60: Compound **8** ^{15}N-HMBC (600 MHz, CD_3OD).

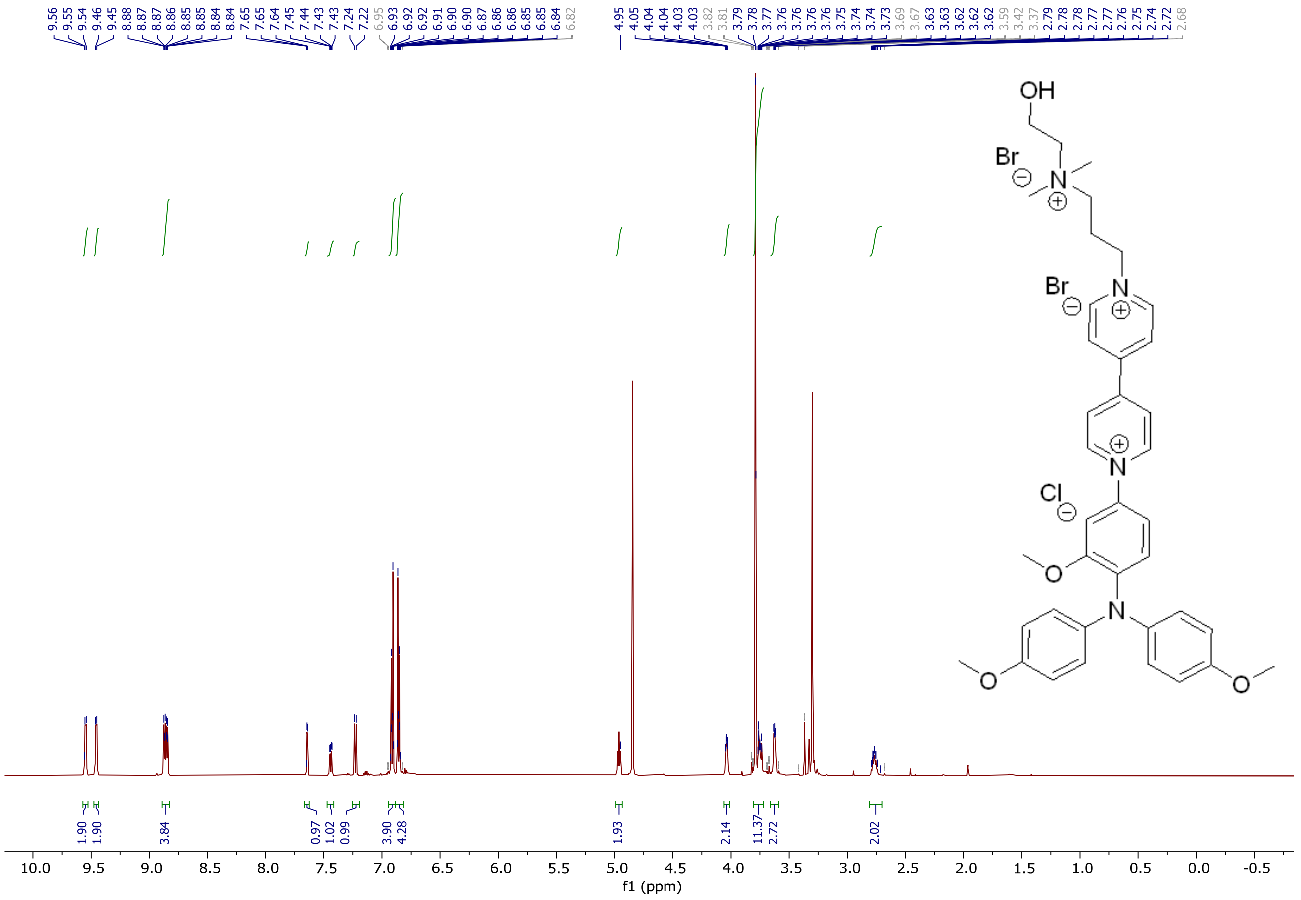

Fig. S61: Compound **9** ^{1}H-NMR (600 MHz, CD_3OD).

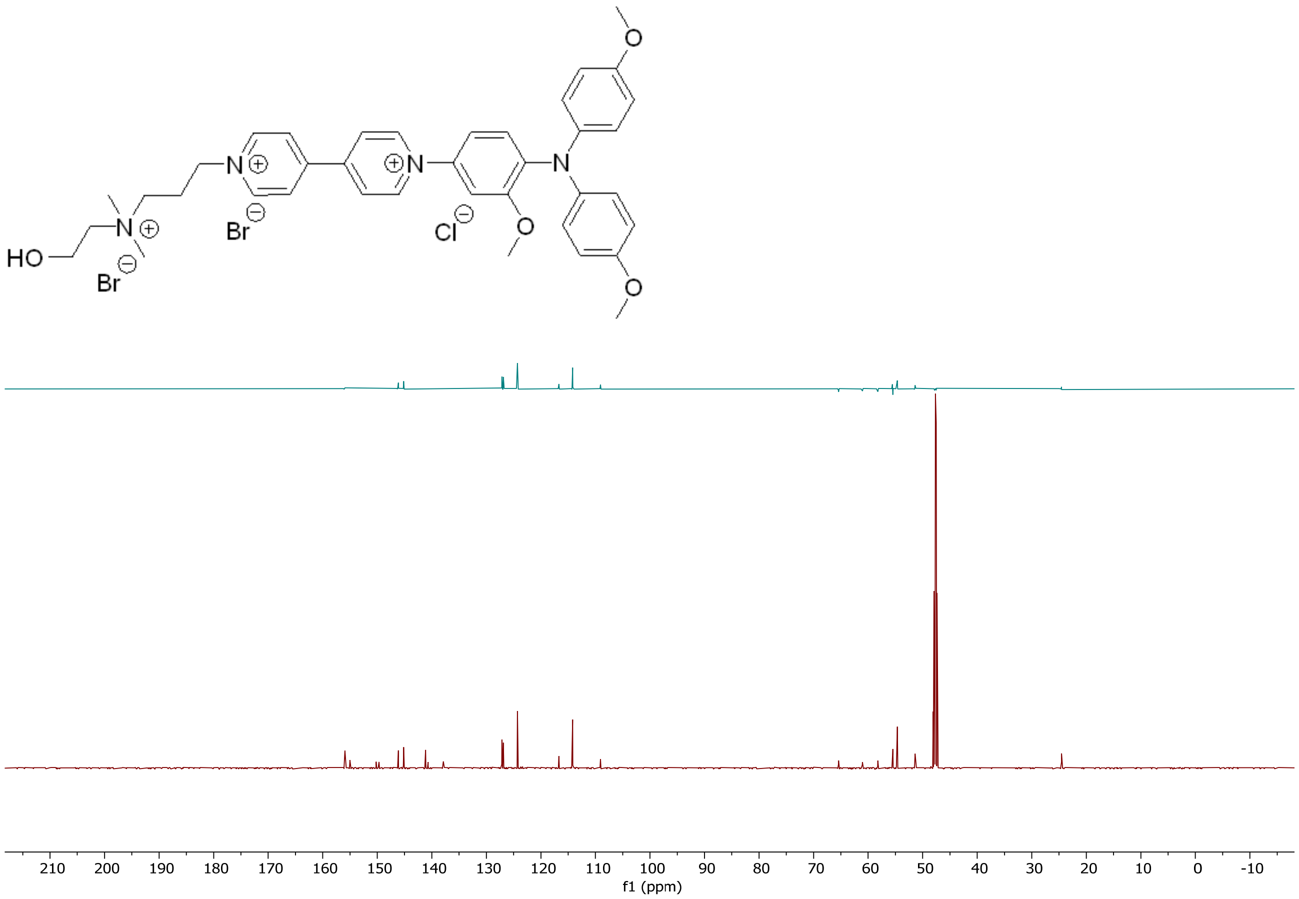

Fig. S62: Compound **9** ^{13}C-NMR (600 MHz, CD_3OD): top (DEPT-90), bottom ^{13}C.

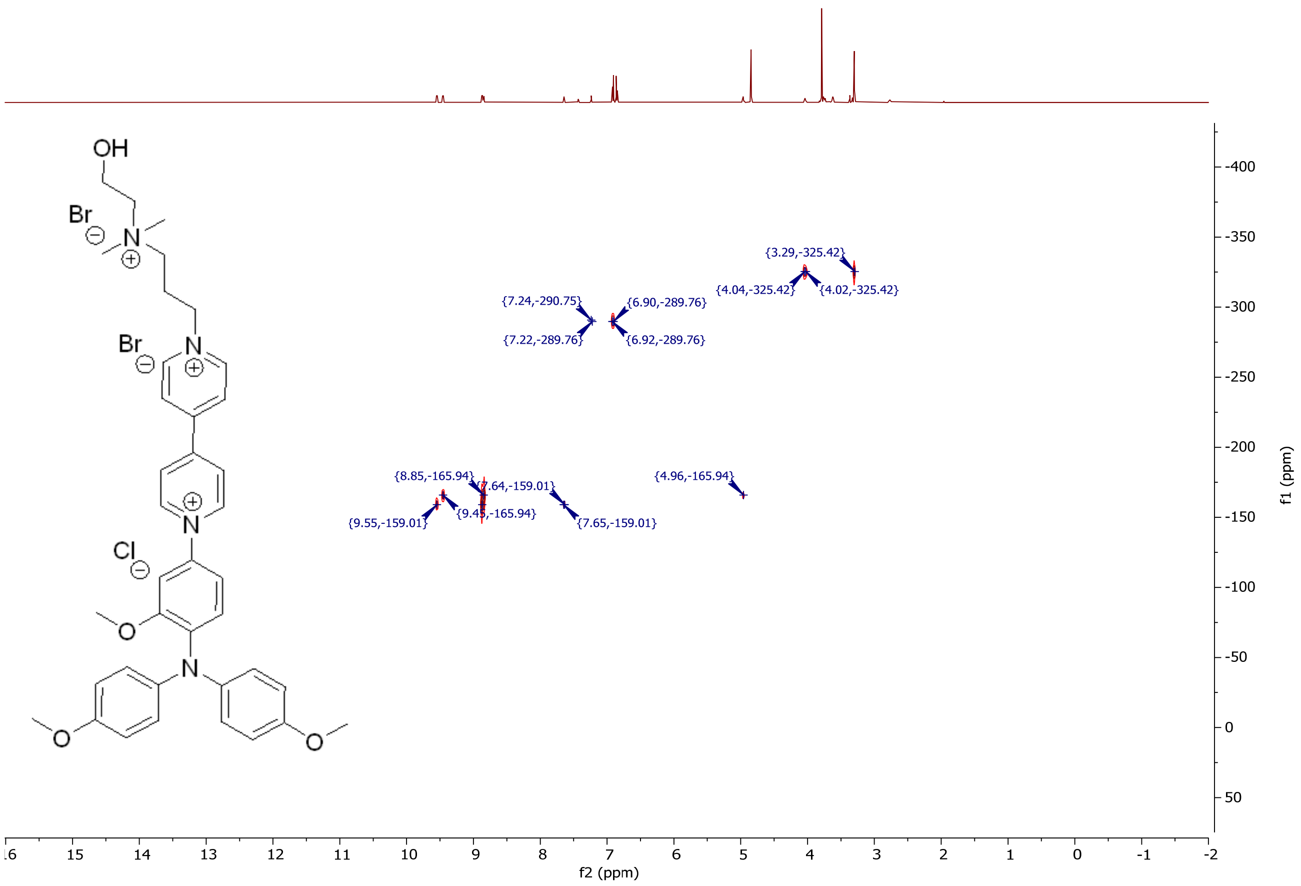

Fig. S63: Compound **9** ^{15}N-HMBC (600 MHz, CD_3OD).

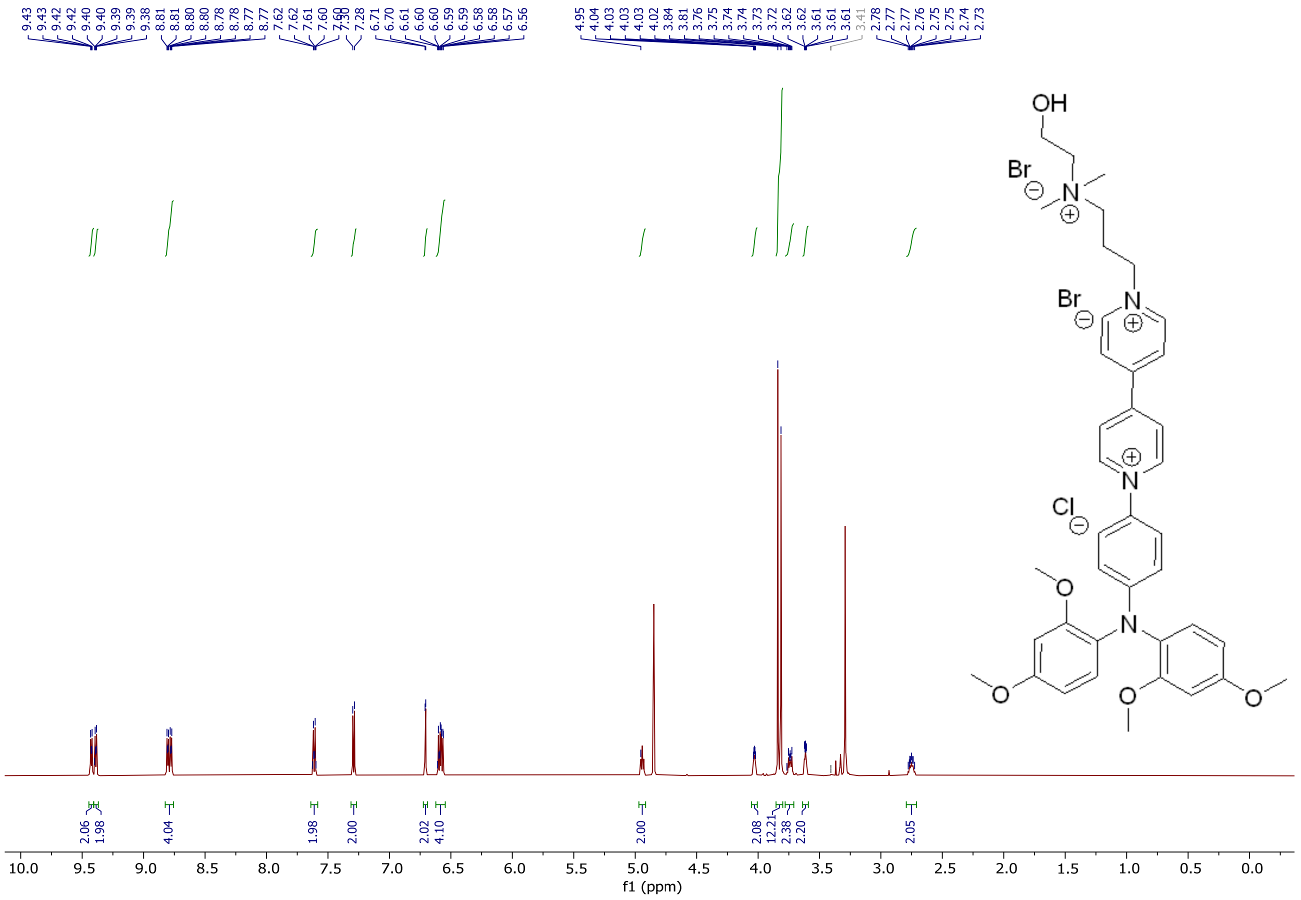

Fig. S64: Compound **10** ^{1}H-NMR (600 MHz, CD_3OD).

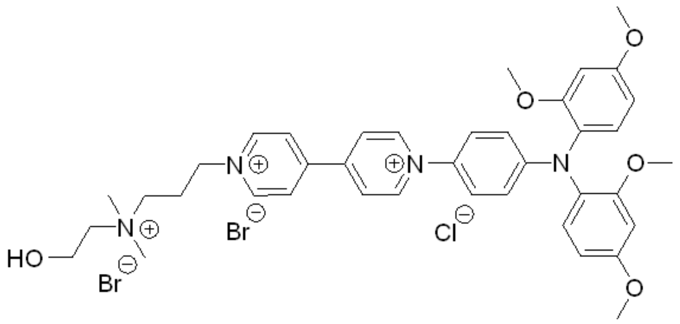

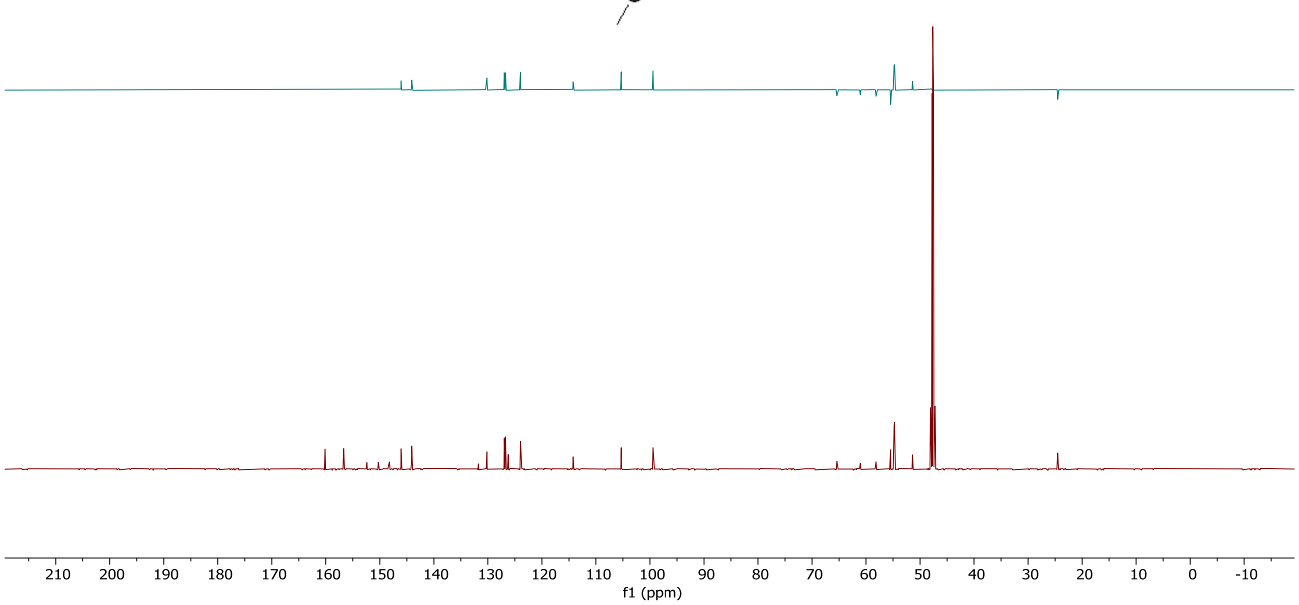

Fig. S65: Compound **10** ^{13}C-NMR (600 MHz, CD_3OD): top (DEPT-90), bottom ^{13}C.

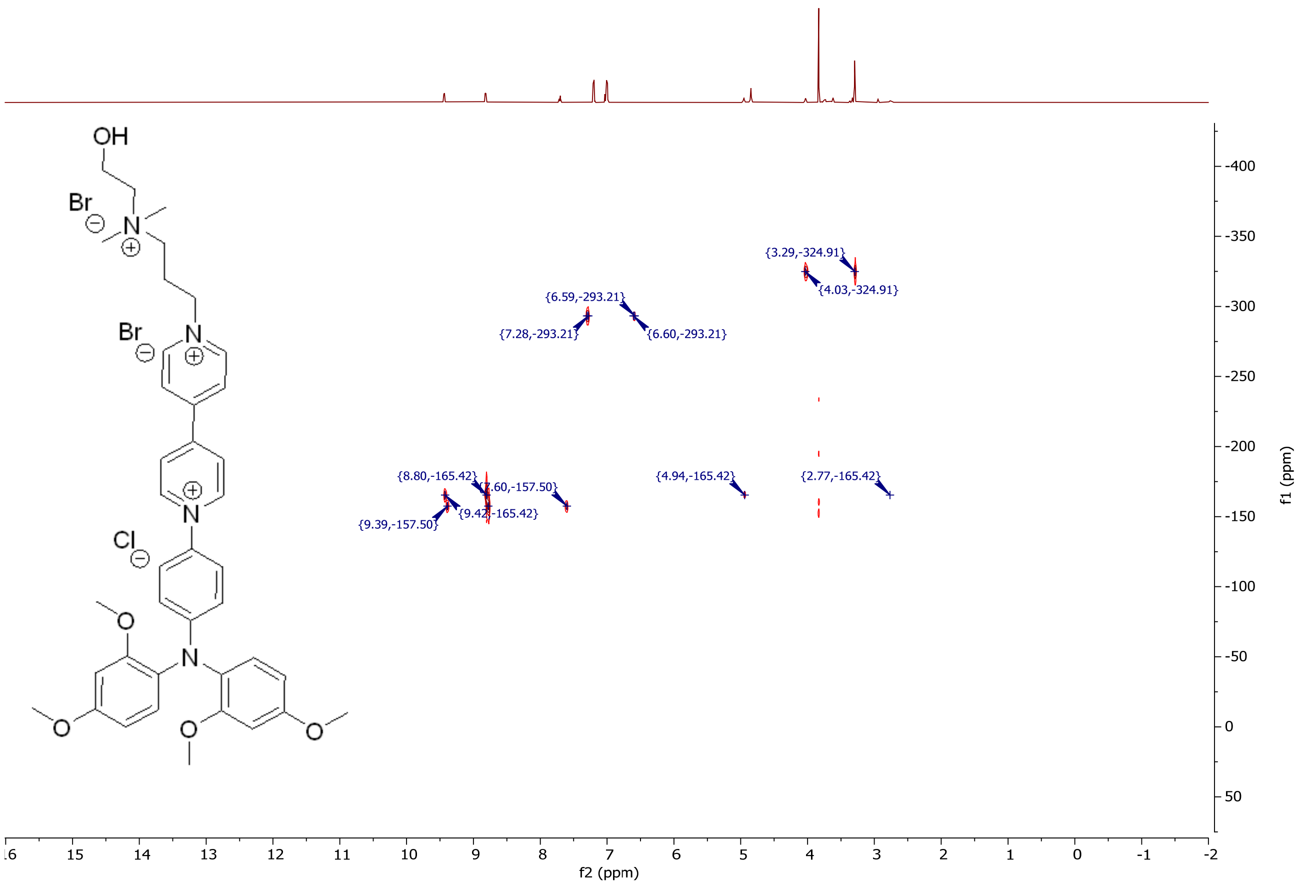

Fig. S66: Compound **10** ^{15}N-HMBC (600 MHz, CD_3OD).

References

(1) C. Caianiello, L.F. Arenas, L. F., T. Turek, T., R. Wilhelm,. *Batt. Supercaps* **2023**, 6 (1), e202200355. DOI: 10.1002/batt.202200355.

(2) F.A Gonçalves, J. Kestin, The Viscosity of NaCl and KCl Solutions in the Range 25–50°C. *Ber. Bunsenges Phys. Chem.* **1977**, 81 (11), 1156–1161. DOI: 10.1002/bbpc.19770811108.

(3) Density of KCl(aq). https://advancedthermo.com/electrolytes/density_KCl.html (accessed 2024-07-06).

(4) H. Zhao, X. Li, L. Li, R. Wang, *Small*. **2015**, 11 (30), 3642–3647. DOI: 10.1002/smll.201500658.

(5) E. S. Beh, D. de Porcellinis, R. L. Gracia, K. T. Xia, R. G. Gordon, M. J. Aziz, *ACS Energy Lett.* **2017**, 2 (3), 639–644. DOI: 10.1021/acsenergylett.7b00019.

(6) H. An, W. Park, H. Shin, D. Y., *EcoMat* **2024**, 6, e12486. DOI: 10.1002/eom2.12486

(7) N. Sinha, T. T. Y. Tan, E. Peris, F. E. Hahn, *Angew. Chem. Int. Ed*. **2017**, 56 (26), 7393–7397. DOI: 10.1002/anie.201702637.

(8) G. N. Lim, S. Hedström, K. A. Jung, P. A. D. Smith, V. S. Batista, F. D'Souza, A. van der Est, A.; P. K. Poddutoori, *J. Phys. Chem. C* **2017**, 121 (27), 14484–14497. DOI: 10.1021/acs.jpcc.7b04197.

(9) M. Zhou, L. Mao, Y. F. Niu, X. L. Zhao, X. Shi, H. B. Yang, *Chin. Chem. Lett.* **2022**, 33 (4), 1870–1874. DOI: 10.1016/j.cclet.2021.11.054.

(10) US2013193840A1.

(11) C.-M. Hung, J.-T. Lin, Y.-H. Yang, Y.-C. Liu, M.-W. Gu, T.-C. Chou, S.-F. Wang, Z.-Q. Chen, C.-C. Wu, L.-C. Chen, C.-C. Hsu, C.-H. Chen, C.-W. Chiu, H.-C. Chen, P.-T. Chou, *JACS Au* **2022**, 2, 160. DOI: 10.1021/jacsau.2c00160.

(12) M. Yano, Y. Ishida, K. Aoyama, M. Tatsumi, K. Sato, D. Shiomi, A. Ichimura, T. Takui, Synthesis and Electronic Properties of Tetraaryl p- and m-Phenylenediamines. *Synth. Met.* **2003**, 137 (1-3), 1275–1276. DOI: 10.1016/S0379-6779(02)01137-2.

(13) E. Moulin, F. Niess, M. Maaloum, E. Buhler, I. Nyrkova, N. Giuseppone, The hierarchical self-assembly of charge nanocarriers: a highly cooperative process promoted by visible light. *Angew. Chem. Int. Ed.* **2010**, 49 (39), 6974–6978. DOI: 10.1002/anie.201001833.

(14) R. Ye, Q. Cui, C. Yao, R. Liu, L. Li, Tunable fluorescence behaviors of a supramolecular system based on a fluorene derivative and cucurbit8uril and its application for ATP sensing. *Phys. Chem. Chem. Phys.* **2017**, 19 (46), 31306–31315. DOI: 10.1039/C7CP06434A.

(15) N. Zeghbib, P. Thelliere, M. Rivard, T. Martens, Microwaves and Aqueous Solvents Promote the Reaction of Poorly Nucleophilic Anilines with a Zincke Salt. *J. Org. Chem.* **2016**, 81 (8), 3256–3262. DOI: 10.1021/acs.joc.6b00208.
